# Evidence of validity of the Risk Self-Medication Questionnaire focused on Health Literacy

**DOI:** 10.1590/0034-7167-2023-0386

**Published:** 2024-07-29

**Authors:** Márcio Adriano Fernandes Barreto, Francisca Diana da Silva Negreiros, Virna Ribeiro Feitosa Cestari, Helena Alves de Carvalho Sampaio, Thereza Maria Magalhães Moreira

**Affiliations:** I Universidade do Estado do Rio Grande do Norte. Pau dos Ferros, Rio Grande do Norte, Brazil; II Universidade Federal do Ceará, Hospital Universitário Walter Cantídio. Fortaleza, Ceará, Brazil; III Universidade Estadual do Ceará. Fortaleza, Ceará, Brazil

**Keywords:** Self Medication, Risk, Health Literacy, Validation Study, Psychometrics, Automedicación, Riesgo, Alfabetización en Salud, Estudio de Validación, Psicometría

## Abstract

**Objectives:**

to analyze the validity evidence of the internal structure of the Risk Self-Medication Questionnaire Focused on Health Literacy.

**Methods:**

a psychometric study with 499 adults. The internal structure was assessed with exploratory and confirmatory factor analysis to prove the adjustment. Internal consistency was measured by composite reliability and McDonald’s omega coefficient (ω).

**Results:**

the parameters revealed a model of 35 items distributed across four factors, explaining 56% of the total variance, with factor loadings ranging from 0.31 to 0.85 and adequate communalities. Accuracy (0.79<ORION<0.98), representativeness (0.89<FDI<0.99), sensitivity (1.92<SR<7.07), factor hope (88.3%< EPTD<97.9%), replicability (0.82<H-Latent<H-observed<0.87) and reliability (ω=0.87) were adequate. The composite reliability ranged from 0.840 to 0.910. Furthermore, good model fit was achieved (TLI = 0.99; CFI = 0.99; GFI = 0.95; RMSEA = 0.02 and RMSR = 0.05).

**Conclusions:**

an instrument was obtained with good evidence of structural validity for measuring self-medication.

## RESUMEN

**Objetivos:**

analizar la evidencia de validez de la estructura interna del Cuestionario de Automedicación de Riesgos Enfocado a la Alfabetización en Salud.

**Métodos:**

estudio psicométrico, con 499 adultos. La estructura interna fue evaluada con análisis factorial exploratorio y confirmatorio para comprobar el ajuste. La consistencia interna se midió mediante la confiabilidad compuesta y el coeficiente omega de McDonald (ω).

**Resultados:**

los parámetros revelaron un modelo de 35 ítems distribuidos en cuatro factores, explicando el 56% de la varianza total, con cargas factoriales que oscilaron entre 0,31 y 0,85 y comunalidades adecuadas. Los indicadores precisión (0,79<ORION<0,98), representatividad (0,89<FDI<0,99), sensibilidad (1,92<SR<7,07), factor esperanza (88,3%< EPTD<97,9%), replicabilidad (0,82<H-Latente<H -observado<0,87) y la confiabilidad (ω=0,87) fueron adecuadas. La confiabilidad compuesta osciló entre 0,840 y 0,910. Además, se logró un buen ajuste del modelo (TLI = 0,99; CFI = 0,99; GFI = 0,95; RMSEA = 0,02 y RMSR = 0,05).

**Conclusiones:**

se obtuvo un instrumento con buena evidencia de validez estructural para medir la automedicación.

## INTRODUCTION

Risk self-medication (RSM) is anchored in the definition of self-medication of the World Health Organization (WHO)^(1)^, incorporating potential elements of risk to human health, consisting of elements involved in inappropriate self-medication^(2)^. Therefore, predicting RSM involves recognizing relevant constituent elements, such as health literacy (medication literacy)^(3-4)^, behavior and behavioral intention^(5)^. From this perspective, literacy is focused on the skills of searching, analyzing and applying information in contexts of medication use^(6)^, impacting the decision and practice of self-medication.

The context of the practice of inappropriate self-medication results in economic waste, damage to the health service and the development of antimicrobial-resistant bacteria^(7)^. Thus, there are potential elements of risk to self-medication which, according to the WHO, are related to wrong self-diagnosis, incorrect choice of therapy, failure to recognize adverse effects, drug interactions, contraindications, inadequate storage, error in dose^(2)^, in addition to not reading the labels^(8-9)^ and medication leaflets before use^(9)^. Studies show that people with low medication literacy are more likely to practice inappropriate or RSM^(4,9)^.

Therefore, measuring the risks that self-medication can cause to the population is a relevant task for public health^(10)^. At this core, research carried out by the United Nations (UN) estimates that, by 2050, these risks could result in the annual deaths of ten million people^(11)^. Brazil accounts for 35% of total medication sales in the country^(12)^, being the fifth country that consumes the most medications in the world^(13)^. Added to this is increased self-medication in the last eight years (from 72% to 81% in 2022)^(14)^. This practice has a relevant share in drug poisoning, with a fatality rate of 0.25%^(15)^.

Regarding the validity evidence process, content validity is an important step, which assesses the agreement between items and construct^(16)^, obtaining a valid and reproducible parameter. However, even if the instrument has presented satisfactory evidence at this stage, it is necessary to analyze the internal structure’s evidence of validity.

Even though the literature shows a relationship between self-medication and health literacy, to date, there have been few efforts to build and validate an instrument that measures self-medication from the perspective of health literacy. Therefore, the search for evidence of validity of an instrument that allows measuring aspects related to RSM becomes opportune to strengthen articulation, monitoring and assessment mechanisms aimed at promoting the rational use of medications^(17)^, in addition to promoting policies that can contribute in a direct and targeted way to improving healthcare services, with a view to minimizing damage caused by inadequate practice of self-medication.

## OBJECTIVES

To analyze the validity evidence of the internal structure of the Risk Self-Medication Questionnaire Focused on Health Literacy (QAR-LS - *Questionário da Automedicação de Risco Focado no Letramento em Saúde*).

## METHODS

### Ethical aspects

The project of this study was approved by the *Universidade Estadual do Ceará* Research Ethics Committee in 2022, in accordance with recommendations of Resolution 466/2012 of the Brazilian National Council of Health. Participants signed the Informed Consent Form (ICF).

### Study design, period and place

This is a psychometric study, focused on analyzing evidence of QAR-LS internal structure^(16)^, developed with the population registered in primary health institutions (n=12), who sought care in Primary Care in the city of Pau dos Ferros, Rio Grande do Norte (RN), Brazil.

The research was carried out in all (n=12) Primary Health Care health units, covering rural and urban locations. The units are from different territories of the municipality, presenting different socioeconomic strata, ranging from vulnerable situations to more favorable conditions, encompassing people with different levels of health literacy.

### Population, sample; inclusion and exclusion criteria

Adults and older adults registered in primary health institutions, selected by convenience, participated in the study. Included were people monitored in health institutions aged > 18 years and who sought out the institution to provide Primary Care services. People without cognitive conditions to respond were excluded.

The sample size of this psychometric study was based on the number of items, with a minimum proportion of ten participants for each item^(18)^. Therefore, QAR-LS with 49 items had an estimated sample size of 490 participants. When considering the extension of the general item bank, and with the intention of preserving heterogeneity and obtaining respondents that covered the entire construct, 536 people were invited (490 + 10%).

However, 36 subjects did not accept to participate and one participant was lost due to incomplete responses to items. This resulted in 499 participants, who guaranteed an average of 10.2 observations for each item of the instrument. Therefore, the number of participants was adequate based on recommendations in the literature^(18)^.

### Study protocol

The data in this study originate from a doctoral thesis. In a previous stage, the construction and content validity of the Risk Self-Medication Questionnaire (QAR - *Questionário de Automedicação de Risco*) took place^(19)^. To this end, the WHO definition of self-medication was adopted, incorporating the potential risk elements of self-medication^(1-2)^. To understand the constituent elements of self-medication, an integrative review was carried out that assessed instruments with evidence of validity that measure self-medication in Brazil, arriving at the following elements: medication literacy; behavioral intention; and behavior.

Then, the item bank was developed based on two scope reviews, involving the elements of health literacy, more specifically medication literacy^(6,20)^ and the Theory of Planned Behavior, to assist with the behavioral intention and behavior components self-medication itself, which poses risks to human health. The database resulted in 136 items, which went through the content validity process, in two rounds with judges from various regions of Brazil, who had expertise in the area of medication use and instrument validity. After this process, QAR was considered validated and reliable, with 49 items and three constituent elements (medication literacy, behavioral intention and behavior)^(19)^.

In this phase, the instrument’s internal structure was assessed, applying the instrument to a population sample (adults and older adults). The main author and five nursing students, previously trained by the main author, participated in collection. In data collection, a questionnaire with sociodemographic data (sex, age, income, education, profession), a questionnaire with clinical variables (health problem, use of continuous medications and amount of medication use without a medical prescription in the last three months) and the bank composed of 49 items, with a response pattern using a five-point Likert scale, ranging from 1 to 5 (from never to always), were used.

Data were collected from February to May 2023, in closed individual interviews, carried out in primary health units. Patients were contacted and the research objectives and relevance were explained to them. Those who agreed to participate in the study signed the ICF in two copies. Patients were then taken to a private room to ensure privacy.

### Analysis of results, and statistics

In the analysis of sociodemographic and clinical data of research participants, absolute and relative frequencies were calculated for categorical variables, and measures of central tendency and dispersion for numerical variables, depending on normality, verified by the Kolmogorov-Smirnov test.

The internal structure of QAR-LS was validated by Exploratory Factor Analysis (EFA) and Confirmatory Factor Analysis (CFA). Data adequacy was performed using the Kaiser-Meyer-Olkin (KMO) test and Bartlett’s test of sphericity (BTS), with expected values for KMO>0.60 and BTS of p<0.05^(21)^. Afterwards, the instrument dimensionality was verified by parallel analysis, via the Parallel Analysis Optimal Implementation technique^(22)^, with bootstrap association extrapolated to 1,000 cases^(23)^.

Factor extraction was performed using the Robust Unweighted Least Squares (ULS) method, with polychoric correlation^(24)^ and Robust Promin rotation^(25)^. Furthermore, the Unidimensional Congruence ((UniCo) > 0.95), Explained Common Variance ((ECV) > 0.85) and Mean of Item Residual Absolute Loadings ((MIREAL) < 0.30) techniques were used to test the dimensionality of factors^(26)^.

The factors were assessed for accuracy (Overall Reliability of fully Informative prior Oblique N-EAP scores (ORION) > 0.70), representativeness of the latent trait and effectiveness of factor estimation (Factor Determinacy Index (FDI) > 0.80^(27)^, sensitivity (Sensitivity Ratio (SR) > 2.0), expected percentage of the factor (Expected Percentage of True Differences (EPTD) > 90%) and replicability (Generalized G-H Index > 0.80)^(26)^.

Items with a correlation above 0.2 with two other items were maintained, with communalities (h^2^) and factor loadings above 0.40 and 0.30, respectively. Items with Heywood cases and double saturation were excluded^(18)^. It was also observed the convergence of the polychoric matrix, kurtosis and asymmetry.

For CFA, model fit indices were assessed: chi-square ratio by degrees of freedom (X2/gl≤5.0); Tucker-Lewis Index (TLI > 0.90); Comparative Fit Index (CFI > 0.94); Goodness of Fit Index (GFI) ≥ 0.95; Root Mean Square Error Approximation (RMSEA) ≤ 0.07; Root Mean Square of Residuals (RMSR < 0.08); Adjusted Goodness of Fit Index (AGFI) ≥ 0.93; and chi-square < 0.05^(28)^.

Finally, reliability was calculated using McDonald’s Omega Coefficient () and composite reliability (CF). The reference values adopted for and CF were: <0.6, low; between 0.6 and 0.7, moderate; and between 0.7 and 0.9, high^(18)^.

To carry out statistical analyses, the Statistical Package for the Social Sciences (IBM SPSS) version 23, Factor (version 11.05.01) and R (version 3.6.2) were used. CF was calculated using the Composite Reliability Calculator, via the website www.thestatisticalmind.com.

## RESULTS

Among the 499 research participants, there was a predominance of female people (367; 73.5%), aged between 18 and 89 years old (Md[p25-p75]=41[30.0-52.0]), with a partner (256; 51.3%), with varied education, such as incomplete (70; 14%) and complete elementary school (47; 9.4%), incomplete (27; 5.4%) and complete high school (203; 40.7%), higher education (37; 7.4%) and graduate education (18; 3.6%). The self-reported income reported by participants ranged from zero to 15 minimum wages, with a prevalence of one wage (210; 42.1%).

Regarding clinical and behavioral data, 238 (47.7%) had some health problem, with 213 (42.7%) having a chronic illness and 237 (47.5%) taking continuous medication. Polypharmacy was reported by 103 (20.6%) of participants. The maximum number of days that self-medicated in the last three months was 90, with a median of five days (Md[p25-p75]=5[2.0-15.0]).

Model 1, with all items (49) of QAR-LS, presented a negative matrix and inadequate percentage of destruction (46.3%), unacceptable KMO, as ten did not saturate and four had loads on more than one factor. Therefore, 14 items (7, 10, 11, 22, 28, 29, 30, 34, 35, 40, 44, 46, 47 and 49) were excluded and a new analysis was carried out.

**Figure 1:**
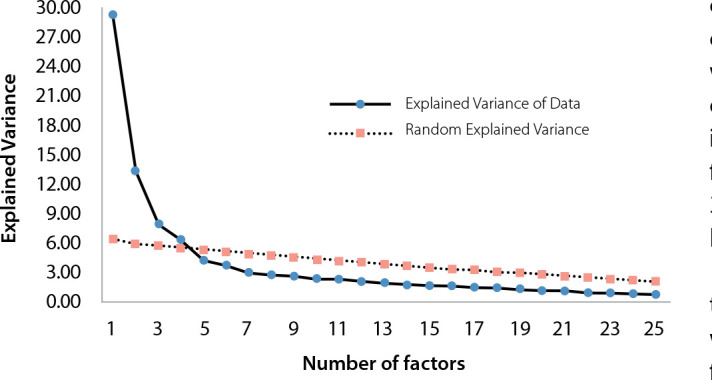
Scree plot of items from the Risk Self-Medication Questionnaire Focused on Health Literacy (QAR-LS), obtained by parallel analysis, Fortaleza, Ceará, Brazil, 2023

Model 2 revealed a positive matrix and good adequacy (KMO=0.83[0.72-0.89]; BTS=5589.5, df=595, p<0.001). Using scree plot (Figure 1) of parallel analysis, item exploration demonstrated a structure with four dominant factors (verticalized line), in which the first two factors are responsible for the largest explained variance in the data (F1=28.3%; F2=13.4%; F3=7.9%; and F4=6.4%; The UniCo (0.79 [95%CI = 0.78-0.82]), ECV (0.67 [95%CI = 0.65-0.72]) and MIREAL (0.25 [95%CI = 0.22-0.28]) attested to model multidimensionality.

Table 1 details the factors and descriptive results of QAR-LS. The first factor (04 items - 6, 12, 17 and 37) encompassed aspects related to the subjective norm, influence of other people, such as family, neighbors and friends, in decision-making, opinion, recommendation and reason for practicing self-medication. The second factor (06 items - 23, 24, 41, 42, 43 and 45) involves items referring to the possibility and execution of the practice of changing the course of treatment prescribed by a healthcare professional. The third (07 items - 1, 2, 3, 4, 5, 13 and 14) was composed of items concerning medication literacy issues, considering a person’s abilities to use medication correctly. The items are inversely proportional to the RSM construct. The fourth factor (18 items - 8, 9, 15, 16, 18, 19, 20, 21, 25, 26, 27, 31, 32, 33, 36, 38, 39 and 48) contained items related to attitude and RSM’s behavior. CF indices were also reported.

**Table 1:** Factor loadings, communalities, kurtosis and reliability of the final model with four factors, Fortaleza, Ceará, Brazil, 2023

Items	Factor loadings				K	h^2^
	F1	F2	F3	F4		
1			0.759		-1.482	0.599
2			0.638		-0.878	0.430
3			0.567		-0.757	0.342
4			0.850		-1.529	0.712
5			0.806		-1.557	0.648
6	0.739				-1.380	0.503
8				0.529	0.324	0.265
9				0.317	-1.335	0.277
12	0.728				-1.379	0.469
13			0.720		-0.999	0.533
14			0.676		-1.035	0.492
15				0.746	-0.436	0.507
16				0.784	0.271	0.516
17	0.784				-1.080	0.688
18				0.614	-0.984	0.477
19				0.737	-0.702	0.508
20				0.408	-0.645	0.304
21				0.580	-1.160	0.386
23		0.770			-1.336	0.511
24		0.588			-0.350	0.380
25				0.718	-0.925	0.442
26				0.760	-0.390	0.562
27				0.494	-1.185	0.370
31				0.674	-1.074	0.470
32				0.455	-1.363	0.274
33				0.729	-0.681	0.322
36				0.735	-1.102	0.570
37	0.763				-1.167	0.718
38				0.522	-1.051	0.376
39				0.465	-1.010	0.289
41		0.792			-0.085	0.621
42		0.547			1.233	0.391
43		0.806			-1.436	0.651
45		0.763			-0.518	0.594
48				0.435	-1.198	0.230
CF	0.840	0.862	0.883	0.910		

*F1 - influence of third parties; F2 - subject interference in therapy; F3 - medication literacy; F4 - attitudes and behaviors; K - kurtosis; h^2^ - commonalities; CF - composite reliability.*

The items presented adequate factor loadings, with high factor loadings on the respective factors. No cross-loading pattern was found (items with factor loadings >0.300 on more than one factor). The factors showed high reliability, according to CF values (F1=0.840; F2=0.862; F3=0.883 and F4=0.910, and =0.87).

Table 2 specifies the quality and effectiveness of QAR-LS factor score estimates. The questionnaire proved to be adequate in terms of accuracy (0.79 < ORION < 0.98), representativeness (0.89 < FDI < 0.99), sensitivity (1.92 <SR<7.07), factor expectation (88.3%< EPTD<97.9%) and repeatability (0.87<H-Latent< 0.94) (0.82 < H-observed<0.91). All factors were adequate, according to reference values, attesting to the quality.

**Table 2:** Quality of factor estimates from the Risk Self-Medication Questionnaire Focused on Health Literacy (QAR-LS - *Questionário da Automedicação de Risco Focado no Letramento em Saúde*), Fortaleza, Ceará, Brazil, 2023

Factors	ORION	FDI	SR	EPTD (%)	G-H Index	
					H-latent	H-observed
Influence of third parties	0.870	0.933	2.591	91.2	0.87 (0.28-0.89)	0.82 (0.75-0.87)
Subject interference in therapy	0.886	0.941	2.792	91.9	0.88 (0.85-0.91)	0.80 (0.73-0.87)
Medication literacy	0.904	0.951	3.073	92.7	0.90 (0.86-0.92)	0.83 (0.75-0.86)
Attitudes and behaviors	0.930	0.965	3.654	94.1	0.93 (0.92-0.94)	0.90 (0.88-0.91)

Table 3 shows the fit indices of tested models and reveals the quality of model 2 over the others, expressed by the values in EFA and CFA.

**Table 3:** Model fit indices, Fortaleza, Ceará, Brazil, 2023

Estimates	Value	95%CI	Reference values
X^2^/gl	1.83	-	≤ 5.0
Tucker-Lewis Index	0.985	0.986-0.992	> 0.90
Comparative Fit Index	0.988	0.989-0.994	> 0.94
Goodness of Fit Inde	0.951	0.946-0.961	> 0.95
Adjusted Goodness of Fit Index	0.937	0.931-0.950	> 0.93
Root Mean Square Error of Approximation	0.027	0.019-0.027	< 0.07
Root Mean Square of Residuals	0.052	0.048-0.052	< 0.08

Considering the results obtained, Chart 1 presents the final version, after factor analysis, in which QAR-LS presents 35 items divided into four dimensions (influence of third parties; interference of subjects in therapy; medication literacy; and attitude and behaviors).

**Chart 1:** Final version of the Risk Self-Medication Questionnaire Focused on Health Literacy (QAR-LS - *Questionário da Automedicação de Risco Focado no Letramento em Saúde*), Fortaleza, Ceará, Brazil, 2023

	QAR-LS (*Questionário de Automedicação de Risco focado no Letramento em Saúde*)
**Influence of third parties**	
1	Before using medication on my own, I seek information from friends/family.
2	When using medication on my own, in the presence of a drug reaction, I seek information from family/friends.
3	I intend to take medication on my own because I trust the opinion of friends/neighbors/family.
4	I use medication on my own because it is recommended by friends/neighbors/family.
**Subject interference in therapy**	
5	I would intend to discontinue use of the medication prescribed by the doctor if symptoms improved.
6	I would intend to suspend the use of the medication prescribed by the doctor and use it at another time.
7	I reduce the dose of the medication the doctor or nurse prescribed when symptoms improve.
8	I increase the dose of the medication the doctor or nurse prescribed when I realize that I am not improving.
9	I stop using medication on my own when I feel better.
10	I have already reduced the number of days of treatment.
**Medication literacy**	
11	Before using medication on my own, I read the information in the leaflet.
12	Before using medication on my own, I read the information on the label.
13	Before using medication on my own, I assess the appropriate dose to be taken.
14	Before using medication on my own, I ask my questions in the leaflet.
15	Before using medication on my own, I follow the instructions contained in the leaflet regarding the number of days to use the medication.
16	When taking medication on my own, I know how to calculate the dose.
17	Before using medication on my own, I understand the information contained in its leaflet.
**Attitudes and behaviors**	
18	Before using medication on my own, I follow my previous experience with the same medication.
19	Before using medication on my own, I follow previous medical prescriptions.
20	I intend to take medication on my own because it relieves the symptoms quickly.
21	I intend to take medication on my own because it has the desired effect.
22	I intend to take medication on my own because I do not need a medical appointment.
23	I intend to take medication on my own because I have it at home.
24	I intend to take medication on my own even without knowing how to identify adverse reactions.
25	I intend to take medication on my own even without medical, pharmacist or nurse advice.
26	I have taken medication on my own for the last three months.
27	I use medication on my own when I do not need a prescription from the pharmacy.
28	I use medication on my own even when prescription retention is required at the pharmacy.
29	I use medication on my own because I have previous treatment experience.
30	I use medication on my own because I have old prescriptions.
31	I use medication on my own because I have medication stored at home.
32	I use medication on my own because it is quicker to solve my health problems.
33	I use medication on my own because I do not need guidance from healthcare professionals.
34	I use more than one medication on my own at the same time.
35	I use antibiotics on my own.

The assessed indicators indicate a four-dimensional model with consistent internal evidence for measuring the RSM construct.

## DISCUSSION

Self-medication assessment instruments provide information relevant to understanding the prevalence of self-medication in the population. However, the development of standardized self-report metrics, sensitive to RSM knowledge, intention and behavior, expands the understanding of the elements of risk to subjects’ health and helps the health service to use effective interventions that can minimize damage from inappropriate self-medication to human health^(29)^.

QAR-LS proves to be a useful and advantageous screening tool, as it considers the influence of third parties, interference of subjects in therapy, literacy and attitude and behaviors, and can be used in health service strategies to minimize potential elements of risk to the population’s health. To minimize health risks and ensure safe use of medications, it is necessary for the individual to have knowledge, attitude and appropriate practice regarding the use of over-the-counter medications^(30)^.

The results of this study demonstrated that QAR-LS presents multidimensional characteristics, satisfactory factor loadings and good levels of reliability, which point to an instrument with consistent and reliable internal evidence to measure the desired construct. To date, this is the first study to assess RSM validity, from the perspective of health literacy (medication literacy) and the Theory of Planned Behavior, using various sizing techniques and adjustment indices.

It is reinforced that the literature has demonstrated a relationship between the inadequate practice of self-medication and low medication literacy^(9,31)^, just as it has been demonstrated that the Theory of Planned Behavior (attitude, subjective norm and perceived control) constructs are strong predictors of self-medication^(32-33)^.

Validity based on the internal structure of an instrument represents the degree to which the structure of correlations between items corresponds to the construct structure that the test proposes to measure^(34)^. Therefore, assessing evidence of the internal structure of instruments is a complex procedure that requires the execution of requirements, such as instrument factorial structure and reliability, which are crucial for it to be effective^(16)^. In this study, the instrument empirical structure reflected the construct theoretical structure, and the analyzed sample proved to be adequate and representative for continuing factor analysis, according to evidence from BTS, KMO and RCI.

Factor analysis identified that the RSM construct is organized into four factors, evidenced by the factor analysis correlation matrix as congruent with the definition of RSM. The theoretical structure consisted of three factors: medication literacy; behavioral intention; and behavior. Thus, by regrouping the items in factor analysis, the four factors were redefined as: influence of third parties; interference of subjects in therapy; medication literacy; and attitude and behaviors.

The four factors, evidenced by the factor analysis correlation matrix, were congruent with the definition of RSM and the respective constituent elements: RSM is multifactorial and incorporates behavioral attributes, behavioral intention (attitude, subjective norm and perceived control) and medication literacy. This model breaks with the traditional one, seeking to measure only self-medication in general. In this way, RSM focused on health literacy is measured at levels that can adopt preventive and promotional measures that precede the risk of self-medication.

Factor 1 contains items from the three theoretical dimensions of the RSM construct; In this factor, the Theory of Planned Behavior is anchored, in a more preponderant way, with the subjective norm construct, which is the belief about the social pressure exerted on an individual to perform or not perform a certain behavior and a person’s motivation to agree with this pressure^(35)^. This pressure from third parties involves people who influence the decision, motivation and practice of self-medication, such as family members^(36-37)^ and friends^(5,38-39)^. This is reinforced by the fact that a study based on the Theory of Planned Behavior showed that 69% of spouses or friends influenced the taking of medications without prescriptions^(5)^.

Factor 2 theoretically corresponds to medication literacy, behavioral intention and behavior in interfering with the conduct of therapy, presenting the three dimensions of the theoretical model. It should be noted that self-medication is a process that involves several facets, such as the WHO definition of self-medication^(1)^ as intermittent use, when interruption/interference in the conduct of the treatment prescribed by a clinician occurs. The behavioral intention and practice of carrying out this situation, combined with the below literacy, constitutes a potential health risk. Therefore, interruption of treatment, such as the use of antibiotics, can contribute to bacterial resistance^(40-41)^.

Factor 3 refers to items considered exclusively from medication literacy, such as reading and understanding medication labels and leaflets, to ensure safe use of dose, duration and other aspects that guarantee patient safety. In this way, these items are configured as inverse to the RSM construct, whose people who score low on the Likert scale will be prone to greater risk when self-medicating.

Factor 4, RSM behavioral intention and behavior, involves subjects’ motivation to carry out a certain behavior, such as packaging medications at home or even the practice itself of having already stored medications in other situations, how to use antibiotics on their own and not seek information from healthcare professionals. These elements are potential risks when using the medication on their own.

The factors demonstrated strong internal consistency, according to the CF value. In this study, we chose to use CF, as it is a technique that considers the factorial loading of items, therefore, allowing a better assessment of the quality of the structural model of psychometric instruments^(42)^.

The adjustment adequacy indices were satisfactory, suggesting acceptance of the model. Thus, the use of robust techniques carried out by confirmatory factor analysis, such as accuracy, representativeness, sensitivity, replicability, expected percentage of the factor and model adequacy indices, in addition to the use of these indicators, combined with theoretical interpretation, made it possible to eliminate possible biases.

The instrument can help fill a gap related to the scarcity of other forms of assessing actions focused on RSM. Furthermore, it can help health workers to develop interventions, being able to act in different spheres, in order to promote the population’s health.

### Study limitations

However, some limitations include possible cultural factors that may impact the understanding of items, subjectivity in participants’ responses and non-generalization to different populations, making it important to carry out future studies that involve participants from other regions, with other cultures and other behaviors. Furthermore, the research brings robust techniques based on psychometric recommendations.

### Contributions to nursing, health or public policy

This study offers a valuable instrument to fill the gap in RSM assessment, providing a tool that can guide healthcare professionals in the development of interventions aimed at specific groups. The instrument highlights aspects such as medication literacy, behavioral intention and decision-making, addressing attitudes, subjective norms and perceived control related to RFM practice. This makes it possible to assess RSM knowledge, decision-making and practice, allowing targeted and effective interventions to promote the health of those at risk in relation to self-medication.

## CONCLUSIONS

The QAR-LS, composed of 35 items, distributed across four dimensions (influence of third parties, interference of subjects in therapy, medication literacy and attitude and behaviors), presented satisfactory psychometric properties, demonstrating a model with good evidence of validity and reliability.

## Supplementary Material





## Data Availability

*https://doi.org/10.48331/scielodata.WPYUK2*
