# Supplementary material for: Evidence of validity of the Risk Self-Medication Questionnaire focused on Health Literacy
Source: Rev Bras Enferm. 2024 Jul 29;77(3):e20230386. doi: 10.1590/0034-7167-2023-0386 (PMC11290745; doi:10.1590/0034-7167-2023-0386)
Supplement: Supplementary file 1 [file 0034-7167-reben-77-03-e20230386-suppl01.pdf]

- Título do conjunto de dados: Banco de dados do artigo intitulado "Evidências de validade do Questionário da Automedicação de Risco focado no Letramento em Saúde (QAR-LS)"
- Informações de contato: Márcio Adriano Fernandes Barreto, Universidade do Estado do Rio Grande do Norte, marciofernandes@uern.br
- Data de coleta dos dados: fevereiro a maio de 2023.
- Visão geral dos dados e arquivos: O arquivo referente ao banco de dados do estudo estão salvos no formato PDF. Trata-se de um banco de dados quantitativos com as variáveis obtidas durante a coleta de dados do estudo. Tais dados foram o substrato para a análise quantitativa, que envolveu testes estatísticos, incluindo validade da estrutura interna do instrumento por meio de análise fatorial exploratória e confirmatória, teste de esfericidade de Bartlett, índice Kaiser-Meyer-Olkin, para a retenção dos fatores utilizou-se a Parallel Analysis Optimal Implementation. O arquivo foi criado em junho de 2023, concomitantemente com o final da coleta de dados.
- Descrição dos métodos de coleta ou geração dos dados: os dados foram gerados a partir da etapa de validação da estrutura interna do QAR-LS, por aplicação com o público-alvo. Participaram 499 sujeitos com idade igual ou maior a dezoito anos, sendo os dados coletados de forma presencial e individual. A estrutura interna do QAR-LS foi validada pela Análise Fatorial Exploratória (AFE) e Confirmatória (AFC).
- Descrição dos métodos usados para o processamento dos dados: 1) elaboração dos bancos de dados no software Excel. 2) Análise estatística dos dados no programa Para realização das análises estatísticas, os softwares Statistical Package for the Social Sciences (IBM SPSS), versão 23; Factor (versão 11.05.01) e R (versão 3.6.2). O cálculo da FC ocorreu pela Composite Reliability Calculator, via site www.thestatisticalmind.com (adotou-se o nível de significância 5% para todos os testes estatísticos). Testes realizados: análise fatorial exploratória (AFE), Análise Paralela via técnica Parallel Analysis Optimal Implementation, análise fatorial confirmatória (AFC), teste de esfericidade de Bartlett, índice Kaiser-Meyer-Olkin,Coeficiente Ômega de McDonald ( ) e fidedignidade composta (FC).
- Informações específicas dos dados: As variáveis do QAR-LS, apresentam resposta de 1 a 5, onde 1 = nunca, 2 = quase nunca, 3 = as vezes, 4 = quase sempre e 5= sempre.
