# Supplementary material for: Evidence of validity of the Risk Self-Medication Questionnaire focused on Health Literacy
Source: Rev Bras Enferm. 2024 Jul 29;77(3):e20230386. doi: 10.1590/0034-7167-2023-0386 (PMC11290745; doi:10.1590/0034-7167-2023-0386)
Supplement: Supplementary file 2 [file 0034-7167-reben-77-03-e20230386-suppl02.pdf]

Nº	sexo	renda	idade	estado civil	escolaridade	problema de saúde	DCNT	medicamento continuo	polifarmaco >2 fármcaos
1	2	1	32	1	4	2	2	2	2
2	2	3	55	1	4	1	1	1	1
3	1	1	49	2	4	2	2	2	2
4	1	1	32	2	5	1	1	1	2
5	2	1	30	1	5	2	2	2	2
6	2	1	50	4	4	1	1	1	1
7	2	3	23	3	5	2	2	2	2
8	2	0	42	2	1	2	2	2	2
9	2	2	30	1	5	2	2	2	2
10	2	1	20	1	4	1	1	1	2
11	2	0	24	1	5	2	2	1	2
12	2	4	49	1	6	2	2	2	2
13	2	2	58	2	4	1	1	1	2
14	2	1	65	1	4	1	1	1	1
15	2	2	44	3	5	2	2	2	2
16	1	1	25	1	1	2	2	2	2
17	2	2	40	2	6	1	1	2	2
18	2	0,5	43	2	4	2	2	2	2
19	2	1	50	2	1	1	1	1	2
20	2	1	30	1	4	1	1	1	2
21	1	1	26	1	1	2	2	2	2
22	2	1	34	1	4	1	1	1	1
23	2	2	37	1	5	1	1	1	1
24	1	1	28	1	4	2	2	2	2
25	2	1	37	4	5	2	2	2	2
26	2	0	21	1	4	2	2	2	2
27	1	1	21	1	4	2	2	2	2
28	2	1	22	1	4	2	2	2	2
29	2	0	20	4	2	2	2	2	2
30	2	0	53	5	1	1	1	2	2
31	2	0	41	2	1	1	1	1	2
32	2	1	39	2	4	1	1	1	1
33	1	2	42	1	4	2	2	2	2
34	2	1	18	1	3	2	2	2	2
35	1	0	18	1	3	2	2	2	2
36	1	3	45	3	4	1	1	1	2
37	2	0,6	24	1	5	2	2	2	2
38	1	0	26	4	2	2	2	2	2
39	1	3	40	6	2	2	2	2	2
40	1	1	37	2	4	1	1	1	2
41	1	2	29	3	2	2	2	2	2
42	1	2	36	5	2	2	2	2	2
43	2	0	45	2	4	1	1	1	2
44	2	1	38	1	4	1	1	1	2

45	1	0	28	1	4	1	1	1	2
1	2	2	30	1	4	2	2	1	2
2	2	1	44	1	4	1	2	1	2
3	2	0	25	1	3	2	2	2	2
4	2	1	37	2	2	1	1	1	2
5	2	2	38	2	4	2	2	1	2
6	2	2	40	2	5	5	1	1	2
7	2	3	45	3	5	2	2	2	2
8	1	1	34	3	4	2	2	2	2
9	1	2	53	6	1	1	1	1	2
10	1	1	39	2	3	2	2	2	2
11	1	1	28	3	5	2	2	2	2
12	2	1	80	2	2	1	1	1	2
13	2	1	25	4	1	2	2	2	2
14	2	0	37	1	4	2	2	1	2
15	2	1	55	2	4	1	1	1	2
16	2	1	60	6	2	1	1	1	1
17	2	1	26	1	2	1	1	2	2
18	2	1	60	2	4	1	1	1	1
19	1	4	45	3	4	1	1	1	1
20	1	1	46	2	1	2	2	2	2
21	1	1	43	3	4	2	2	2	2
22	2	1	41	3	4	2	2	2	2
23	2	1	64	5	2	1	1	1	1
24	2	1	68	6	1	1	1	1	1
25	2	0	23	1	4	2	2	2	2
26	2	3	55	5	4	1	1	1	2
27	2	3	24	1	4	2	2	2	2
28	2	2	25	3	4	2	2	1	2
29	1	3	49	2	4	2	2	2	2
30	2	1	20	1	1	2	2	2	2
31	2	1	58	2	2	1	1	1	2
32	2	1	57	1	5	1	1	1	1
33	2	4	30	1	5	2	2	2	2
34	2	0	35	2	1	1	1	2	2
35	1	1	61	2	1	1	2	1	2
36	2	2	35	2	5	2	2	1	2
37	2	0	22	3	1	2	2	1	2
38	2	0	53	6	1	2	2	2	2
39	2	3	40	5	4	2	2	1	2
40	2	0	47	2	2	1	1	1	1
41	2	1	51	2	1	1	1	1	2
42	2	1	46	2	1	2	2	2	2
43	2	1	60	5	1	1	1	1	1
44	1	1	73	2	1	1	1	1	1
45	2	0	50	2	1	1	1	1	2
46	2	4	45	1	5	2	2	2	2
1	1	2	27	1	5	2	2	2	2
2	2	1	52	2	2	1	1	1	1
3	2	2	52	1	4	2	2	2	2

4	2	2	57	2	4	1	1	1	1
5	2	1	48	2	1	2	2	2	2
6	2	1	36	1	4	1	1	1	1
7	2	2	33	2	6	2	2	2	2
8	1	5	36	2	5	2	2	2	2
9	1	1	57	1	2	2	1	1	1
10	2	1	36	1	4	2	2	1	2
11	2	0	38	5	4	1	1	1	2
12	2	0	26	2	3	2	2	2	2
13	2	0	22	1	2	1	1	1	2
14	2	1	25	1	4	2	2	2	2
15	2	1	55	1	1	2	2	2	2
16	2	1	51	1	3	1	1	1	2
1	2	3	45	2	4	1	1	1	2
2	2	1	22	1	4	2	2	2	2
3	2	1	22	3	4	2	2	1	2
4	2	1	26	2	4	2	2	2	2
5	1	1	25	2	5	2	2	2	2
6	1	3	30	1	4	1	2	2	2
7	2	1	35	1	5	2	2	2	2
8	2	2	33	2	6	1	1	1	2
9	2	3	28	2	5	2	2	2	2
10	2	1	41	5	6	1	1	1	1
11	2	1	58	4	1	1	1	1	1
12	2	1	54	2	4	1	2	2	2
13	1	3	46	2	2	2	2	2	2
14	2	2	68	6	2	2	2	2	2
15	2	0	43	2	2	2	2	2	2
16	2	1	65	5	4	1	1	1	2
17	1	1	28	1	4	1	1	1	1
18	2	1	44	5	3	1	1	1	2
19	2	1	35	2	4	1	2	2	2
20	2	1	28	1	5	2	2	2	2
21	2	1	50	2	4	1	1	1	1
22	2	1	29	1	4	1	1	1	2
23	1	1	44	2	5	2	2	2	2
24	1	3	35	2	5	2	2	2	2
25	1	1	58	6	2	2	2	2	2
26	2	0	24	1	4	2	2	1	2
27	2	0	50	2	4	1	1	2	2
28	2	1	56	6	2	1	1	1	1
29	2	1	34	2	4	1	1	1	2
30	2	0,5	18	4	2	2	2	2	2
31	2	1	50	1	4	2	2	2	2
32	2	0	21	1	4	2	2	1	2
33	2	4	56	5	5	1	1	1	2
34	2	0	26	1	4	1	1	2	2
35	1	0	19	1	4	2	2	2	2
36	2	0	35	3	4	2	2	2	2
37	1	5	36	2	6	2	2	2	2

38	1	1	56	5	1	2	2	2	2
39	2	4	39	2	6	2	2	1	2
1	2	6	74	2	5	1	1	1	1
2	2	1	42	2	4	2	2	2	2
3	2	1	74	2	2	1	1	2	2
4	2	1	62	1	2	1	1	1	1
5	2	1	53	2	4	2	2	2	2
6	2	1,5	25	2	5	2	2	1	2
7	2	2,5	54	2	4	1	1	1	2
8	1	2,5	49	2	4	1	1	1	2
9	1	0,5	19	1	4	2	2	2	2
10	2	0	19	1	4	2	2	2	2
11	2	1	46	1	4	2	2	2	2
12	1	1	52	1	1	1	1	1	2
13	1	2	65	1	2	1	1	1	2
14	2	2	27	1	6	2	2	2	2
15	2	2	47	1	4	1	2	2	2
16	2	3	47	5	5	1	1	1	1
17	2	1	48	2	4	2	2	2	2
18	2	2	46	2	4	1	2	1	2
19	2	1	31	2	4	2	2	2	2
20	2	1	33	1	4	1	1	1	2
21	2	0	18	1	4	1	1	1	1
22	2	0	47	3	2	1	1	1	2
23	2	0	26	1	4	2	2	2	2
24	2	1	28	2	4	1	1	1	2
25	2	0	19	1	2	2	2	2	2
26	2	1	40	2	2	1	1	1	1
27	2	0	46	3	4	1	1	1	1
28	1	1	38	3	1	2	2	2	2
29	2	1	67	2	1	1	1	1	1
30	2	2	28	3	4	2	2	1	2
1	2	1	44	1	5	2	2	2	2
2	1	2	41	2	4	1	2	2	2
3	2	1	41	2	5	2	2	2	2
4	2	1	42	2	4	2	2	2	2
5	2	2	54	2	4	1	1	1	2
6	2	1	35	2	4	2	2	2	2
7	2	1	29	2	4	2	2	1	2
8	2	0,5	25	1	1	2	2	2	2
9	2	0,5	42	3	1	2	2	2	2
10	2	0	22	3	4	2	2	2	2
11	1	2	57	2	4	2	2	2	2
12	2	1	58	2	4	1	2	2	2
13	2	1	39	2	4	2	2	2	2
14	2	1	35	1	4	2	2	2	2
15	2	1	21	2	4	1	2	2	2
16	2	1	66	1	4	1	2	2	2
17	2	1	68	2	1	1	2	1	2
18	2	2	48	2	5	1	1	2	2

19	2	1	66	2	1	1	1	1	1
20	1	0,5	47	3	1	2	2	2	2
21	2	1	45	6	3	1	1	1	1
22	1	1	50	5	2	1	1	1	2
23	2	0,5	41	3	1	2	2	2	2
24	2	1	38	2	4	1	1	2	2
25	2	1	58	2	1	1	1	1	2
26	1	1	28	2	4	2	2	2	2
27	2	1	40	2	5	2	2	2	2
28	2	2	45	2	4	2	2	2	2
29	1	2	53	5	4	2	2	2	2
30	2	1	35	3	4	1	1	2	2
31	2	4	57	2	5	1	2	1	2
32	2	5	46	2	6	1	1	1	1
33	1	0	18	1	3	1	1	1	1
34	1	5	23	1	5	2	2	2	2
35	1	5	44	3	5	1	1	2	2
36	2	5	42	2	6	2	2	1	2
37	2	1	50	2	5	1	1	1	1
38	1	4	52	2	3	2	2	2	2
39	2	0	19	1	4	2	2	2	2
40	2	1	42	3	2	2	2	2	2
41	2	5	44	2	6	1	1	1	2
42	1	2	69	2	2	1	1	1	1
43	1	3	54	2	5	2	2	2	2
45	1	0	18	1	4	2	2	2	2
46	2	0,5	51	2	1	1	1	1	2
47	2	4	68	2	5	1	1	1	1
48	2	1	86	2	1	1	1	1	1
49	1	1	89	2	1	1	1	1	2
50	2	1	59	2	1	2	2	2	2
51	2	0,5	44	1	4	1	1	1	1
52	2	1	51	2	4	1	1	1	2
53	2	0,5	57	1	3	1	1	1	1
54	2	1	63	5	1	2	2	2	2
55	1	1	30	1	3	2	2	2	2
56	1	0	18	1	3	2	2	2	2
57	2	0,5	47	1	4	2	2	2	2
58	2	0,5	23	1	4	2	2	2	2
59	2	0,5	32	3	1	1	2	2	2
1	2	1	29	2	4	2	2	2	2
2	2	2	51	5	4	1	1	2	2
3	2	1	34	2	5	2	2	2	2
4	2	2	27	2	4	2	2	2	2
5	2	4	35	3	6	1	1	1	2
6	2	1	39	2	5	1	1	1	2
7	2	2	50	2	4	1	1	1	1
8	2	1,5	44	5	4	1	1	1	2
9	2	1	37	2	5	1	1	1	2
10	2	2	52	3	4	1	1	1	1

11	2	3	49	2	4	2	2	2	2
12	2	2	56	2	5	1	1	1	2
13	2	2	33	2	5	2	2	2	2
14	2	2,5	77	5	1	1	1	1	1
15	2	1	50	2	5	1	1	1	1
16	2	0,5	33	1	5	2	2	2	2
17	2	1	42	1	4	1	1	1	2
18	2	1	38	5	4	1	1	2	2
19	2	3	24	2	5	2	2	2	2
20	2	1	54	5	2	1	1	1	1
21	2	0,5	21	1	4	2	2	1	2
22	1	2	31	2	5	2	2	2	2
23	1	1,5	36	3	4	1	2	2	2
24	2	0,5	35	3	3	2	2	2	2
25	2	2,5	65	2	2	1	1	1	1
26	1	10	57	1	6	1	1	1	1
27	1	1	57	2	3	1	1	1	1
28	2	2	53	2	4	1	1	1	1
29	2	0	22	1	5	2	2	1	1
30	2	1	60	2	1	1	1	1	2
31	2	3	61	2	5	2	2	2	2
32	2	2	57	5	5	2	2	2	2
33	2	4	45	2	5	2	2	2	2
34	2	4	25	1	5	1	1	2	2
35	2	1	49	2	4	1	1	1	2
36	2	4	50	2	4	2	2	1	2
37	2	0	57	5	2	1	1	1	1
38	2	1	62	5	1	1	1	1	1
39	2	2	57	2	5	1	1	1	2
40	2	1	55	6	4	1	1	1	2
41	2	0,5	40	3	2	1	1	1	2
42	2	1	64	2	1	1	1	1	1
43	2	1	37	2	4	2	2	2	2
44	1	0	18	1	4	1	1	1	1
45	2	3	59	2	5	1	1	1	2
46	2	1	52	1	3	1	1	1	1
47	1	3	30	1	4	2	2	2	2
48	2	3	33	5	4	2	2	1	2
49	2	0	29	2	4	2	2	2	2
50	1	3	41	2	4	2	2	2	2
51	2	0	39	2	4	1	1	1	2
52	2	5	67	5	5	1	1	1	2
53	2	2	69	1	5	2	2	2	2
54	2	3	64	1	5	1	1	1	1
55	2	4,5	32	2	6	1	1	1	2
56	1	3	38	1	5	2	2	2	2
57	2	0	33	1	5	2	2	2	2
58	2	1	33	1	4	2	2	2	2
59	2	1	33	5	4	1	1	1	1
60	2	3	76	6	5	1	1	1	2

61	2	1	41	1	4	1	1	2	2
1	2	1	51	2	5	1	2	2	2
2	1	4	29	1	5	1	2	2	2
3	2	1	32	1	6	2	2	2	2
4	2	2	50	2	4	1	1	1	2
5	2	2	22	1	4	2	2	2	2
6	2	2	45	2	3	2	2	1	2
7	2	2	42	2	5	1	1	1	2
8	2	1	39	2	4	2	2	2	2
9	2	15	43	1	6	2	2	2	2
10	2	3	59	1	4	2	2	2	2
11	2	2	42	1	4	2	2	2	2
12	2	1	42	1	6	2	2	2	2
13	2	5	40	2	6	1	2	2	2
14	2	1,5	18	2	5	2	2	2	2
15	2	0	42	2	5	2	2	2	2
16	2	1	40	2	5	1	1	1	2
17	2	1	40	2	1	2	2	2	2
18	2	0	41	2	2	1	1	1	2
19	2	0	26	1	5	2	2	2	2
20	2	2	41	2	4	1	1	1	1
21	1	2	24	1	5	2	2	2	2
22	2	1	40	2	2	2	2	2	2
23	2	3	34	1	5	1	1	1	1
24	1	2	56	2	4	2	2	2	2
25	2	5	30	1	6	1	1	1	1
26	2	5	34	3	5	1	1	1	2
27	2	3	50	6	4	1	1	1	2
28	2	7	37	2	6	2	2	1	1
29	1	9	35	1	6	1	2	1	2
30	2	1	28	1	4	2	2	2	2
31	1	3	60	2	4	2	2	2	2
32	1	1	60	5	4	1	1	2	2
33	1	1	45	2	4	1	1	1	2
34	2	4	60	5	6	2	2	2	2
35	1	1	50	6	4	1	1	1	1
36	2	0	30	1	6	2	2	2	2
37	1	1	33	1	5	2	2	2	2
38	2	2	61	1	5	1	1	1	2
39	1	1	28	1	5	2	2	2	2
40	1	4	56	2	5	2	2	2	2
41	1	2	47	3	4	2	2	2	2
42	2	4	42	2	5	1	1	1	1
43	2	4	60	1	5	1	1	1	2
44	1	2	44	1	4	2	2	2	2
45	2	5	56	2	5	1	2	1	2
46	1	1	39	3	4	2	2	2	2
47	2	4	53	5	5	2	2	2	2
48	2	1	57	2	4	1	1	1	1
49	2	3	53	2	4	1	1	1	2

50	1	2,5	48	5	2	1	1	1	1
51	2	1	43	1	4	2	2	2	2
52	2	1	51	2	4	1	2	2	2
53	2	3	53	2	4	1	1	1	1
54	2	1	60	1	4	1	1	1	2
55	2	0	27	1	4	2	2	2	2
56	2	2	27	1	6	2	2	1	1
57	2	4	75	6	5	1	1	1	1
58	2	4	36	3	5	2	2	2	2
59	1	5	38	3	4	1	1	1	1
60	2	1	28	1	4	2	2	2	2
61	2	1	53	5	4	1	1	1	1
62	2	1	41	1	4	2	2	2	2
63	2	1	59	2	2	1	1	1	1
64	1	1	54	2	2	1	1	1	1
65	2	0	52	1	1	1	1	1	2
66	1	1	36	1	4	2	2	2	2
67	1	1	54	1	3	1	1	1	1
68	1	1	52	1	1	2	2	2	2
1	2	1	56	1	5	1	1	1	1
2	2	1	23	1	4	2	2	2	2
3	2	1	42	1	4	2	2	2	2
4	2	2	77	6	1	1	1	1	1
5	2	2	42	2	6	1	1	1	1
6	2	2	26	1	4	2	2	2	2
7	2	1,5	39	2	4	1	1	1	2
8	2	2	41	2	4	2	2	2	2
9	2	2	60	4	2	2	2	2	2
10	2	2	36	5	5	2	2	2	2
11	1	2	40	1	5	2	2	2	2
12	2	2	57	2	5	1	1	1	1
13	2	2	51	2	4	2	2	2	2
14	2	1	47	1	1	2	2	2	2
15	2	1	26	1	4	2	2	2	2
16	2	2	53	2	4	1	1	1	1
17	2	1	41	1	4	2	2	1	2
18	1	1	29	2	4	2	2	2	2
19	1	3	50	2	4	2	2	2	2
20	1	1	35	3	4	2	2	2	2
21	2	1	58	5	1	1	1	1	2
22	1	3	39	3	2	2	2	2	2
23	2	0	20	4	2	2	2	2	2
24	1	1	35	1	5	2	2	2	2
25	1	2	50	2	5	1	1	1	2
26	1	1	30	3	4	2	2	2	2
27	2	1	25	1	4	2	2	2	2
28	1	2	29	1	5	2	2	2	2
29	1	1	20	4	2	2	2	2	2
30	1	1	35	2	4	1	1	1	2
31	1	1,5	38	1	4	1	1	1	2

32	2	3	48	1	6	2	2	2	2
33	2	1	54	1	1	2	2	1	2
34	1	1	29	1	6	1	2	2	2
35	2	1	55	1	1	1	1	1	2
36	2	3	58	1	5	1	1	1	2
37	1	1	33	1	4	1	1	1	2
38	1	1	35	1	4	2	2	2	2
39	2	1	52	1	4	2	2	2	2
40	1	1	63	1	1	2	2	2	2
41	2	1	79	2	1	1	2	1	1
42	1	5	56	2	3	2	1	1	2
43	1	1	70	2	4	1	1	1	2
44	2	1	41	2	5	1	1	1	1
45	1	0	26	1	5	2	2	2	2
46	1	3	27	1	6	2	2	2	2
47	1	4	65	2	4	1	1	1	1
48	1	7	41	1	6	1	2	2	2
49	1	3	54	1	5	1	1	1	2
50	2	6	50	2	6	2	2	2	2
51	1	1	35	2	5	2	2	2	2
52	2	1	38	1	4	2	2	2	2
53	2	1	69	3	4	1	1	1	1
54	1	3	24	1	5	2	2	2	2
55	2	2	24	2	5	2	2	2	2
56	2	4	57	2	5	1	1	1	2
57	2	1	41	2	4	2	2	2	2
58	2	3	50	2	4	2	2	1	2
59	2	2	36	3	4	2	2	2	2
60	2	0	22	1	4	1	1	1	1
1	1	1,5	18	3	3	2	2	2	2
2	2	2	36	3	5	2	2	2	2
3	1	2	42	2	5	1	1	1	2
4	1	1	31	2	3	2	2	2	2
5	2	5	47	2	6	2	2	1	1
6	2	3	34	3	4	1	1	1	2
7	2	0	37	2	3	2	2	2	2
8	2	4	47	2	5	2	2	2	2
9	2	1,5	19	3	3	2	2	2	2
10	2	0	21	3	3	2	2	2	2
1	2	1	37	3	4	2	2	2	2
2	2	1	72	6	1	1	1	1	1
3	2	1	25	3	1	1	1	1	1
4	2	8	38	2	6	1	1	2	2
5	1	1	22	1	4	2	2	2	2
6	2	1	49	2	1	1	1	1	1
7	2	1	54	2	2	1	1	1	1
8	2	1	20	1	4	1	1	2	2
9	2	2	27	1	4	2	2	2	2
10	1	2	59	2	4	2	2	2	2
11	2	2	46	2	4	2	2	1	2

12	2	2	60	6	4	1	1	1	2
13	2	1	20	1	4	2	2	2	2
14	2	1	22	2	4	2	2	1	2
15	2	0,5	28	1	4	2	2	2	2
16	2	1	57	2	1	1	1	1	1
17	2	1	21	1	4	2	2	1	2
18	2	0,5	57	1	1	1	1	1	1
19	2	1	24	1	5	2	2	2	2
20	2	3	32	1	6	2	2	2	2
21	2	0,5	24	1	4	2	2	2	2
22	2	0,5	57	3	1	1	1	1	1
23	2	0,5	22	1	4	2	2	2	2
24	2	1	56	6	1	1	1	1	1
25	2	0,5	44	2	1	1	1	1	1
26	2	1	37	1	4	2	2	2	2
27	2	2	36	5	5	2	2	2	2
28	2	2	34	1	5	1	1	2	2
29	2	1	54	2	4	1	1	1	2
30	2	0,5	43	1	1	1	1	1	1
31	2	0,5	51	2	2	1	1	1	1
32	2	0,5	29	1	5	2	2	2	2
33	2	0,5	22	1	2	2	2	2	2
34	2	0	18	1	3	2	2	2	2
35	2	0,5	41	1	1	2	2	2	2
36	2	1	67	2	1	1	1	1	1
37	2	0,5	35	5	1	2	2	2	2
38	2	1	50	3	1	1	1	1	1
39	1	1	21	3	1	2	2	2	2
40	2	1	37	3	4	2	2	2	2
41	2	1,5	46	2	1	1	1	1	1
42	2	1	61	2	1	1	1	1	1
43	2	1	65	2	3	1	1	1	2
44	2	0	23	1	4	1	1	1	1
45	2	1	52	2	3	1	1	1	2
1	1	7	39	3	6	2	2	2	2
2	2	3	43	2	6	1	2	1	2
3	2	1	41	2	2	1	1	1	2
4	1	2	35	1	5	2	2	2	2
5	2	4	44	2	5	2	2	2	2
6	1	3	63	2	5	2	2	2	2
7	2	3	38	1	6	1	1	1	2
8	1	3	34	2	5	1	1	1	2
9	1	9	43	2	6	1	2	1	2
10	1	5	41	1	4	2	2	2	2
11	1	4	48	3	4	1	1	1	1
12	2	7	36	2	6	1	1	1	2
13	2	7	45	1	6	1	1	1	2
14	1	2	25	1	5	2	2	2	2
15	2	6	32	3	6	2	2	1	1
16	1	6	32	2	6	2	2	2	2

17	1	6	29	2	6	1	2	1	2
18	1	4	29	1	5	1	2	2	2
19	1	5	40	1	6	2	2	2	2
20	1	4	62	2	4	1	1	1	2
21	1	5	40	5	6	1	1	1	2

quantas vezes autmedica três meses	CONHECIMENTO	LM1	LM2	LM3	LM4	LM5	LM6	LM7
2		2	5	5	5	5	5	5
0		1	1	1	1	1	1	1
15		5	5	5	5	2	3	3
2		4	5	5	4	3	2	5
4		3	3	5	2	1	1	1
6		1	3	5	5	1	5	5
0		2	2	5	2	5	5	5
15		5	5	5	5	5	5	5
10		4	5	5	4	4	2	2
8		1	1	5	1	1	5	4
2		3	3	5	3	2	2	3
0		5	5	5	5	5	4	5
2		3	3	4	3	3	4	4
10		1	1	5	2	1	2	1
20		5	5	5	5	3	1	4
2		1	4	4	2	2	3	3
2		5	5	5	5	5	2	2
90		5	5	5	5	5	1	5
0		3	5	5	4	5	3	5
0		3	5	5	5	4	4	5
0		3	3	5	3	4	3	3
2		3	3	5	3	3	4	5
0		3	3	5	3	2	1	3
2		5	5	5	5	5	2	2
15		2	2	5	2	4	3	3
0		3	4	5	3	5	2	4
0		3	3	5	3	2	4	3
0		5	5	5	5	5	5	4
2		3	3	5	3	5	3	3
0		1	1	2	1	1	3	2
0		1	1	5	2	5	2	2
15		3	3	2	3	2	3	3
0		4	5	5	4	5	4	5
0		3	5	1	1	1	1	1
0		2	2	5	5	3	4	2
5		5	5	5	5	5	5	5
4		3	3	5	5	5	3	3
3		5	5	5	5	5	1	1
2		4	5	5	5	4	1	1
20		1	2	2	1	3	3	4
20		3	3	4	4	2	3	3
20		2	3	5	4	5	2	3
60		3	4	1	3	4	4	1
4		1	1	3	1	1	4	5

5		3	3	5	3	5	5	4
3		3	5	4	2	3	1	2
12		1	5	5	1	1	1	3
4		4	3	1	3	3	1	3
5		5	4	5	5	4	4	1
40		3	4	5	3	1	3	5
20		2	5	5	2	2	5	5
3		3	5	2	2	2	3	4
0		3	4	5	1	1	3	4
5		1	1	1	1	1	3	1
0		3	5	1	1	1	5	3
1		2	5	3	2	2	5	1
10		5	5	5	5	5	1	1
15		2	5	4	4	5	4	3
2		4	5	3	5	3	1	2
10		2	5	5	4	4	1	1
0		1	3	3	1	1	5	1
10		1	5	1	1	1	3	5
4		1	4	3	1	1	1	4
0		1	1	1	1	1	4	3
0		1	1	1	1	1	5	1
3		1	5	1	1	1	3	5
3		1	3	4	1	1	1	1
15		3	5		1	1	3	4
20		3	3	1	4	3	5	1
3		1	5	4	1	1	4	1
60		5	5	5	5	5	4	3
6		1	4	4	2	2	4	5
0		1	3	5	1	1	2	1
1		3	5	3	3	3	5	4
0		5	4	3	4	3	5	4
2		3	5	2	3	3	1	1
20		4	5	1	4	4	3	1
0		1	4	4	1	1	3	2
10		3	3	3	3	3	2	4
20		1	1	1	1	1	1	5
3		5	5	5	5	5	2	3
8		1	5	3	5	5	3	5
40		1	1	1	1	1	1	5
90		1	5	5	1	1	3	2
1		5	5	4	5	5	4	2
20		4	4	3	4	4	1	1
3		4	1	4	5	4	5	5
3		3	5	3	3	3	5	5
0		4	4	4	4	4	1	3
0		1	4	2	1	1	4	1
12		5	3	2	5	1	1	1
3		3	5	2	3	2	4	3
5		5	5	5	5	5	2	3
0		2	4	5	4	4	1	3

4		1	3	5	3	2	2	1
5		3	3	4	2	2	1	1
1		1	1	5	1	5	1	1
3		5	5	5	5	1	2	4
4		1	2	4	1	1	4	4
0		1	1	1	1	1	4	2
4		1	2	4	1	1	3	3
6		1	2	3	1	2	3	3
5		5	5	5	3	1	1	3
10		5	5	5	5	5	1	3
15		1	3	5	3	5	1	5
10		1	5	5	1	3	4	5
0		4	5	5	5	5	5	3
6		3	5	3	1	4	4	4
0		4	4	2	3	3	4	4
0		4	4	4	4	4	3	3
1		2	3	5	3	4	3	3
30		1	3	1	1	1	5	5
12		5	5	5	5	5	5	5
3		4	5	3	5	2	1	3
0		3	5	5	3	1	3	3
3		1	4	5	2	2	3	4
2		1	2	3	3	3	1	1
0		2	4	3	3	2	3	3
20		3	1	3	1	1	4	4
0		5	5	5	1	1	5	5
0		1	1	4	1	1	1	4
20		3	3	1	3	1	3	4
20		1	1	3	3	3	4	4
40		5	1	1	4	1	1	1
20		1	5	2	3	3	4	2
20		3	5	5	3	3	1	3
10		4	4	2	4	4	4	5
5		1	5	3	3	3	1	1
10		2	5	5	5	2	5	4
1		3	5	4	5	2	1	3
1		1	3	1	1	1	2	4
0		1	5	1	1	1	5	3
5		1	5	4	1	1	3	4
10		1	1	5	1	3	4	3
30		1	1	3	1	1	4	3
5		1	2	4	1	3	5	1
5		1	3	5	5	2	3	5
20		1	3	5	1	5	5	1
10		4	5	3	4	4	4	3
4		3	5	1	3	3	4	4
30		5	5	3	3	1	3	4
12		5	4	5	5	4	5	3
6		4	5	1	4	4	1	1
3		3	5	4	3	2	4	4

20		4	4	4	4	4	4	1
0		4	5	1	4	4	2	3
90		1	3	1	1	3	3	1
4		3	5	5	5	5	1	3
1		1	2	3	5	5	5	1
20		1	3	5	1	1	3	1
3		5	5	5	5	3	2	2
0		5	5	5	5	5	1	1
5		3	3	5	1	3	1	1
5		5	5	5	3	5	3	3
1		4	4	5	5	5	3	3
20		1	1	3	1	1	1	1
20		5	5	5	5	5	1	1
4		1	1	1	1	1	5	5
0		5	5	5	5	3	4	5
1		3	4	5	3	3	3	4
10		4	5	1	5	3	3	1
30		3	5	1	4	1	3	3
20		5	5	4	4	5	2	1
6		3	3	3	3	1	5	1
3		5	5	5	5	5	5	2
15		1	5	1	1	1	5	5
9		3	5	1	2	2	5	2
0		5	5	5	5	5	4	1
10		1	1	1	1	1	3	5
6		5	5	3	5	5	3	1
0		5	4	5	5	4	5	1
0		5	4	5	3	3	5	3
0		5	5	5	5	5	5	5
15		1	1	3	1	1	1	3
20		1	1	1	1	1	4	5
5		2	5	5	5	2	3	3
0		1	1	1	1	1	1	1
10		2	5	5	5	5	2	3
0		4	4	2	3	1	4	5
0		5	5	5	5	5	5	1
0		5	5	5	5	4	1	1
10		3	5	3	1	1	1	3
5		1	3	5	1	1	3	3
2		1	2	5	1	1	1	5
7		1	4	5	1	1	5	5
5		3	3	4	2	2	3	3
1		5	5	5	5	5	1	3
5		1	3	5	5	5	1	5
2		5	5	5	5	4	1	3
2		2	5	5	3	5	3	5
8		1	2	3	2	1	4	3
6		5	3	4	5	2	5	4
0		1	5	5	5	1	1	1
2		3	3	5	3	4	4	2

7		1	1	4	1	2	1	3
5		1	2	5	3	1	3	5
0		1	2	5	4	3	1	5
1		3	3	5	3	1	1	3
0		2	1	3	4	2	1	2
2		5	5	4	3	5	1	5
0		3	5	3	3	3	1	1
8		2	3	5	1	1	3	5
15		3	3	5	5	5	5	5
0		5	5	5	4	4	3	3
10		1	5	5	1	1	3	5
5		5	3	4	2	3	1	3
20		5	5	5	5	5	3	1
5		3	4	3	3	3	4	2
3		1	2	2	2	2	3	1
6		1	3	5	1	1	5	2
20		5	5	4	5	5	4	5
10		2	5	2	3	3	4	3
90		5	5	3	5	1	4	1
40		1	5	1	1	1	1	1
9		3	5	5	3	3	4	3
20		2	5	4	2	2	5	1
30		4	4	3	3	2	2	4
5		3	3	3	3	3	5	4
0		1	5	5	1	1	1	1
10		1	5	5	1	5	3	1
6		5	5	2	5	3	5	5
2		5	5	5	5	5	2	1
15		1	1	2	1	2	3	5
16		1	1	1	1	1	3	3
0		5	3	2	3	3	3	3
1		5	5	5	5	5	3	1
15		4	5	4	5	4	5	5
10		2	5	5	1	1	5	1
3		2	3	5	5	5	1	5
3		3	3	5	5	5	5	5
0		1	1	4	2	1	5	1
10		1	3	5	5	5	5	3
20		5	5	5	5	3	5	4
3		5	5	5	5	4	1	4
4		2	5	3	1	3	1	1
12		3	5	5	5	5	3	3
1		2	2	3	2	2	3	3
2		2	4	5	4	4	3	2
36		3	5	5	3	3	2	1
0		3	5	5	5	5	4	4
15		1	3	5	1	3	1	3
9		5	5	4	5	5	2	4
15		5	5	5	5	5	3	3
20		5	5	5	5	5	3	3

15		1	1	3	3	4	3	3
5		3	2	4	3	4	3	3
15		5	5	5	5	5	3	2
3		3	5	4	4	3	1	1
1		5	5	5	5	5	2	1
2		2	2	4	3	2	1	5
10		3	4	4	3	3	2	2
10		2	5	5	2	5	3	5
10		5	5	5	5	5	1	4
30		5	4	5	3	4	1	5
7		3	3	4	3	1	4	2
10		1	1	2	1	1	5	5
10		3	3	4	4	3	2	1
10		5	4	4	4	4	1	2
3		1	1	1	3	5	5	2
0		1	1	1	1	1	1	1
10		1	3	5	1	3	1	5
36		1	2	3	1	2	4	4
9		2	3	4	3	2	4	3
10		5	5	5	5	5	5	1
0		5	5	5	3	5	4	1
60		5	5	4	5	5	3	1
20		5	5	5	5	5	5	1
12		3	5	1	3	4	5	2
0		5	5	5	5	5	4	4
20		1	4	5	3	5	1	5
5		5	5	2	5	5	1	4
5		1	1	2	1	1	1	1
10		3	2	4	5	2	2	2
3		3	4	4	5	5	4	2
40		5	5	5	5	5	1	4
0		1	5	1	1	1	1	1
1		4	5	5	4	4	3	4
20		4	5	1	4	3	5	4
5		1	4	1	1	1	1	1
8		5	3	3	4	4	5	5
15		1	5	5	1	1	5	3
20		3	3	5	3	1	4	3
8		3	3	1	1	1	1	4
5		1	4	4	2	2	5	5
6		3	5	4	4	4	4	4
10		5	5	4	3	3	4	3
10		5	5	5	5	5	3	1
40		1	1	4	1	1	4	1
1		5	4	5	5	3	1	2
4		3	5	3	3	2	3	3
9		1	2	1	5	1	1	5
30		1	3	5	2	1	3	3
29		1	5	5	1	1	5	5
9		5	4	5	5	2	5	4

6		3	1	5	3	2	1	3
0		5	5	5	5	1	1	1
3		5	5	5	5	5	1	3
30		4	5	5	3	1	5	3
3		3	5	5	4	4	2	3
4		3	5	5	3	4	2	5
5		5	4	4	4	4	4	3
10		5	2	3	5	5	2	1
1		3	5	5	3	1	5	5
10		1	4	2	1	3	3	1
0		5	5	5	4	3	1	3
0		5	5	4	1	1	3	5
1		1	5	3	1	3	3	3
0		1	4	5	3	5	4	4
30		3	5	5	2	2	5	2
10		4	5	5	4	4	1	5
10		3	5	5	3	3	2	2
8		1	5	3	1	3	3	3
20		1	5	3	1	1	2	2
40		2	5	1	3	2	4	5
0		4	4	5	5	5	3	3
5		5	5	5	5	5	2	2
10		1	2	4	1	1	5	5
90		1	3	5	1	1	3	3
0		1	5	1	1	1	1	5
15		2	4	3	2	1	1	3
5		2	4	5	3	3	3	3
10		1	1	4	1	1	5	5
90		3	5	5	3	5	1	1
90		5	5	5	5	5	3	3
4		1	5	4	1	1	3	3
30		5	5	5	5	5	5	2
90		1	5	1	1	1	1	5
5		3	3	5	3	5	5	5
0		5	4	5	5	5	4	5
20		3	5	3	1	1	4	3
30		4	5	2	4	1	3	1
40		3	2	4	2	1	3	3
30		3	5	5	5	3	4	1
3		3	5	5	3	2	5	4
2		5	5	5	1	5	1	1
5		2	3	3	2	2	4	3
12		5	4	3	5	5	5	3
6		5	5	4	5	4	3	2
1		1	3	2	1	1	3	4
12		5	4	3	5	5	1	3
0		3	3	5	5	5	3	4
5		2	3	3	3	3	4	3
2		5	5	5	5	5	5	5
3		5	5	3	5	5	4	2

3		2	4	3	5	5	5	1
8		3	1	4	1	1	5	4
2		3	2	2	3	3	1	4
3		1	4	1	1	1	3	1
30		5	5	5	5	5	1	4
6		5	5	5	5	4	3	5
0		3	3	3	3	3	3	2
4		3	4	4	3	3	2	4
2		3	4	5	5	5	3	1
4		1	2	3	1	1	2	5
30		1	5	5	3	1	5	1
40		3	5	1	2	2	1	4
6		5	5	5	5	5	1	4
20		5	5	1	5	5	4	4
30		5	5	4	4	4	5	5
15		5	5	1	5	5	1	4
12		1	5	1	1	1	4	1
4		2	5	4	2	2	4	3
10		1	5	5	1	1	4	5
60		2	3	5	5	5	1	1
30		3	5	4	5	4	5	3
3		3	4	3	5	5	5	5
2		1	1	2	1	5	5	3
3		3	2	1	4	1	3	4
8		1	4	3	1	1	3	3
15		3	1	2	3	3	1	1
20		2	5	1	5	5	1	1
3		5	5	5	5	5	5	4
6		1	2	4	1	1	3	1
0		5	4	5	5	1	1	1
10		5	5	5	5	5	3	1
0		5	5	5	5	5	1	1
10		1	1	1	1	1	5	5
2		1	5	1	1	1	5	5
0		1	2	5	1	1	1	1
5		3	5	5	5	5	3	3
9		1	3	5	3	2	1	5
0		1	1	5	1	2	5	5
2		2	3	5	4	4	2	1
5		3	3	4	4	3	3	1
4		5	5	5	3	3	2	1
20		2	1	5	1	5	3	1
5		1	5	5	2	1	3	3
5		5	5	2	5	5	3	3
10		4	4	4	4	3	3	3
20		5	5	5	5	5	3	3
5		4	4	4	4	4	5	3
2		5	5	3	5	2	5	3
2		1	1	1	1	1	5	4
10		4	3	4	3	3	5	5

10		3	3	5	3	3	3	3
10		1	1	3	1	1	4	5
30		3	4	4	2	2	3	3
20		1	1	2	1	1	4	4
60		5	2	5	5	5	5	5
40		1	5	5	1	1	5	5
5		5	5	5	5	5	1	5
3		1	3	5	1	1	4	5
10		1	1	1	1	1	3	5
5		1	1	1	1	1	1	1
3		3	4	5	5	3	3	4
3		5	5	5	5	5	1	3
4		3	4	5	4	2	2	3
3		5	5	5	5	5	3	3
10		4	4	1	3	1	1	1
2		5	5	5	5	5	1	1
30		3	4	3	4	4	4	4
0		1	1	1	1	1	1	1
0		4	2	4	3	2	1	1
1		1	1	5	1	1	1	3
5		5	5	3	5	5	4	1
20		3	5	4	2	2	1	1
5		1	4	1	1	1	4	3
5		4	5	4	4	4	3	3
5		5	5	5	5	5	3	3
1		5	5	4	5	5	5	3
90		5	5	5	4	4	5	2
1		5	5	4	5	5	3	1
90		5	5	5	5	1	1	1
0		1	1	1	1	1	5	1
0		1	1	4	2	3	4	2
20		4	5	5	4	5	4	3
2		5	5	3	5	5	1	3
20		3	3	5	3	3	1	1
20		5	5	5	5	4	3	3
5		3	3	1	3	1	1	3
7		4	1	4	3	3	4	4
0		5	5	5	5	5	5	5
0		1	1	1	1	1	4	5
2		2	3	5	4	5	4	3
1		5	4	5	3	5	1	5
5		1	1	5	1	1	1	3
15		3	5	5	5	1	5	5
1		3	5	2	1	1	2	5
4		1	1	5	1	1	1	3
6		4	5	4	4	4	1	3
30		1	1	1	1	1	2	3
35		1	1	3	1	3	5	5
10		1	1	1	1	1	1	1
90		1	2	3	3	1	3	3

0		1	1	1	1	1	1	1
8		1	3	5	4	5	5	5
0		1	5	5	1	1	3	5
4		5	5	1	5	3	1	5
4		1	1	1	1	1	1	1
3		3	2	5	3	5	2	5
3		1	1	3	1	1	1	3
4		2	4	5	1	3	5	4
6		2	3	5	2	2	5	1
60		2	2	5	2	5	3	1
0		4	3	2	5	5	5	5
14		1	4	3	1	2	4	2
3		1	5	3	3	3	5	5
60		5	1	1	1	1	1	3
3		4	3	5	1	1	1	5
12		4	3	4	1	1	1	3
5		5	4	4	1	3	1	3
60		1	2	3	1	1	1	1
0		4	5	5	4	3	5	3
0		3	5	5	5	5	1	1
15		2	5	5	2	3	3	3
30		4	2	5	5	5	5	4
5		1	1	5	1	1	3	1
5		1	2	5	1	1	3	5
2		5	5	5	5	5	1	1
40		1	5	5	1	1	3	4
40		2	4	2	1	2	1	1
35		1	1	2	1	1	2	5
0		1	1	1	1	1	1	1
40		1	1	3	1	1	1	5
80		1	1	1	1	1	1	3
0		5	5	5	3	5	1	4
30		5	4	5	5	5	5	5
1		5	4	5	5	5	5	5
12		3	5	3	5	5	5	5
24		3	5	4	1	1	4	1
0		3	1	1	3	3	3	5
20		2	5	3	1	3	1	3
3		5	5	5	5	5	5	4
0		1	5	5	1	1	1	1
3		4	4	4	3	3	4	4
90		4	5	5	3	1	2	4
10		1	3	3	4	2	3	4
0		4	3	5	5	5	5	5
4		2	3	2	3	3	2	3
30		4	4	5	4	4	3	2
40		3	3	5	3	3	4	3
15		1	3	5	2	1	5	1
90		3	4	5	4	3	2	1
0		4	4	5	4	4	3	1

10		3	3	5	3	4	4	1
1		2	5	4	2	3	4	1
0		5	4	5	5	5	1	1
5		5	5	5	5	5	1	1
2		4	2	5	3	3	4	4

LM8	LM9	LM10	LM11	LM12	LM13	LM14	**INTENÇÃO**	I1	I2
5	1	1	1	3	5	5		4	5
1	1	1	1	1	1	1		3	1
4	3	3	1	3	5	5		5	5
4	3	4	2	3	5	4		4	4
5	3	1	5	5	2	2		1	1
5	1	1	1	3	3	3		3	5
5	3	2	3	4	5	3		3	3
5	5	4	4	5	5	5		3	3
5	3	2	3	2	5	3		5	5
5	4	2	2	3	2	1		5	5
5	1	3	2	1	4	5		5	4
5	4	4	4	4	5	5		4	5
3	1	1	1	3	5	5		5	5
1	5	3	1	1	5	5		3	3
5	1	3	3	1	1	5		3	5
3	3	3	3	3	3	3		3	3
5	5	3	3	1	4	4		4	4
5	1	1	1	1	5	5		1	1
3	1	5	3	3	5	1		3	1
1	1	4	4	4	1	2		1	1
3	3	3	5	5	2	2		5	5
5	1	1	3	3	5	5		5	5
3	3	4	3	1	5	3		1	1
2	3	3	3	2	4	5		2	1
4	3	2	2	3	4	3		3	4
4	1	1	2	3	5	3		2	4
3	2	5	5	4	5	5		4	4
5	4	5	5	4	5	5		5	5
5	1	3	5	3	5	5		4	1
4	1	2	2	3	2	3		5	4
5	3	4	4	3	5	3		5	5
4	4	4	4	2	3	3		3	3
5	5	3	3	3	4	3		2	2
1	1	1	1	3	2	2		3	3
2	2	2	3	4	3	3		2	2
5	5	5	5	5	5	1		3	3
3	3	3	3	3	5	3		5	5
5	1	1	5	5	5	5		5	4
3	3	3	1	1	5	5		3	5
4	2	1	3	4	3	1		5	4
4	3	1	1	3	3	2		5	5
4	4	2	2	2	4	4		2	3
5	5	5	3	1	3	1		3	3
4	2	2	3	4	1	1		3	5

3	3	3	3	3	3	3		5	5
4	3	1	1	1	5	5		2	3
5	3	3	3	1	1	1		3	5
4	3	4	4	1	1	3		3	4
4	4	1	1	4	4	3		2	1
5	3	1	1	4	5	2		5	5
5	5	5	5	5	5	3		3	5
4	2	5	5	3	5	3		4	4
4	2	1	1	3	2	2		4	4
4	5	1	1	3	1	1		5	5
1	1	1	1	5	4	3		4	4
3	1	3	3	5	5	5		3	3
5	1	1	1	1	5	4		4	4
4	3	2	2	3	3	4		5	3
5	2	4	4	1	5	3		4	4
5	5	1	1	1	3	3		1	2
5	1	3	3	1	5	3		4	4
3	4	4	4	3	1	1		4	4
4	4	1	1	4	1	1		4	4
4	5	3	3	4	1	1		4	4
1	1	1	1	5	1	1		1	1
3	3	3	3	3	4	3		5	5
5	3	1	1	1	1	1		5	5
5	5	1	1	5	3	2		4	4
5	3	3	3	5	4	2		5	5
4	1	1	1	5	1	1		3	4
4	3	1	1	3	5	4		4	4
5	5	5	5	5	2	3		4	4
3	1	1	1	1	5	5		5	5
3	1	3	3	5	5	3		3	3
4	3	1	1	5	3	3		1	3
4	4	1	1	1	3	3		4	4
4	1	1	1	3	5	2		5	5
4	2	2	1	1	5	4		4	5
4	2	1	1	2	5	4		5	5
5	1	1	1	5	1	1		5	5
5	5	1	3	1	5	5		5	5
4	4	2	2	1	2	2		5	5
5	5	1	1	1	1	1		5	5
5	1	1	1	3	5	5		5	5
4	4	1	1	4	4	5		5	5
5	4	3	3	1	5	2		5	5
5	3	1	1	5	1	5		5	5
3	3	1	1	3	5	3		4	4
2	1	4	4	1	1	3		1	1
4	1	1	1	4	1	1		4	4
3	3	1	1	3	5	3		5	5
5	5	4	4	4	3	3		4	5
4	4	3	3	4	4	3		5	4
5	3	1	5	1	3	5		1	3

3	3	1	1	4	3	5		4	3
5	2	4	1	1	5	4		5	5
5	1	5	5	5	5	5		5	5
4	3	5	5	3	5	5		4	5
3	4	4	4	4	2	2		4	4
4	2	1	1	3	1	1		2	2
3	3	1	2	4	5	4		4	4
3	4	5	5	5	1	4		5	5
5	3	3	1	1	5	5		3	5
5	5	5	3	3	5	5		2	4
5	5	1	1	1	5	5		2	3
5	1	1	3	5	5	5		3	5
5	5	5	3	5	5	4		3	5
3	2	3	3	4	5	3		3	3
2	2	1	2	4	4	4		2	3
5	1	3	3	5	1	3		5	3
5	3	1	1	3	3	4		2	4
5	1	1	1	1	1	4		5	5
5	4	4	4	5	5	4		5	4
5	1	2	5	4	5	5		1	1
4	3	3	5	3	5	4		4	4
5	5	3	3	2	5	3		4	5
3	3	2	3	3	5	5		4	3
3	3	2	3	3	5	4		4	5
5	1	1	1	1	2	1		4	5
4	5	3	3	5	5	5		5	1
4	1	1	3	1	5	2		5	4
5	3	3	1	1	5	3		5	5
4	5	3	3	4	3	4		5	5
5	4	1	1	1	5	5		5	5
4	4	2	2	4	4	4		4	4
3	4	5	5	1	5	4		5	5
4	2	2	2	5	5	4		5	5
5	1	5	4	1	4	4		4	4
5	2	3	3	5	2	2		5	5
5	5	3	4	1	5	4		5	4
4	1	4	4	1	4	4		3	4
3	1	1	1	5	1	1		1	2
4	2	3	3	4	2	3		3	2
5	1	3	1	3	5	1		4	4
5	5	5	3	3	5	5		5	3
5	4	1	1	4	5	5		5	5
5	1	3	3	5	5	5		4	5
5	5	1	1	1	5	3		4	4
4	3	3	3	4	3	3		4	4
5	5	5	5	5	5	4		3	4
4	4	3	3	4	4	5		4	4
2	1	4	4	4	3	4		4	5
5	5	1	5	1	5	4		3	5
4	3	4	4	3	2	2		4	4

4	4	3	3	4	5	3		4	4
3	3	1	4	4	4	4		2	2
2	4	1	1	3	5	5		1	3
3	1	1	1	3	3	5		3	4
5	5	1	1	5	5	5		5	5
5	1	1	1	5	3	4		5	5
3	1	1	1	1	1	1		5	5
5	5	5	5	1	5	5		5	5
1	4	1	1	4	5	5		5	5
4	4	3	3	4	5	5		5	5
3	4	3	5	3	5	5		3	3
4	3	1	1	5	3	1		4	4
5	5	1	2	3	5	5		5	5
5	3	3	1	1	1	1		5	5
5	5	1	1	4	2	4		5	5
4	4	1	4	4	4	5		3	3
3	3	5	5	3	5	4		3	3
4	3	1	1	4	1	4		3	2
5	4	5	5	5	5	3		3	4
4	2	1	1	5	4	1		4	4
5	1	1	1	5	5	5		1	5
5	3	3	3	5	1	4		5	3
5	3	3	3	5	3	4		4	5
1	1	1	1	5	5	4		4	5
3	1	3	3	3	1	1		5	5
5	3	3	5	3	1	3		3	1
1	1	1	1	5	3	5		3	5
5	5	1	1	5	3	3		5	5
5	3	5	5	5	5	5		1	5
5	1	1	1	2	1	1		4	5
5	5	1	1	5	1	1		5	5
3	4	5	5	3	5	5		4	5
1	1	1	1	1	1	1		1	1
4	3	4	5	1	5	5		4	4
5	3	3	4	5	4	3		3	4
4	3	1	1	1	1	1		1	1
3	2	4	5	1	5	5		5	4
4	3	4	4	1	2	1		3	4
3	3	3	1	1	5	5		1	3
3	1	1	1	2	1	1		5	5
4	2	1	1	4	3	1		4	5
3	1	5	5	3	5	3		3	3
3	4	4	5	2	5	5		2	4
5	3	3	3	1	4	2		4	3
5	4	3	3	2	5	3		3	5
3	3	4	1	1	5	4		5	5
4	3	4	3	3	1	2		4	5
4	1	1	1	5	4	5		3	4
5	5	1	1	5	5	5		3	2
4	4	4	4	4	3	5		5	4

3	2	1	1	2	1	1		4	5
5	1	1	1	1	3	4		5	5
1	1	1	1	1	5	3		2	1
4	5	3	1	1	5	5		5	3
1	1	3	3	3	2	2		3	3
1	1	1	1	2	4	4		4	3
1	1	1	1	2	4	3		3	4
3	4	1	3	5	1	1		5	5
3	3	2	3	5	5	5		5	5
2	3	2	3	3	4	3		1	5
5	5	1	1	1	3	3		3	5
5	1	3	5	3	5	5		3	5
4	4	2	1	3	5	3		4	4
4	4	2	3	5	4	5		5	5
4	4	3	3	3	4	4		3	4
4	3	1	1	5	5	5		4	5
5	3	2	2	3	5	5		4	5
5	5	3	3	5	3	4		5	3
5	3	1	1	4	5	3		3	2
5	1	1	1	1	1	3		5	4
5	5	3	3	4	5	5		5	5
5	3	5	5	5	5	3		3	3
3	2	2	2	2	3	3		2	2
4	4	1	1	4	2	3		5	5
1	1	1	1	1	2	3		3	1
4	2	4	3	4	5	5		3	5
5	5	5	5	5	5	3		5	5
1	3	3	1	1	5	5		5	5
3	3	1	1	3	1	1		5	5
3	3	1	1	3	1	1		4	4
1	1	5	5	3	3	5		4	3
1	1	1	3	4	5	4		1	2
5	5	5	5	5	5	5		3	5
1	3	1	3	5	5	5		5	5
5	5	1	1	1	5	5		3	3
3	3	1	3	4	3	2		4	3
4	1	1	3	4	1	1		3	3
5	5	5	5	5	5	5		5	5
5	1	4	5	5	5	5		5	5
1	1	2	1	1	5	5		5	5
5	4	2	4	1	3	3		3	4
3	3	1	4	4	4	3		4	4
5	4	3	2	3	5	4		3	3
4	4	2	2	2	4	2		1	1
3	3	1	1	1	5	4		3	3
3	2	3	1	1	5	5		3	3
5	3	1	1	5	1	1		1	3
4	4	1	1	2	5	5		3	3
3	3	1	2	3	5	4		3	3
3	3	1	3	3	5	4		3	3

1	3	1	1	2	3	4		5	3
2	3	2	2	3	3	2		2	2
4	3	3	3	3	4	4		4	4
1	5	1	1	1	3	3		2	2
5	4	3	5	1	5	4		3	1
5	3	1	1	5	5	1		2	4
4	4	1	3	4	5	4		5	5
5	5	4	4	4	5	5		3	5
5	4	1	3	3	5	5		3	4
5	5	1	1	3	5	4		5	5
5	1	3	3	5	4	4		4	5
4	3	5	5	5	1	1		5	5
2	2	2	1	2	2	2		2	2
2	2	2	2	2	2	2		2	2
1	1	1	1	2	1	2		3	3
1	1	1	1	1	1	1		1	1
5	5	1	1	1	5	5		4	4
5	4	4	4	4	5	5		4	4
5	2	2	4	3	5	4		4	5
5	1	5	5	5	5	3		2	3
5	4	1	1	3	5	5		5	5
5	5	3	3	1	5	4		5	5
5	5	5	5	5	4	5		4	4
3	3	2	2	5	2	2		4	4
5	5	3	3	4	5	5		3	3
5	1	5	5	1	5	5		5	5
3	4	1	1	1	5	4		3	4
5	5	1	1	1	1	1		5	5
4	3	4	4	4	5	3		5	5
5	5	5	3	4	5	2		4	4
4	4	1	1	5	5	5		5	5
1	4	1	1	1	2	2		3	3
4	4	3	3	4	5	5		3	4
5	3	2	2	5	4	4		4	4
5	5	1	1	1	5	5		4	5
5	3	5	5	5	5	3		5	5
5	3	1	4	4	1	2		5	5
5	4	2	4	5	4	5		5	5
4	4	1	1	1	3	3		4	4
5	5	2	2	5	5	4		2	3
5	2	4	4	4	5	4		5	4
4	3	5	5	5	5	4		3	4
5	5	1	1	1	5	5		3	2
5	5	1	1	5	1	5		5	5
4	4	3	5	1	3	4		2	4
4	4	3	3	3	5	5		4	4
4	1	5	5	5	1	5		5	5
5	3	3	5	1	4	5		5	5
5	5	5	5	3	5	5		4	5
3	3	1	1	5	3	5		5	5

4	5	1	1	1	2	4		5	4
2	1	1	1	1	2	2		2	2
3	3	3	5	1	2	3		3	1
5	2	2	5	5	5	4		5	5
3	3	5	4	1	4	4		3	3
5	5	4	4	4	5	5		4	4
5	4	2	3	3	4	4		4	4
4	5	3	5	1	3	4		5	5
5	5	5	5	5	5	5		5	5
4	4	1	3	3	2	3		3	4
3	1	1	1	1	5	3		3	3
5	5	5	5	5	5	1		1	1
3	3	3	3	3	3	3		5	5
4	4	3	4	4	4	4		3	4
5	5	5	5	5	2	2		5	5
5	5	1	3	3	5	4		5	5
3	4	2	2	2	5	3		5	5
3	3	3	3	3	3	3		2	5
2	2	3	3	3	1	1		5	5
5	5	2	5	4	5	5		5	5
4	3	2	2	3	5	5		2	3
3	1	3	3	1	5	5		2	5
5	3	1	1	3	1	1		5	5
5	4	3	3	4	5	5		5	4
5	4	1	1	1	1	3		5	5
2	3	2	2	1	5	4		4	4
4	1	2	1	1	4	3		3	4
1	1	4	4	5	5	3		5	5
5	5	3	3	1	5	3		5	5
4	4	3	2	3	5	5		4	4
3	3	3	3	3	3	2		4	4
5	5	2	2	2	5	5		5	5
1	1	1	1	1	1	1		1	3
3	5	2	2	3	5	3		5	5
5	5	1	1	5	4	3		2	1
5	5	2	2	5	1	2		5	5
5	2	2	1	5	4	4		4	5
5	2	2	1	2	5	4		5	5
3	1	3	1	1	4	5		3	4
5	3	1	1	5	4	5		3	4
5	1	3	3	1	5	5		5	5
3	2	2	3	4	3	2		4	4
5	4	3	3	5	5	5		4	4
5	5	5	5	3	5	3		3	3
3	5	1	1	3	3	2		1	2
5	4	1	1	4	4	3		5	4
3	2	5	5	4	4	4		3	3
4	4	2	2	3	4	3		2	3
5	2	4	4	4	4	4		5	5
5	4	4	4	4	4	3		5	5

5	3	5	5	5	5	3		5	5
4	4	1	1	5	5	5		5	5
4	2	3	3	1	5	5		3	3
5	1	3	3	1	5	5		2	5
5	5	1	1	2	5	5		5	5
5	1	3	3	3	5	5		4	1
4	4	3	4	3	4	3		2	2
2	1	1	1	1	3	4		1	1
3	3	4	3	3	5	5		3	3
4	4	4	3	1	4	4		5	5
5	1	4	4	5	5	5		5	5
5	3	3	3	1	1	2		5	5
5	4	4	4	1	4	3		3	5
5	4	4	4	4	4	3		1	5
5	1	1	1	1	4	3		5	5
5	1	1	1	1	1	1		5	5
5	4	4	4	4	1	2		4	4
4	4	1	1	4	4	4		4	4
5	1	4	4	4	1	1		5	5
5	5	4	5	2	5	5		3	5
5	5	4	5	5	5	5		5	5
3	1	5	5	5	3	1		3	3
5	3	1	1	5	5	5		4	4
5	5	5	5	5	5	5		5	5
5	5	5	5	1	5	5		5	5
5	5	5	5	1	5	5		4	5
5	5	3	1	1	5	5		5	5
5	5	3	1	5	5	5		5	5
5	3	2	4	4	2	5		5	5
1	1	1	5	1	5	5		5	5
5	5	5	5	5	5	5		5	5
2	3	5	5	1	5	5		5	5
5	1	1	5	5	1	1		5	5
5	5	3	3	5	1	3		5	5
3	5	2	2	2	3	5		5	5
4	1	3	5	1	5	5		3	4
5	3	2	1	1	3	4		3	4
1	3	5	1	5	2	1		5	3
4	5	1	1	1	5	5		2	1
1	1	3	4	3	5	5		1	3
5	1	3	3	2	3	5		4	4
5	3	3	3	4	5	5		5	3
3	4	3	5	3	5	5		5	5
4	4	3	3	3	5	5		5	5
2	3	5	5	3	5	5		5	5
4	4	5	5	3	5	5		5	5
3	3	5	5	3	5	5		4	4
3	3	4	4	3	5	5		4	4
3	3	2	2	3	2	2		3	3
4	3	3	3	5	3	4		4	3

4	2	1	1	3	1	2		3	2
5	5	1	1	3	1	1		5	5
5	4	2	1	2	2	2		4	4
4	4	1	1	2	1	1		5	5
5	1	5	1	1	5	5		5	5
5	5	5	5	2	1	2		5	5
5	5	1	3	1	4	5		5	5
5	5	5	5	5	3	3		5	5
5	3	1	1	3	1	1		5	5
4	1	1	1	1	1	1		5	5
4	5	2	1	5	5	4		3	4
3	3	1	1	1	5	5		4	3
5	3	1	1	4	5	3		4	4
1	1	1	1	1	3	5		4	4
3	1	2	3	1	5	5		3	3
5	5	1	1	1	5	4		1	1
5	4	2	3	4	3	2		1	3
1	4	3	1	1	1	3		5	5
4	3	1	4	1	5	5		4	3
4	1	1	1	1	4	4		5	5
5	5	2	2	5	5	5		5	5
5	5	5	5	1	4	3		4	4
3	3	4	4	4	1	3		4	4
5	4	3	3	4	4	4		4	4
5	4	5	5	4	5	5		3	4
5	2	5	5	5	5	5		3	3
5	1	1	1	5	5	5		3	4
5	1	2	2	4	1	3		3	3
5	1	5	5	1	5	5		5	5
5	1	1	1	3	5	1		5	5
4	4	5	5	3	1	1		4	4
2	1	3	5	4	5	5		4	3
5	1	1	5	1	5	5		5	5
5	3	1	1	1	5	5		1	5
2	1	1	1	1	5	5		4	4
3	1	1	3	3	5	5		3	4
3	1	1	1	4	4	4		4	3
1	1	1	2	4	5	5		1	1
1	1	1	1	3	2	2		3	3
5	4	3	3	5	5	5		3	5
5	5	1	1	4	5	5		5	5
5	3	1	1	1	1	1		5	5
5	5	5	5	5	1	3		5	5
3	3	4	1	3	3	4		4	4
5	1	1	1	1	1	1		4	4
5	1	4	4	3	4	4		4	5
5	1	5	5	3	1	1		5	5
5	1	1	5	5	1	1		5	5
5	1	1	1	3	1	1		5	5
4	5	3	1	3	1	1		5	3

1	1	1	1	1	1	1		1	1
4	3	3	1	3	1	1		3	5
5	5	5	5	5	5	5		1	5
1	5	5	1	1	5	5		5	3
5	5	1	1	1	5	1		5	5
3	2	4	5	4	5	5		5	5
5	3	1	1	1	1	1		3	5
5	5	5	5	5	3	3		5	5
5	3	3	5	3	5	5		5	5
5	1	2	4	1	5	5		5	5
5	5	1	1	5	3	3		1	4
3	1	4	1	5	2	2		5	5
3	3	1	5	1	5	5		5	5
5	1	1	1	3	1	1		3	4
5	1	3	5	1	3	3		5	3
4	3	3	3	3	3	3		3	3
4	1	3	5	3	3	3		3	3
5	1	1	1	3	5	5		5	5
5	1	5	5	4	4	3		2	5
5	5	5	5	1	5	5		5	5
5	1	5	5	4	4	3		5	5
4	2	5	5	4	2	5		5	4
5	1	1	1	1	3	3		5	5
5	1	1	1	1	3	3		5	5
5	3	1	1	1	5	5		4	4
5	1	1	1	3	3	3		4	4
5	1	1	3	1	3	3		5	5
5	1	1	1	1	1	1		5	5
1	1	1	1	1	1	1		1	1
3	2	1	1	2	1	1		4	3
5	5	1	1	5	1	1		5	5
5	1	1	1	5	5	5		1	1
3	3	3	3	4	5	5		4	4
3	3	3	3	5	5	5		3	3
5	5	4	4	4	4	3		5	5
5	5	1	1	4	5	4		3	5
5	1	1	1	3	3	2		1	1
3	4	1	1	3	4	4		4	4
5	5	1	1	5	5	5		3	4
1	1	1	1	1	4	4		1	1
4	3	2	4	4	3	3		3	5
5	5	5	3	2	5	4		3	5
3	2	3	3	3	4	4		2	2
5	3	3	5	2	4	4		3	3
4	4	1	2	3	3	3		4	4
3	2	3	3	3	4	4		4	3
5	4	2	3	3	5	5		5	5
3	3	3	2	4	2	1		3	4
4	2	4	5	3	4	4		4	4
4	4	2	4	3	4	3		2	2

4	2	4	4	4	3	3		3	2
4	1	3	3	3	5	5		2	5
1	1	1	1	1	5	5		1	1
5	5	1	1	1	5	4		1	1
5	4	4	4	4	3	3		4	4

I3	I4	I5	I6	I7	I8	I9	I10	**COMPORTAMENTO**	C1
1	1	5	2	2	1	4	1		1
1	1	1	1	1	1	1	1		1
3	3	4	5	1	5	3	1		3
3	3	3	4	1	4	5	5		5
3	5	3	1	1	3	1	1		3
2	3	5	3	4	1	5	4		4
3	3	3	2	3	3	3	3		1
1	3	2	2	2	1	4	1		4
3	3		3	4	3	3	3		5
4	3	5	2	2	5	4	3		5
1	3	3	2	4	5	5	2		5
4	5	4	1	1	4	4	1		1
3	2	3	3	3	2	3	3		3
1	1	1	1	4	1	1	1		4
1	3	3	1	1	5	2	1		4
3	3	3	3	3	3	3	3		3
2	3	3	1	4	2	2	2		3
1	1	1	1	1	1	1	1		1
1	1	1	1	1	1	1	1		3
1	1	1	1	1	1	1	1		1
2	3	3	3	3	1	1	1		4
1	1	2	4	1	1	3	3		3
1	1	1	1	1	2	2	2		1
1	1	1	1	1	1	1	1		2
1	3	4	3	3	3	2	1		5
2	1	3	1	1	1	2	2		3
2	2	3	1	1	3	3	3		1
1	5	5	1	1	1	5	1		3
1	1	1	1	1	5	5	1		1
2	3	3	2	1	2	1	1		4
3	3	4	2	2	4	4	3		4
3	3	3	4	4	5	2	3		4
1	2	1	1	1	3	3	1		1
1	1	3	1	1	1	3	1		3
1	3	1	1	1	1	1	1		5
1	3	3	1	2	5	5	1		3
3	3	5	1	3	5	3	3		5
1	1	1	1	1	1	1	1		5
1	5	1	1	4	3	4	1		3
2	3	4	3	3	3	4	1		4
3	5	5	1	5	4	3	3		5
3	3	4	5	3	3	4	2		4
3	4	4	2	5	5	3	1		2
5	4	2	5	5	4	4	3		5

3	5	5	3	3	4	3	3		5
2	4	3	1	3	2	2	2		3
3	4	5	1	3	3	3	3		4
3	4	4	3	3	3	3	3		3
1	1	4	3	3	1	1	1		3
3	3	5	4	2	1	1	1		5
3	5	5	3	3	3	3	3		4
3	3	4	2	2	4	4	3		4
1	1	1	1	1	1	1	1		1
5	5	5	3	3	5	5	3		3
5	3	5	3	3	1	1	1		1
4	4	2	3	2	2	2	2		3
1	3	5	3	3	4	4	3		4
3	4	4	2	3	3	3	3		4
3	5	3	2	2	5	5	3		3
1	1	5	1	1	5	5	3		4
2	5	5	3	3	5	5	3		1
1	5	5	1	5	3	5	3		3
4	2	4	3	3	4	4	3		3
4	5	5	1	1	5	5	3		1
5	1	4	3	3	1	1	1		1
4	4	4	1	1	5	5	3		3
4	5	5	5	5	5	5	3		3
4	5	5	1	1	5	5	3		4
3	5	5	2	2	2	2	2		5
3	5	5	2	4	1	1	1		3
4	3	1	1	1	1	1	1		4
3	5	5	4	1	3	3	3		3
1	3	3	1	4	4	4	3		1
4	5	4	3	2	2	2	2		3
5	4	4	3	3	4	4	2		1
1	4	4	3	2	3	3	3		3
1	5	5	5	1	1	1	1		4
3	5	2	4	3	4	1	1		1
3	3	3	3	3	3	3	3		4
1	5	5	1	4	5	1	3		5
2	5	5	3	2	2	1	2		4
4	5	3	3	4	4	4	3		5
1	5	5	5	5	1	1	1		4
1	4	5	1	1	4	4	1		5
4	4	4	1	1	1	1	1		4
5	4	5	5	1	1	1	1		5
1	4	3	1	1	5	2	2		4
1	4	4	1	1	4	1	1		3
1	1	4	1	1	4	1	1		1
4	1	1	1	1	4	4	1		1
1	5	1	1	4	1	1	1		4
2	4	5	3	3	1	1	1		2
3	4	3	3	3	2	1	1		4
1	1	5	1	1	1	1	1		1

3	2	3	1	3	3	2	1		3
3	4	3	1	3	4	1	1		5
1	4	5	2	1	5	1	2		4
2	5	5	1	4	5	1	1		5
4	3	2	1	2	3	3	3		3
2	2	1	1	2	2	2	2		1
2	3	2	2	2	4	3	2		4
3	5	5	3	5	5	1	1		5
1	3	3	3	3	3	3	3		5
3	5	5	1	5	5	1	1		5
1	4	5	1	2	5	1	1		4
5	3	3	1	1	5	3	3		3
3	5	5	1	1	4	1	1		1
3	4	4	2	3	2	1	1		5
3	2	2	1	2	1	1	1		2
2	5	1	1	5	1	1	1		2
3	4	5	3	3	3	4	3		2
5	5	5	5	5	5	5	1		5
5	5	5	3	4	4	4	4		5
2	1	1	1	1	4	3	3		1
2	4	3	3	3	4	4	4		1
3	4	5	1	3	1	1	5		5
1	3	2	2	2	1	1	2		4
1	3	4	4	3	3	2	3		3
1	3	5	1	5	3	5	5		5
1	1	1	1	1	1	5	1		1
1	1	3	3	3	1	3	3		1
4	5	5	4	5	5	3	5		5
3	3	4	2		2	2	3		5
1	5	4	3	4	1	1	3		5
4	2	4	4	4	1	1	1		4
1	3	3	3	3	5	5	3		4
3	4	4	3	3	4	4	3		4
1	1	4	1	1	1	1	1		4
3	2	5	2	2	2	2	2		4
1	4	3	1	1	5	3	2		2
3	3	2	2	2	2	1	1		2
5	1	5	2	1	1	1	1		1
1	3	3	1		1	4	4		4
5	1	4	5	1	4	1	4		5
1	5	5	4	5	2	3	3		5
4	2	1	1	1	1	1	1		5
3	2	4	1	5	1	1	5		4
5	5	5	5	5	5	1	1		4
2	4	5	2	4	4	3	2		4
3	4	5	3	3	5	2	3		2
3	4	4	1	4	4	1	1		4
2	2	3	1	3	2	3	2		4
1	4	1	1	1	1	1	1		3
3	4	3	2	1	1	1	1		3

4	4	4	1	4	4	1	3		4
2	3	2	2	1	4	1	1		1
1	3	3	1	1	1	1	1		5
1	5	1	1	1	1	1	1		3
3	3	4	1	1	1	5	5		5
1	5	5	5	5	5	5	5		5
1	1	3	1	1	3	3	1		5
2	1	1	1	1	1	1	1		1
5	5	5	5	5	5	5	5		5
4	5	5	4	3	1	4	1		5
2	3	3	1	2	1	1	1		2
5	5	4	1	3	2	3	1		4
1	1	5	1	4	1	1	1		5
5	3	3	3	3	5	5	1		5
4	4	4	1	1	1	1	1		3
1	3	3	1	3	1	1	1		2
2	3	4	1	1	1	1	1		4
3	1	4	3	3	5	1	3		4
4	4	5	5	5	5	5	5		5
4	4	4	1	4	1	1	1		4
1	1	5	1	3	5	1	3		5
5	5	5	3	4	3	1	1		5
4	3	5	4	4	5	1	3		5
5	4	4	1	1	4	5	2		1
3	4	5	4	4	5	5	4		5
1	5	5	1	1	1	1	1		5
1	5	5	1	1	1	5	1		5
1	5	5	1	1	1	5	1		3
5	5	5	1	1	1	1	1		1
1	3	3	1	1	5	1	3		4
5	5	5	3	4	4	4	4		5
4	5	5	2	4	5	3	5		5
1	1	1	1	1	1	1	1		1
2	3	1	1	3	3	3	2		5
1	3	1	1	1	5	1	1		5
2	1	1	1	1	1	2	1		1
1	5	5	1	4	4	3	3		3
1	5	3	1	1	4	1	3		4
3	3	3	1	1	1	3	1		5
1	5	5	3	3	5	1	1		3
3	3	4	2	4	2	1	3		3
3	3	4	3	3	2	4	4		3
1	4	4	1	2	1	1	1		2
5	3	4	1	1		4	1		3
2	5	5	2	3	1	1	1		4
3	5	5	3	3	4	5	5		3
3	3	3	4	5	4	2	5		5
4	1	5	4	2	3	1	1		3
1	4	4	1	1	3	4	3		4
4	5	4	4	2	4	1	1		3

2	3	5	1	3	2	3	4		3
2	5	5	4	1	1	4	1		3
1	2	3	1	1	4	5	1		3
1	5	5	1	5	5	5	5		2
2	3	3	3	3	4	5	5		1
1	1	5	1	5	5	5	1		2
1	1		1	2	1	1	2		3
4	3	5	3	4	2	3	3		5
2	5	5	3	2	5	5	5		4
1	1	3	1	1	1	1	1		1
4	2	4	1	1	3	1	1		5
3	5	5	1	3	1	1	3		3
3	5	4	2	4	5	2	3		5
4	4	4	2	5	5	1	1		5
4	4	3	3	2	1	1	1		3
5	4	2	3	2	1	1	1		3
3	4	3	3	4	5	5	4		5
3	5	3	3	3	4	1	1		5
3	5	5	3	2	2	1	1		5
4	5	5	2	1	1	1	1		4
5	4	5	2	2	4	3	3		4
5	5	5	5	5	5	5	5		4
2	2	3	1	1	1	3	3		2
4	4	5	3	4	4	1	1		3
1	1	1	1	1	1	1	1		1
2	3	2	1	1	5	4	2		5
5	5	5	3	3	3	2	4		2
1	3	2	1	1	2	2	1		2
3	3	3	3	1	3	3	3		5
3	3	3	2	3	3	3	3		3
1	5	5	1	1	1	1	1		1
1	1	4	1	1	1	1	1		2
3	3	3	3	3	3	3	3		4
1	5	3	5	5	5	5	5		4
1	5	5	1	1	1	1	1		3
3	3	3	2	2	2	4	1		3
3	5	5	1	4	3	4	4		1
1	5	5	3	1	4	5	5		3
5	5	3	1	3	5	5	5		4
5	4	4	1	1	3	3	3		2
1	3	3	4	2	3	2	1		5
3	3	3	2	3	1	1	1		4
2	1	1	1	3	1	1	1		2
1	1	3	1	1	3	1	1		3
2	3	3	2	3	1	1	1		5
1	1	3	1	1	1	1	1		1
3	5	3	1	3	3	1	1		1
3	3	3	2	1	1	2	1		1
1	1	1	1	2	1	1	1		3
1	3	3	1	4	1	1	1		3

2	4	3	3	1	2	5	4		3
1	2	2	1	2	1	2	2		3
1	5	4	3	4	2	2	1		4
1	2	2	2	1	1	2	1		3
1	1	1	2	1	2	1	1		2
2	5	4	2	2	5	5	4		2
3	2	4	3	2	3	4	3		5
1	4	5	1	1	5	1	5		5
1	5	5	2	3	3	3	3		4
1	5	5	5	1	1	1	1		5
2	4	4	2	5	4	1	1		4
3	5	5	5	3	1	1	1		5
2	2	2	2	2	2	2	2		3
2	2	2	2	2	1	1	1		2
2	2	2	1	1	2	2	2		2
1	1	1	1	1	4	4	4		1
1	4	5	4	5	1	1	1		5
4	4	5	4	4	4	1	1		5
2	4	4	3	3	4	2	1		4
5	1	1	1	1	5	1	1		3
4	3	5	3	3	5	5	3		2
2	2	3	1	1	5	5	3		5
4	4	5	1	4	5	5	3		5
4	5	4	2	4	5	5	2		3
3	3	5	2	2	5	5	5		3
1	5	1	1	5	5	5	3		3
1	3	1	1	4	3	3	3		3
1	1	5	1	1	1	1	1		3
3	4	4	2	3	4	3	1		4
1	4	4	2	4	1	4	1		5
1	4	5	1	1	5	1	1		3
1	1	1	1	1	4	4	2		1
2	3	3	4	2	2	2	2		3
2	4	4	2	4	1	3	3		4
1	5	5	5	5	5	1	1		4
5	5	5	5	5	4	1	1		4
4	5	5	4	3	1	1	3		3
4	5	5	1	3	4	4	1		5
3	4	4	4	4	3	3	3		4
4	5	1	3	1	1	1	1		4
3	4	5	4	3	1	1	1		5
4	5	5	3	4	4	1	4		5
1	5	1	1	1	5	1	1		3
4	5	5	1	5	5	1	1		5
1	4	4	3	3	2	3	1		3
3	4	3	2	3	4	4	3		4
1	5	5	1	5	5	1	3		5
4	4	5	5	5	5	1	1		5
5	5	5	1	1	5	1	1		5
3	4	5	1	2	1	1	1		5

1	4	4	2	4	3	2	2		5
1	5	5	1	2	5	1	1		1
3	3	2	1	4	5	1	1		3
5	5	5	2	2	5	1	1		5
2	3	3	2	2	4	2	2		3
2	2	3	3	1	1	3	3		3
2	4	4	3	3	3	3	2		4
1	2	3	3	3	3	2	1		5
5	5	4	2	3	4	1	3		5
3	3	4	1	2	3	1	1		5
1	3	3	1	1	5	1	1		1
1	2	2	1	1	2	1	1		1
2	2	3	3	3	3	3	3		3
3	2	2	2	2	3	2	2		2
1	5	5	1	5	5	5	5		5
1	5	5	5	5	3	1	1		5
3	4	5	5	4	5	1	1		5
3	3	5	5	3	3	3	3		2
3	5	5	3	3	5	3	1		5
1	4	5	4	2	1	1	1		3
1	3	3	1	1	3	1	1		1
1	1	2	1	1	3	5	1		3
3	5	5	5	4	5	5	1		5
4	4	5	2	3	3	1	1		4
1	1	4	4	1	1	1	1		1
2	4	5	3	3	1	1	2		5
3	4	3	3	3	4	4	1		4
4	5	5	1	5	4	4	4		5
1	1	3	3	3	1	1	1		5
3	5	3	1	2	3	2	2		5
4	3	3	3	3	3	3	3		2
2	5	5	4	3	4	4	4		5
1	1	1	1	1	1	1	1		5
2	5	5	3	3	4	3	3		5
1	2	1	1	1	2	2	2		1
5	5	4	3	2	1	1	1		4
5	5	5	3	3	5	1	1		5
5	5	5	3	5	5	1	1		5
4	3	5	1	4	3	5	4		5
5	3	3	2	1	1	1	2		3
1	5	1	1	5	5	1	3		1
4	4	3	4	4	3	4	4		3
3	3	5	1	2	3	2	2		5
2	4	3	2	3	4	3	2		3
5	2	2	1	2	2	2	2		2
2	5	5	1	5	5	1	1		5
4	4	4	1	1	4	5	3		1
3	3	3	3	4	4	1	1		4
5	5	5	1	1	1	1	1		2
5		4	1	1	3	4	3		4

5	5	5	1	5	1	1	1		3
5	5	5	1	5	4	1	1		4
1	2	2	2	2	3	3	3		3
1	5		5	5	5	1	1		2
1	5	5	1	1	4	1	1		5
1	1	5	1	1	5	3	3		4
2	4	2	2	2	1	1	1		1
1	1	4	1	1	1	1	1		3
3	3	3	2	3	1	1	1		3
2	5	5	4	4	4	4	4		4
5	5	5	1	4	4	1	4		5
1	4	4	1	4	4	4	4		4
1	4	4	1	4	4	4	4		4
4	2	4	1	1	4	4	4		4
5	4	4	1	3	4	4	4		5
1	3	4	2	2	4	4	4		5
4	4	4	1	3	1	1	1		4
4	4	4	3	3	3	3	3		3
4	4	4	2	1	1	1	1		3
2	3	5	1	3	1	1	1		5
5	5	5	1	4	3	4	1		5
4	1	1	1	1	5	5	1		5
4	4	5	3	3	1	1	1		2
3	5	5	5	5	5	3	3		3
1	5	5	1	5	5	5	1		5
1	5	5	1	5	5	5	1		5
1	5	5	1	5	5	5	2		5
5	1	5	5	1	5	5	3		5
2	5	5	3	5	4	5	1		5
1	1	1	1	1	4	1	1		1
3	5	5	5	5	5	5	1		5
1	1	2	5	1	3	4	1		1
5	5	2	3	5	5	5	3		5
5	3	2	3	3	5	5	3		2
2	1	5	1	1	1	1	1		2
1	3	3	1	1	1	1	1		4
1	3	4	4	4	3	1	3		4
1	3	5	3	5	5	3	4		1
1	5	4	3	2	2	2	2		3
1	3	3	1	2	3	1	1		3
1	1	2	1	1	2	1	1		3
3	4	4	3	4	4	5	4		4
4	5	2	3	5	5	5	1		2
3	2	5	1	1	5	2	1		2
4	3	4	2	3	5	5	5		4
4	4	5	2	2	3	3	1		4
3	3	4	1	3	3	3	1		4
3	3	4	1	3	4	4	1		3
3	3	3	2	3	5	5	2		2
3	4	3	2	2	3	3	3		4

2	4	3	3	2	2	2	2		4
4	3	5	2	3	5	2	3		5
3	3	3	3	3	2	4	4		5
5	4	5	4	4	4	4	4		5
1	1	5	1	5	1	1	1		5
4	5	5	5	5	5	5	5		5
1	1	3	1	1	5	5	1		5
4	5	5	4	4	5	1	5		5
3	3	5	3	4	5	5	5		4
1	1	5	1	1	2	5	1		4
3	4	3	1	2	4	2	2		3
1	5	5	1	4	1	5	1		5
2	4	4	5	3	3	3	2		5
1	1	3	3	3	5	5	1		5
1	3	3	1	3	4	1	1		5
1	4	1	1	1	4	1	1		3
2	3	2	1	3	2	1	1		4
1	1	1	4	1	1	1	1		1
1	3	4	1	3	5	1	1		1
1	4	4	2	3	1	1	1		3
2	5	5	2	2	1	1	1		5
1	1	4	1	1	4	4	2		3
3	4	3	2	3	1	1	1		4
3	5	4	2	3	1	1	1		2
3	4	4	1	1	3	3	3		3
3	4	3	1	1	5	5	3		2
4	4	4	2	2	4	4	3		4
1	1	4	1	1	1	1	1		3
1	5	5	1	5	1	1	1		5
1	5	5	1	5	5	5	1		3
2	3	4	3	3	4	2	1		2
1	1	3	1	3	1	1	1		5
1	3	3	1	3	5	3	1		3
1	3	3	1	5	5	1	1		5
1	4	4	4	4	5	1	1		5
4	3	2	3	5	2	5	4		3
1	1	1	1	1	3	1	1		3
1	3	1	1	1	1	1	1		1
3	3	3	2	3	4	2	2		1
3	2	1	1	2	3	4	4		3
3	3	5	1	1	1	1	3		3
5	5	3	3	3	2	5	3		5
5	5	5	5	1	5	5	1		5
2	2	3	3	2	1	1	1		1
1	4	4	4	4	4	2	1		3
3	4	4	3	4	4	2	3		5
5	5	5	5	3	5	5	5		5
5	5	5	5	1	5	5	5		5
3	1	3	5	5	3	1	1		3
1	3	4	3	4	3	4	3		5

1	1	1	1	1	4	1	1		1
3	3	2	4	3	2	5	5		4
1	1	1	1	1	1	5	1		1
5	5	5	5	5	5	5	5		4
1	3	5	5	3	3	5	1		3
1	1	5	2	5	1	1	1		4
1	3	5	3	3	1	1	1		3
4	1	5	5	1	1	1	1		3
3	1	3	5	4	3	1	1		5
3	3	5	5	5	1	1	1		5
5	3	5	3	3	1	1	1		1
1	4	3	1	4	1	5	1		5
1	3	5	1	3	1	1	1		3
2	3	5	3	2	5	1	1		4
1	5	3	1	2	3	1	1		2
1	2	3	1	3	1	1	1		3
1	3	3	1	3	3	3	1		3
1	1	5	1	1	3	1	3		4
4	2	2	2	2	3	1	1		5
3	5	3	1	3	5	5	1		1
4	5	4	2	3	3	1	3		4
4	5	5	4	5	5	2	1		4
2	4	5	4	5	5	3	2		3
3	4	5	4	5	5	1	2		3
1	3	4	4	1	3	1	1		2
2	4	3	2	4	5	1	1		4
1	2	4	1	4	5	1	1		4
2	4	2	5	4	5	5	4		4
1	1	1	1	1	4	4	4		1
1	5	3	3	3	5	5	2		5
1	5	5	5	5	5	5	3		4
2	2	1	1	1	1	1	1		1
3	4	5	4	2	1	3	3		5
3	5	5	1	1	5	1	5		3
4	5	3	1	4	5	5	3		4
3	3	3	1	1	1	1	1		4
3	3	1	3	1	1	1	1		2
1	5	4	4	2	4	4	1		4
1	3	4	1	2	1	1	1		3
1	1	1	1	1	1	1	1		1
2	4	2	2	4	5	4	4		3
3	5	4	4	4	1	1	1		5
3	2	3	3	3	2	3	2		3
3	3	3	3	3	5	3	3		5
2	4	3	3	2	3	2	2		3
3	4	4	3	3	2	3	2		5
3	5	5	3	3	3	3	2		5
4	5	5	5	5	3	4	3		5
2	4	5	3	4	3	2	1		5
2	4	4	2	3	3	3	2		1

2	3	3	1	2	2	2	2		3
2	4	5	1	3	1	1	1		2
1	2	1	1	1	1	1	1		1
1	1	4	1	1	4	1	1		3
3	3	4	4	3	2	2	2		4

C2	C3	C4	C5	C6	C7	C8	C9	C10	C11
3	1	1	1	1	1	1	4	3	3
1	1	1	1	1	1	1	1	1	1
3	5	1	3	1	3	3	1	1	1
5	4	2	2	1	1	5	2	3	3
3	1	1	1	1	1	1	3	1	1
5	3	3	1	3	3	3	5	3	3
3	2	1	1	1	1	1	2	1	1
3	2	2	4	1	3	1	1	1	2
5	2	1	2	1	4	3	5	3	3
5	4	1	1	5	5	3	5	1	3
5	2	1	1	1	3	1	3	1	1
3	1	1	1	4	5	4	4	1	2
3	2	1	3	3	2	1	1	3	1
3	1	4	4	1	1	1	3	1	1
3	1	1	1	1	1	1	4	3	1
3	3	2	1	1	2	1	1	1	2
4	2	2	2	5	4	3	2	1	2
1	1	1	1	1	3	1	1	1	1
1	1	1	1	1	3	3	3	3	3
1	1	1	1	1	1	1	1	1	1
5	3	1	1	1	3	3	3	3	3
4	1	1	1	1	4	1	4	1	1
1	1	1	1	1	1	1	1	1	1
1	1	1	1	1	1	1	1	1	1
5	3	1	3	3	3	2	4	1	2
3	2	2	3	1	2	1	3	1	1
3	3	1	3	1	3	2	2	3	2
5	1	1	1	1	5	1	5	1	1
1	1	1	1	1	1	1	1	1	1
4	4	1	1	1	1	2	4	3	3
3	4	1	1	1	1	1	4	2	3
4	4	3	2	4	3	4	4	3	3
1	2	1	1	1	3	2	2	1	1
3	1	1	1	1	1	1	3	3	1
2	2	2	1	1	1	1	1	1	2
3	1	1	3	1	3	1	3	1	1
5	3	2	1	1	3	3	3	3	3
4	1	1	1	1	1	1	1	1	1
5	1	2	3	2	3	2	3	1	1
2	3	1	3	3	2	2	3	2	3
5	4	3	1	1	5	3	5	1	3
4	2	2	1	1	3	1	4	1	2
3	5	1	1	5	3	5	5	3	3
3	3	1	5	2	4	4	4	1	1

5	5	1	1	1	4	5	3	3	3
4	1	1	1	1	3	3	3	2	2
5	3	3	1	1	5	4	5	1	3
3	3	1	3	4	3	3	4	4	4
2	2	1	1	1	4	4	4	1	1
5	3	4	1	1	4	4	4	2	2
5	3	5	1	1	4	5	5	2	3
3	2	1	3	2	4	4	4	1	1
5	3	1	1	1	1	1	3	2	3
5	5	5	1	5	4	4	4	5	5
3	3	4	1	1	3	3	4	1	1
4	1	4	1	2	3	1	1	3	3
4	3	4	1	1	4	4	4	1	1
4	1	2	1	2	1	1	5	4	4
5	2	1	1	4	4	4	4	2	3
1	1	1	1	1	5	5	5	1	1
4	1	1	4	4	4	4	4	1	2
5	5	1	1	1	2	1	1	5	4
4	3	4	2	1	4	4	4	1	2
3	3	1	1	5	5	5	5	4	4
3	3	4	1	1	4	4	4	1	4
4	4	1	4	3	4	4	4	1	1
5	1	2	1	1	5	5	5	5	5
5	3	5	2	1	5	5	5	4	4
4	4	3	1	1	5	5	5	4	4
5	3	1	1	4	4	4	4	2	3
2	2	4	1	2	4	4	4	2	1
5	4	1	1	1	4	4	4	4	4
5	2	3	1	1	1	1	5	1	1
4	4	1	3	3	1	1	5	1	1
5	1	1	1	1	5	5	4	3	4
5	1	2	1	1	5	5	5	1	1
5	4	5	1	1	4	1	5	5	5
4	1	1	1	1	2	1	1	1	1
5	1	4	3	1	3	3	4	1	1
5	3	1	2	1	1	5	5	5	5
5	4	3	1	1	5	5	5	1	1
5	4	1	1	1	3	4	4	1	3
5	4	1	1	1	5	5	5	5	5
5	4	3	1	1	4	4	4	3	3
4	4	1	1	1	2	4	4	1	1
5	3	1	1	1	4	4	4	5	5
5	4	1	1	1	1	3	4	4	1
3	3	3	1	2	4	3	3	1	1
3	1	4	1	1	1	1	4	1	1
4	2	4	1	1	4	1	4	4	4
5	1	1	1	5	5	5	3	3	3
5	3	1	1	1	3	3	3	2	2
3	1	1	2	1	3	2	3	3	4
3	3	1	1	1	3	1	3	3	3

4	3	1	1	3	4	3	4	1	1
3	1	1	1	1	3	1	3	3	3
3	5	1	1	3	5	1	4	1	1
5	1	5	1	1	4	4	5	2	3
3	2	1	1	3	3	3	3	2	1
2	1	1	1	1	2	1	1	1	1
5	3	1	1	1	5	2	5	2	2
5	1	1	1	3	5	3	3	1	3
5	1	1	1	1	3	4	4	1	3
4	1	1	1	1	5	4	3	1	1
5	1	1	3	1	5	5	5	1	1
3	1	1	1	1	3	1	3	1	1
3	1	1	1	1	5	5	5	1	1
4	4	1	1	4	3	3	3	3	4
2	2	1	1	1	2	2	2	1	1
5	4	1	1	2	3	3	1	1	1
2	2	1	1	1	4	3	4	3	3
5	1	1	1	1	5	1	5	5	5
5	4	1	1	2	4	3	5	1	1
2	1	1	3	1	1	1	1	1	1
4	4	3	1	1	1	1	1	1	1
5	1	2	1	1	3	3	5	1	2
3	1	4	4	3	3	3	3	1	3
4	3	2	2	1	4	4	4	1	3
4	5	3	1	1	5	3	5	5	3
5	1	1	1	1	5	1	5	1	3
3	1	3	1	1	1	1	3	1	1
5	4	1	1	1	4	1	5	5	1
4	3	1	1	3	3	1	3	5	5
4	5	1	1	2	3	1	5	1	1
4	3	1	1	1	4	4	4	4	4
4	2	3	1	1	4	4	4	3	3
4	2	3	1	3	4	4	4	2	2
4	1	1	1	1	3	3	3	1	3
4	3	3	1	1	3	3	4	3	3
5	2	1	1	1	4	3	3	1	3
5	3	3	3	2	2	2	1	3	3
1	1	1	1	1	1	1	1	1	1
3	3	2	1	3	3	3	3	3	2
4	1	1	1	1	5	1	4	1	1
4	3	1	1	1	4	3	4	4	4
3	1	1	1	1	5	3	2	1	1
5	5	1	1	1	3	3	4	5	1
5	1	1	1	1	5	4	5	1	1
4	4	3	1	1	3	1	4	3	3
5	1	1	1	1	4	3	4	1	1
4	1	3	1	1	3	4	4	4	3
3	1	1	1	1	3	3	3	3	2
4	2	1	1	1	4	4	1	1	1
4	1	3	1	2	4	3	3	3	3

4	3	4	4	4	4	4	4	4	4
2	1	1	1	1	2	1	1	1	1
4	5	5	5	1	1	1	4	1	1
3	3	1	1	1	3	3	3	1	1
5	1	1	1	1	1	1	4	1	1
5	2	1	1	1	5	1	4	1	1
5	5	1	1	1	1	1	3	1	1
2	1	1	1	1	1	1	1	1	1
5	5	1	3	5	5	5	4	5	5
4	4	1	3	5	4	3	3	1	3
2	1	1	2	1	2	1	2	1	1
4	2	1	3	1	1	1	5	1	2
5	5	1	1	1	4	1	5	1	1
5	5	1	3	1	3	3	3	3	1
3	3	3	1	1	3	4	4	1	1
3	3	3	1	1	4	1	3	1	1
4	3	4	1	1	1	1	1	1	1
5	3	3	3	3	3	4	2	3	2
5	5	5	1	1	4	4	4	1	1
3	3	4	3	1	1	1	4	1	1
5	5	5	1	1	1	1	3	1	1
5	5	1	1	3	5	5	5	3	1
5	1	4	1	1	5	1	4	3	3
3	1	1	3	1	1	1	3	1	1
5	1	1	1	3	3	1	5	5	3
4	3	4	1	1	4	4	4	1	1
5	5	5	1	1	1	3	5	1	1
5	5	4	3	4	5	5	5	1	1
5	5	5	1	1	5	5	5	5	1
5	5	4	1	1	5	1	5	1	1
5	5	4	4	1	5	5	5	1	1
5	5	5	1	1	5	5	5	1	1
1	1	1	1	1	1	1	1	1	1
5	3	2	3	4	4	1	3	1	1
5	4	1	4	1	4	3	5	1	1
1	1	1	1	1	1	1	1	1	1
3	3	1	1	1	5	1	3	3	5
5	4	1	1	1	3	3	3	5	3
5	1	1	1	1	3	3	3	1	2
4	5	1	1	1	2	1	5	3	1
4	3	1	1	1	4	3	4	2	2
3	3	2	1	1	3	3	4	2	3
2	1	1	3	1	4	3	4	1	1
4	1	1	1	1	3	3	3	1	1
5	3	1	1	1	3	3	5	2	2
4	4	3	1	1	3	3	4	1	1
3	3	1	1	1	1	1	3	2	1
5	1	1	1	1	3	2	4	3	3
4	3	1	1	1	3	4	3	3	2
3	3	1	1	1	3	3	3	1	1

3	4	1	1	1	5	4	4	1	1
5	1	1	3	3	4	1	4	1	3
2	1	1	1	1	1	2	2	1	2
5	4	1	1	1	5	1	5	1	4
4	5	1	1	1	3	3	4	3	4
1	4	1	1	1	3	1	5	1	1
2	1	1	1	1	2	1	2	1	1
1	3	1	1	1	3	5	3	1	1
5	4	1	2	1	1	4	4	5	2
3	1	1	1	1	3	1	3	1	2
1	1	1	1	1	4	4	4	1	1
3	3	1	1	1	3	3	3	2	3
5	3	2	2	1	4	5	3	1	1
5	4	3	1	3	5	4	5	1	3
3	3	5	1	3	4	4	4	3	3
4	1	5	1	4	5	1	2	1	3
4	5	2	2	2	3	3	3	2	2
5	2	1	1	1	4	4	4	3	2
5	3	4	1	3	5	3	5	1	1
5	4	1	1	5	5	1	5	4	4
5	5	1	1	1	5	5	5	2	1
5	4	4	1	3	5	5	5	3	4
2	1	1	1	3	1	1	1	1	2
4	4	1	3	2	4	4	4	4	4
4	1	1	1	1	1	1	1	1	1
2	1	1	1	1	2	3	1	1	2
3	5	3	1	1	3	1	3	1	3
2	1	1	1	1	1	3	3	1	1
5	3	1	1	1	3	3	3	1	1
3	3	1	1	1	3	3	3	1	1
2	3	1	1	1	1	1	4	1	1
2	2	1	1	3	1	1	1	1	1
4	4	1	4	1	4	4	4	4	4
5	1	1	1	1	5	5	5	4	5
3	2	1	1	1	3	3	3	2	2
4	2	1	3	3	3	3	3	2	3
3	4	2	1	1	1	4	4	1	1
5	5	1	1	1	4	4	3	1	1
5	3	1	1	1	4	4	4	1	5
3	3	1	1	1	3	3	3	1	1
5	2	5	1	1	4	3	3	2	2
3	1	1	1	1	3	4	3	1	1
3	3	1	1	1	3	2	2	1	1
3	2	1	1	1	1	1	3	2	3
3	1	1	1	1	1	1	3	1	1
1	1	1	1	1	1	1	1	1	1
5	3	3	3	1	3	3	3	1	1
3	1	1	1	3	2	2	3	1	2
3	1	1	1	1	3	1	1	1	1
3	3	1	1	1	3	1	3	1	1

4	5	1	1	2	4	4	4	2	4
3	2	1	1	1	1	1	2	2	1
4	1	1	1	1	3	2	3	3	3
3	1	1	1	1	3	3	3	1	1
3	1	3	1	1	3	1	1	1	1
3	1	1	1	1	4	4	4	2	1
4	3	1	1	1	5	4	4	1	3
5	4	3	1	1	5	5	4	1	1
5	1	1	1	1	4	1	5	1	1
5	1	1	1	1	5	3	4	1	1
4	1	1	1	1	4	1	4	4	2
5	5	1	1	1	5	2	5	5	5
2	1	1	1	1	2	2	2	2	2
2	2	1	1	1	1	1	1	2	2
2	2	2	2	2	1	1	1	1	2
1	1	1	1	1	1	1	1	1	1
5	5	1	2	4	4	1	4	4	4
5	5	1	4	1	4	4	4	4	4
4	1	1	1	1	1	2	3	1	2
5	1	1	1	1	5	1	1	1	1
5	1	1	1	1	4	1	5	1	1
5	4	2	2	4	5	5	4	3	3
5	5	4	5	1	5	4	5	1	1
5	1	1	1	1	4	5	5	2	2
3	2	1	1	1	4	4	5	1	1
5	3	1	1	1	1	1	1	2	2
4	3	4	1	1	3	4	1	3	3
5	1	4	1	1	4	3	3	2	3
5	2	3	1	3	3	3	4	1	2
5	5	4	4	3	4	4	4	2	1
3	1	4	1	1	5	5	5	1	1
1	1	1	2	4	1	4	1	1	1
4	4	3	3	1	4	4	4	1	1
4	4	5	2	1	5	1	5	2	2
5	4	1	1	1	1	4	3	1	1
5	3	1	3	3	4	4	4	1	1
4	3	2	1	3	3	4	5	4	3
5	4	4	1	1	4	4	5	1	3
4	2	2	1	2	4	4	4	3	3
5	1	1	1	4	5	5	5	1	1
5	5	5	1	1	4	5	2	1	2
5	2	1	1	2	5	1	5	1	2
5	5	4	1	1	5	5	5	1	1
5	5	5	1	1	5	3	5	1	1
3	2	4	1	2	4	3	4	4	4
4	2	1	2	1	4	2	3	2	3
5	2	1	1	1	3	5	5	1	1
5	1	1	1	1	3	4	5	4	3
5	5	1	1	1	5	1	5	5	5
3	1	1	1	1	4	1	3	1	1

4	1	1	1	1	2	2	4	3	3
2	1	1	1	1	1	1	1	1	1
4	1	2	1	1	3	3	3	3	3
5	4	1	1	1	5	4	5	4	1
3	2	1	1	1	3	2	3	1	2
3	2	1	1	1	3	1	3	1	1
4	2	3	1	3	3	2	4	1	1
5	4	1	1	1	4	1	4	1	4
5	4	4	1	1	4	3	4	1	1
3	3	3	1	4	2	2	4	4	4
3	1	3	1	1	1	1	1	1	1
2	1	1	1	1	1	2	1	1	1
3	3	1	1	3	3	3	3	2	3
3	2	1	1	3	3	3	2	2	3
5	5	2	2	1	1	1	5	5	1
5	5	5	1	1	1	1	5	5	5
5	3	1	1	1	3	3	5	3	3
3	3	2	3	3	3	2	2	3	2
5	4	3	3	3	2	2	5	5	5
3	2	1	1	3	3	3	4	3	2
3	1	3	1	1	2	2	1	1	1
2	1	1	1	1	1	1	2	1	1
5	5	1	1	1	3	2	5	2	3
4	2	2	4	1	4	1	5	1	2
3	4	1	1	1	4	4	4	1	4
3	3	3	3	1	3	3	4	3	4
4	1	4	1	1	2	1	3	3	3
5	1	1	1	1	1	4	4	1	1
3	1	5	1	1	3	1	5	1	1
5	2	1	1	2	5	4	4	1	1
2	2	1	1	1	3	3	3	2	2
5	4	2	4	4	4	4	4	2	1
1	1	1	1	3	1	1	1	1	1
5	5	1	1	1	4	5	4	1	3
2	1	2	2	1	3	3	2	1	1
5	1	3	1	1	4	4	4	3	4
5	3	1	1	1	4	2	5	4	3
5	4	3	1	2	5	4	5	2	2
5	1	1	3	1	4	1	5	2	1
3	2	1	1	1	3	1	3	1	1
5	1	1	1	1	5	3	1	3	1
3	4	3	1	4	4	3	2	3	3
5	3	1	1	2	5	5	5	2	2
3	3	4	1	1	4	4	4	2	2
3	3	2	1	4	2	2	2	1	2
5	1	1	1	1	5	5	5	1	1
4	1	1	1	1	4	4	4	4	1
4	2	2	1	1	4	4	4	2	1
4	2	3	4	1	4	1	4	3	3
3	1	1	1	1	4	4	4	4	4

5	1	5	4	1	4	4	4	1	3
5	1	4	1	1	4	4	4	1	1
2	2	5	1	1	4	3	2	2	2
5	3	1	1	1	1	1	3	1	4
5	2	1	1	1	5	5	5	1	1
5	1	1	4	1	1	1	4	1	1
2	2	2	1	1	1	1	2	2	1
4	1	1	3	1	1	1	1	1	1
3	3	4	1	1	4	3	3	2	2
5	4	4	1	4	5	4	4	4	4
5	4	3	1	3	5	5	5	4	4
4	1	3	1	1	5	5	5	4	2
4	1	1	1	1	4	4	4	1	1
4	1	4	1	1	5	4	5	1	1
5	5	5	1	1	5	5	5	4	4
5	1	1	1	1	5	5	5	1	1
4	1	4	1	1	4	4	4	1	1
4	1	1	1	1	1	1	4	2	2
5	4	1	1	1	5	1	4	1	1
3	1	1	1	3	5	5	3	5	3
5	3	3	1	1	5	4	5	3	2
5	1	1	1	1	5	1	5	1	1
3	3	1	1	1	2	3	3	1	1
5	5	3	3	3	5	5	5	5	3
5	5	1	3	1	5	5	5	5	3
5	5	1	1	4	5	5	5	5	3
5	5	5	1	1	5	5	5	5	1
5	4	3	2	1	5	5	5	5	1
1	5	1	1	1	5	5	5	3	3
1	1	1	1	2	2	2	1	1	1
5	4	1	2	1	5	5	5	5	1
1	1	1	1	1	2	2	2	1	1
5	4	1	1	1	5	5	2	3	4
3	3	1	1	1	5	5	2	3	1
2	1	3	1	1	5	5	5	5	5
5	1	1	1	1	1	1	1	1	1
5	2	1	1	1	3	3	4	1	4
5	3	1	1	3	1	4	5	1	1
5	1	1	1	1	1	1	3	1	1
3	1	1	1	1	1	1	3	1	1
1	1	1	1	1	1	3	1	1	2
4	3	1	1	1	3	4	5	2	3
3	3	2	1	5	5	2	3	2	2
3	3	1	1	1	4	4	2	1	1
3	3	1	1	1	1	2	5	5	2
4	4	1	1	1	1	3	5	2	2
4	3	2	1	3	3	3	4	2	2
3	3	3	1	2	2	2	4	3	2
4	1	1	1	1	3	3	2	4	4
3	2	3	4	3	2	2	3	3	2

4	3	3	3	3	2	2	2	2	2
5	5	3	3	1	5	2	5	5	5
5	3	1	1	1	3	1	3	3	3
5	5	3	3	1	5	1	5	5	5
5	1	1	1	1	5	2	5	5	5
5	5	1	1	1	5	5	5	5	5
5	5	1	1	1	5	1	5	1	1
5	4	1	4	1	5	3	3	4	5
3	3	2	2	3	5	3	5	3	3
1	3	4	1	1	1	4	3	2	1
4	2	2	2	4	2	3	4	1	4
5	5	1	1	1	5	4	5	1	1
5	2	3	1	1	3	2	3	1	4
5	5	1	3	1	1	3	1	3	5
5	5	1	3	1	1	1	1	3	3
4	1	5	1	1	1	4	4	4	1
3	2	1	1	1	4	2	2	1	1
3	1	1	1	1	3	4	1	1	1
3	2	3	1	1	3	1	3	1	1
5	5	1	1	1	4	4	4	4	4
5	4	1	1	1	5	5	5	1	1
4	1	1	1	1	4	4	4	1	1
4	2	1	1	3	2	2	4	3	1
2	2	1	1	1	4	4	4	1	1
3	1	1	1	1	1	4	4	1	1
3	3	5	1	1	4	4	4	1	3
4	3	1	1	1	4	4	4	3	3
4	1	3	1	1	1	1	1	1	1
5	4	5	1	1	5	5	5	1	1
3	1	1	1	1	1	1	1	1	1
4	1	2	1	1	4	3	3	3	2
5	3	3	1	3	2	1	3	1	3
5	3	1	1	1	3	1	5	3	3
3	5	3	1	3	3	1	1	1	1
5	1	1	1	1	4	1	4	4	3
3	1	3	1	1	3	1	3	1	1
3	1	1	3	1	1	1	3	1	3
1	1	1	1	1	1	1	1	1	1
3	3	3	1	3	3	3	3	3	3
3	1	1	1	1	1	1	2	1	1
5	1	5	1	1	5	5	3	1	1
3	5	1	1	1	5	3	3	1	1
5	1	5	1	1	5	1	1	1	1
2	1	1	1	2	1	1	1	2	1
3	1	1	1	1	3	1	4	1	3
5	3	1	1	1	5	1	5	2	2
5	5	1	1	3	5	1	5	5	5
5	5	1	1	1	5	1	5	5	5
4	1	1	1	1	5	1	4	5	4
3	1	1	1	2	4	3	5	4	3

1	1	1	1	1	1	1	1	1	1
4	3	3	2	1	2	2	1	2	1
5	1	1	1	1	1	1	5	1	1
5	5	1	1	1	5	5	5	1	5
5	5	1	1	1	5	5	3	1	3
1	3	1	1	1	3	3	2	3	2
3	1	1	1	3	3	3	3	1	1
4	1	1	1	2	4	4	4	3	3
5	4	3	1	1	3	3	5	1	1
5	4	5	1	1	3	1	5	1	1
5	3	4	1	1	3	5	5	3	3
2	5	1	1	1	4	1	4	2	2
5	1	1	1	1	5	5	5	1	1
3	1	1	1	1	3	1	1	1	1
3	1	1	1	1	2	1	3	1	1
2	1	1	1	1	3	1	3	1	1
3	3	1	3	1	5	3	3	1	1
5	1	1	1	1	5	1	5	1	1
2	2	1	1	1	4	2	4	1	1
1	1	1	1	1	5	5	3	1	1
4	3	2	1	3	4	2	4	2	1
5	1	1	1	1	4	1	4	2	3
4	1	1	1	1	5	2	5	1	2
4	1	1	2	1	5	1	5	1	1
3	1	1	1	1	3	1	3	1	1
4	1	1	1	1	4	1	4	3	3
4	3	1	1	1	4	1	4	3	3
4	3	1	1	1	4	1	2	4	5
1	1	1	1	1	1	1	1	1	1
4	1	1	1	1	5	1	3	1	5
5	5	1	1	1	5	5	5	5	5
2	1	1	1	1	3	3	5	1	1
5	5	3	3	1	4	2	4	1	3
3	4	2	2	1	3	1	5	1	3
4	3	1	1	3	3	3	5	3	5
3	1	1	1	1	5	3	3	1	1
3	2	1	1	1	3	1	2	2	2
5	4	1	1	3	2	4	4	1	3
4	1	3	3	1	4	4	4	1	1
4	1	1	1	1	1	1	1	1	1
3	2	3	2	2	2	3	2	3	2
4	2	4	1	1	5	3	4	2	2
2	2	1	1	1	2	2	3	2	2
5	5	3	3	1	3	3	3	3	3
4	2	1	2	2	3	1	3	2	2
4	2	3	1	3	2	2	3	3	3
5	5	3	3	3	3	3	5	1	1
5	4	1	5	4	4	3	5	2	4
4	3	2	1	1	3	2	4	2	2
2	1	1	1	1	3	1	3	2	2

3	2	4	1	1	2	2	3	1	1
3	1	1	1	3	3	3	3	1	2
1	1	1	1	1	1	1	1	1	1
4	1	4	4	1	4	4	4	1	1
5	3	5	1	1	4	2	4	1	3

C12	C13	C14	C15	C16	C17	C18	C19	C20	C21
3	3	1	1	1	3	1	5	1	1
1	1	1	1	1	1	1	1	1	1
1	3	3	1	1	1	1	3	1	3
2	2	3	3	1	1	1	1	2	2
1	3	1	1	1	1	1	1	1	1
1	1	5	1	5	1	1	5	1	1
1	1	2	2	1	3	2	4	1	3
2	3	2	1	1	2	2	2	3	3
5	3	5	4	1	1	2	4	4	2
5	4	4	2	1	3	3	5	2	2
2	2	3	1	1	1	2	4	2	3
2	3	5	1	1	4	2	4	2	3
3	3	3	1	3	1	1	3	1	1
1	1	3	1	3	1	1	1	1	1
1	1	2	1	1	1	1	1	1	1
2	2	1	1	1	1	2	3	2	2
2	3	2	4	1	1	1	1	1	1
1	1	1	1	1	1	1	1	1	1
3	1	3	1	1	1	1	3	1	3
1	1	1	1	1	1	1	1	1	1
3	1	3	1	1	1	1	1	1	1
1	1	1	1	5	1	1	1	1	1
1	1	1	1	1	1	1	2	1	1
2	2	2	2	1	1	1	1	1	1
3	2	2	3	1	2	1	2	3	3
4	1	3	1	1	1	2	1	1	1
3	2	2	1	1	4	2	3	3	3
5	1	5	5	1	1	1	3	1	1
1	1	2	1	1	1	1	2	1	1
2	2	2	2	1	2	2	2	2	1
4	1	3	2	1	1	2	3	4	1
3	3	3	4	4	3	4	5	4	4
1	2	1	1	1	1	2	3	1	3
1	3	1	1	1	1	1	3	1	1
1	1	1	3	1	1	1	2	1	1
3	3	3	1	1	3	1	4	1	3
3	3	3	1	1	1	1	1	1	1
1	1	1	1	1	1	1	1	1	1
3	1	3	3	1	1	1	3	3	1
2	3	3	2	1	2	2	3	1	1
5	3	5	3	1	1	1	3	1	1
3	2	3	1	1	2	1	1	2	2
1	3	3	3	5	3	5	3	4	2
2	4	2	1	5	2	4	5	1	1

4	3	4	5	4	4	1	3	3	3
2	1	1	1	1	1	1	1	1	1
4	3	3	4	1	3	1	3	3	3
3	4	1	1	1	3	3	3	1	1
4	1	1	1	1	1	1	1	1	1
4	2	4	2	1	1	1	1	3	2
3	4	3	3	1	1	1	1	3	3
3	2	1	1	1	1	1	1	1	1
1	1	3	1	1	1	1	1	1	1
5	3	3	5	1	5	5	4	1	5
1	5	4	4	1	1	1	1	1	1
3	4	3	2	1	4	1	4	1	3
4	1	1	4	2	1	1	1	1	4
5	3	4	5	5	5	1	5	1	3
3	3	2	2	4	1	1	1	3	3
1	1	1	4	1	4	4	1	1	1
3	1	1	1	1	1	1	1	1	1
4	4	2	1	1	1	1	5	1	5
4	1	1	3	3	1	1	1	1	1
4	4	5	1	1	4	4	4	4	4
1	4	1	4	1	1	1	1	1	1
4	4	4	4	1	3	3	3	3	1
4	4	4	4	1	1	1	4	1	4
4	4	4	4	1	4	4	4	1	4
3	3	4	4	3	3	3	3	3	3
4	4	4	2	1	1	1	2	1	2
3	4	2	4	4	1	1	1	1	1
5	4	2	2	1	3	3	3	5	3
1	1	1	1	1	1	1	1	1	1
3	4	3	4	1	1	1	1	1	1
4	4	4	4	1	4	1	4	4	4
3	1	1	1	3	4	1	4	4	4
4	3	5	5	1	1	1	1	1	1
3	2	2	1	1	1	1	3	1	3
3	3	3	4	1	4	1	3	1	3
5	4	4	4	4	1	1	1	1	1
5	1	3	2	2	1	1	1	3	2
3	4	4	3	1	1	1	3	1	5
5	1	1	1	1	1	1	1	1	1
4	1	3	4	1	4	4	4	4	4
5	5	4	3	1	1	1	1	1	1
5	5	5	5	1	1	1	5	5	1
1	1	1	1	1	1	1	4	1	1
1	2	1	3	3	1	1	4	1	1
1	1	1	1	1	1	1	1	1	1
4	5	4	1	1	1	1	1	1	1
3	3	1	3	1	2	1	1	1	1
4	3	3	2	1	1	1	1	1	1
5	3	3	3	5	4	1	5	1	3
3	1	3	1	1	1	1	1	1	1

3	3	3	2	4	3	2	3	2	4
3	2	3	1	1	1	1	1	1	1
4	3	3	1	5	3	1	5	1	1
5	3	5	1	1	2	1	4	2	4
3	3	2	1	4	2	1	4	1	3
2	2	1	1	1	1	1	1	1	1
3	3	3	2	4	1	1	3	1	1
4	3	3	3	1	2	3	5	1	5
3	3	5	1	1	1	1	3	1	3
1	1	4	1	2	1	1	1	1	1
2	1	5	2	1	1	1	1	1	1
3	3	5	5	1	5	5	5	3	1
1	1	5	1	1	1	1	1	2	1
4	4	3	3	4	1	1	1	1	1
1	3	2	1	1	1	1	1	1	1
3	3	4	5	1	1	1	1	1	3
4	4	4	1	1	2	1	3	1	3
5	5	5	5	5	5	1	5	3	5
4	5	4	4	5	1	1	3	2	3
1	1	4	4	1	1	1	3	1	2
3	3	3	1	3	1	1	1	1	1
3	3	3	2	1	1	1	1	1	3
3	1	2	1	3	1	1	1	3	3
3	1	3	2	1	2	1	3	1	3
2	1	5	1	1	3	1	4	1	3
3	5	5	1	1	5	1	4	3	3
3	3	3	1	1	3	1	3	1	1
5	5	4	1	1	1	1	3	4	1
5	3	3	2	5	3	3	3	3	2
2	3	4	1	1	1	1	1	1	1
4	4	4	4	1	1	1	4	4	4
4	4	3	1	1	4	4	4	4	4
4	4	3	3	1	1	1	4	2	4
4	1	1	1	1	1	1	1	1	1
2	3	4	1	1	1	1	3	1	3
3	2	4	1	3	3	1	4	2	3
3	3	3	2	1	1	1	3	2	1
1	5	1	1	1	1	1	1	1	1
3	3	4	4	1	3	4	5	3	5
1	4	1	1	1	1	1	3	1	1
5	1	3	3	5	3	5	3	5	3
3	3	3	1	1	3	1	1	1	1
1	1	3	1	1	1	1	3	1	1
3	4	5	1	1	1	1	1	1	1
4	2	4	3	1	2	2	4	2	2
4	4	4	1	1	1	1	4	4	4
3	3	3	3	1	1	3	1	1	1
2	2	3	1	1	1	1	1	1	1
3	1	1	2	1	1	1	1	1	1
4	2	3	1	1	1	1	3	1	2

4	4	3	4	3	4	1	4	4	4
2	1	1	1	1	1	1	1	1	1
1	3	5	5	5	4	1	3	1	1
1	1	1	1	1	1	1	1	1	1
5	1	5	5	1	1	5	5	1	5
5	1	1	4	1	1	1	5	1	1
5	1	3	1	1	1	1	1	1	1
1	1	2	1	1	1	1	1	1	1
5	5	5	1	1	1	4	5	1	3
5	5	3	1	1	1	1	3	1	1
1	2	2	1	1	1	1	1	1	1
3	5	3	1	1	1	1	3	1	2
4	1	4	1	1	1	5	1	1	3
5	3	3	1	5	1	1	1	1	1
3	3	3	2	1	1	1	1	3	1
3	1	3	1	1	1	1	1	3	1
1	1	4	4	1	1	1	1	1	1
3	3	4	3	1	3	3	4	3	4
5	4	2	2	1	4	4	4	1	4
1	3	1	4	1	1	1	1	1	1
1	1	1	5	1	1	1	5	1	1
5	5	5	5	1	5	5	5	5	5
3	4	4	4	1	1	4	5	1	3
3	4	1	1	1	4	1	4	1	4
5	3	3	3	1	3	3	5	1	3
2	2	1	4	1	1	1	1	1	3
4	1	1	1	1	3	2	5	4	4
3	1	1	5	5	5	4	5	5	5
5	5	1	1	1	5	5	5	5	5
1	1	5	5	5	5	5	5	5	5
5	5	5	5	5	1	1	4	1	1
5	4	3	3	1	1	1	3	1	1
1	1	1	1	1	1	1	1	1	1
3	2	4	4	4	3	3	3	2	1
1	1	1	1	1	1	1	1	1	1
1	1	1	1	3	1	1	2	1	1
3	1	5	3	1	3	1	5	1	1
5	1	3	5	4	4	1	5	1	1
2	3	3	1	4	3	1	2	1	3
5	1	3	1	1	1	1	1	1	1
4	4	2	1	1	1	1	3	1	2
3	3	3	2	2	3	2	3	1	1
4	4	4	1	1	1	1	1	1	1
3	1	3	1	1	1	1	3	1	1
5	3	3	2	5	1	1	1	1	3
3	3	3	1	1	5	1	5	1	4
4	2	3	1	1	3	1	3	1	1
4	4	4	1	1	1	1	2	1	1
4	4	4	1	5	1	1	3	3	3
3	3	4	1	1	3	3	4	1	1

3	2	3	1	5	1	1	3	2	2
4	1	5	1	1	1	1	4	1	4
1	1	1	1	5	1	1	3	1	4
3	1	5	2	5	1	1	5	1	4
3	3	3	4	3	4	4	5	1	5
1	2	2	2	1	1	1	5	1	1
1	1	2	1	1	1	1	1	1	1
5	5	5	5	4	2	1	5	5	5
5	4	4	1	1	4	5	5	4	5
1	3	5	1	1	1	1	1	2	1
1	4	1	1	1	1	1	1	1	1
3	2	3	1	4	1	1	1	1	1
5	3	4	5	4	4	2	4	1	1
4	4	4	4	4	5	1	1	1	1
4	3	4	3	1	1	1	3	3	3
4	5	3	3	1	1	1	1	1	1
3	3	3	3	4	1	2	5	1	2
4	4	4	3	1	3	3	1	1	1
5	4	1	4	1	4	1	1	3	4
5	4	4	4	1	1	1	5	1	1
5	5	4	4	1	1	1	5	1	2
4	4	4	3	1	1	1	5	1	1
2	2	3	1	1	2	1	2	1	1
4	4	4	4	3	1	1	4	1	4
1	1	1	1	1	1	1	1	1	1
3	2	5	1	5	3	1	5	2	1
3	5	3	1	5	5	3	5	5	5
1	1	4	1	5	1	1	1	1	1
3	3	3	1	5	5	3	3	1	1
1	3	5	1	5	4	1	1	1	1
1	3	3	1	1	3	1	3	1	1
1	1	1	1	1	1	1	1	1	1
4	3	3	1	4	3	4	4	5	5
5	5	1	5	5	5	5	5	5	5
4	4	1	1	1	3	3	3	2	2
3	4	2	2	2	3	3	4	3	3
3	5	4	1	1	1	2	5	5	5
4	1	1	1	1	5	5	5	5	5
4	3	3	3	4	1	1	5	1	1
4	1	5	1	1	4	4	1	1	3
1	3	3	1	1	1	3	3	1	1
3	3	3	3	1	1	1	5	1	1
3	3	3	1	1	1	1	3	1	4
1	1	2	1	1	1	1	3	1	1
1	1	3	2	1	1	3	1	1	1
1	1	1	1	1	1	1	3	3	1
3	1	1	1	1	1	1	1	1	1
1	2	3	2	4	1	1	3	1	2
2	2	3	3	1	1	1	1	1	1
3	3	3	1	1	1	1	1	1	1

4	1	4	2	3	3	5	3	4	3
2	2	2	2	1	2	2	2	2	2
3	1	3	4	1	1	1	1	1	1
1	1	3	1	1	1	1	1	1	1
1	1	1	1	1	3	3	1	1	1
4	2	5	1	1	1	1	5	1	1
5	3	3	3	1	3	3	3	3	3
1	1	1	1	1	1	1	1	1	1
1	1	5	1	1	1	1	3	1	1
4	1	1	2	4	1	1	1	1	1
4	2	4	1	1	2	1	2	1	1
5	5	5	4	1	1	1	1	3	1
2	1	1	1	1	2	2	2	1	2
2	2	2	2	1	1	1	1	1	1
2	2	2	2	2	1	1	2	1	1
1	1	1	1	1	1	1	1	1	1
4	4	4	4	1	3	1	4	4	4
4	4	4	4	4	2	4	4	4	4
4	2	3	3	2	1	1	2	1	2
1	5	2	1	1	4	1	4	1	4
1	1	3	1	1	5	1	5	1	5
3	2	2	4	1	1	1	5	1	1
5	5	3	5	1	1	5	5	3	2
3	4	5	3	1	1	1	1	2	1
3	4	3	1	1	1	1	1	1	1
5	1	1	5	1	5	5	5	5	4
5	1	1	4	3	1	1	3	1	3
2	2	1	3	1	1	1	1	1	1
2	3	3	2	1	1	1	1	1	1
5	3	3	5	5	4	3	4	2	3
3	1	1	1	1	1	1	1	1	1
3	1	3	1	1	1	1	4	1	1
5	4	5	5	1	3	3	3	2	3
4	3	4	4	1	2	1	2	2	2
4	5	4	4	1	1	1	4	1	5
4	1	4	4	1	4	1	4	4	4
5	5	5	5	1	1	1	3	3	1
5	4	4	5	5	1	2	3	2	2
4	4	4	4	1	2	3	4	3	4
2	4	1	1	1	1	1	5	1	1
2	2	4	4	1	1	1	1	1	1
5	4	3	1	1	1	1	4	1	2
5	1	5	1	1	1	1	1	1	1
5	3	3	5	5	1	1	1	1	1
5	1	5	2	4	3	1	5	2	3
4	4	4	1	1	5	1	3	1	3
5	5	5	1	1	5	1	5	1	3
5	3	4	4	1	3	1	3	1	1
5	5	5	5	5	1	1	1	1	1
3	3	3	1	1	1	1	1	1	1

3	1	4	2	1	2	1	4	1	4
2	1	1	1	1	1	1	1	1	1
3	1	3	1	2	1	1	1	1	1
4	4	3	1	1	1	3	1	1	1
3	2	3	2	4	2	2	2	2	2
3	1	1	1	1	1	1	1	1	1
2	2	3	3	2	3	2	3	2	2
4	1	5	1	1	3	1	3	3	1
5	3	3	3	1	1	3	3	3	3
1	1	1	4	5	1	4	1	4	1
1	1	1	1	1	1	1	1	1	1
1	1	1	1	1	1	1	1	1	1
3	3	3	3	3	2	2	2	2	2
2	3	3	3	1	1	4	2	2	3
3	5	3	3	3	5	1	5	5	2
5	1	5	1	1	1	1	5	1	1
5	3	5	3	3	1	1	1	1	1
3	2	3	3	3	3	3	3	3	3
5	5	5	5	5	5	3	3	3	3
4	2	3	3	4	2	2	1	1	1
2	1	2	2	1	1	1	1	1	1
3	1	1	1	1	1	1	1	1	1
5	3	3	3	5	5	3	5	5	5
4	3	3	5	1	2	4	1	5	5
4	1	1	1	1	1	1	1	1	1
3	1	4	3	2	1	1	1	3	3
4	4	4	4	1	3	1	5	1	1
1	4	4	1	1	4	4	4	4	4
3	1	3	4	1	1	1	1	1	1
4	3	4	3	3	2	1	5	2	2
3	3	3	3	1	2	2	2	2	2
4	4	4	4	4	2	4	4	5	5
1	1	1	3	3	1	1	1	1	1
4	3	4	2	4	5	1		3	3
2	1	1	1	1	1	1	1	1	1
4	4	2	4	4	1	1	1	1	1
4	5	3	1	1	1	3	1	1	1
3	5	5	3	1	3	4	3	3	1
5	3	4	3	4	1	1	5	1	3
3	4	3	1	1	1	1	1	1	1
5	1	5	1	1	1	1	3	1	3
3	3	3	2	1	2	3	4	2	2
4	3	4	5	5	3	1	2	1	1
3	4	4	2	5	5	2	4	1	4
4	4	3	1	1	3	1	3	1	3
5	1	3	4	1	1	1	1	1	1
2	2	1	1	1	4	1	4	1	4
2	4	3	3	4	2	4	1	1	1
4	4	3	4	4	1	1	1	1	1
4	5	4	4	4	4	4	4	4	4

5	5	4	2	1	3	3	3	4	2
5	5	5	4	1	1	1	1	1	1
1	1	1	3	1	2	2	2	2	2
4	4	4	3	1	1	1	4	4	1
5	1	1	5	1	4	1	4	1	1
3	2	1	5	1	1	1	1	1	1
1	2	3	2	1	1	1	3	1	1
1	1	1	1	1	1	1	1	1	1
3	3	4	2	1	1	1	1	1	1
5	1	4	4	2	3	3	4	3	3
5	5	4	4	1	1	1	4	4	5
4	1	4	4	1	4	4	4	4	4
4	3	4	4	1	1	1	4	1	1
5	5	3	2	1	1	1	4	1	1
4	4	4	5	1	1	5	4	5	4
5	1	5	4	4	1	1	4	1	4
4	4	4	4	1	4	1	4	3	4
4	4	3	4	1	1	1	1	1	1
4	4	4	4	1	1	1	1	1	1
5	1	3	5	5	1	1	1	1	1
5	5	5	2	1	1	1	3	1	1
5	5	5	1	1	1	3	3	1	1
4	1	2	1	5	1	1	1	1	1
5	1	5	5	5	3	3	3	1	3
5	1	5	5	1	5	5	5	5	5
5	1	5	5	1	3	5	5	5	5
5	5	5	5	1	3	5	5	1	3
5	5	3	5	1	5	5	5	1	1
5	1	5	1	1	1	1	3	1	1
5	1	1	1	1	1	2	2	1	5
5	1	3	2	5	5	5	5	1	5
5	1	1	1	1	1	1	1	2	1
5	5	5	1	1	5	5	5	5	5
5	5	5	1	1	3	3	4	3	2
5	1	1	3	5	1	1	1	1	1
1	1	3	3	1	1	1	1	1	1
4	1	3	1	1	1	1	4	1	4
1	3	3	2	1	5	5	5	5	3
3	1	3	1	1	1	1	1	1	1
3	3	2	1	1	1	1	1	1	1
3	3	1	1	1	1	1	1	1	1
4	3	3	1	1	2	2	5	2	1
3	2	2	2	1	4	2	5	1	3
4	3	1	1	4	1	1	1	1	1
4	3	2	2	1	1	1	5	2	2
5	4	3	1	1	1	1	1	1	1
2	3	3	1	1	2	3	1	1	1
5	3	2	1	1	2	1	4	1	3
5	3	2	1	5	1	1	1	1	4
3	3	2	2	3	4	1	2	3	2

2	3	4	2	1	2	2	2	2	2
5	3	5	3	1	3	4	5	5	3
4	4	4	2	1	4	2	2	1	3
5	5	4	3	4	4	3	4	3	3
5	1	2	1	1	1	1	1	1	1
5	5	5	5	1	5	4	5	5	5
4	1	1	1	1	5	1	1	5	5
5	5	5	5	1	4	1	5	1	4
5	5	5	3	4	3	4	3	4	3
5	1	1	5	5	2	1	2	1	2
3	4	4	2	1	1	1	5	2	3
5	1	5	5	5	1	1	1	3	1
5	2	4	4	1	1	1	4	2	3
1	1	1	1	1	1	1	1	1	1
3	3	1	3	1	1	1	1	3	3
1	1	1	1	1	1	1	4	1	1
2	2	2	2	1	2	2	2	2	2
4	1	1	1	1	1	1	1	1	1
3	1	3	1	1	1	1	1	1	1
4	1	1	1	1	1	1	1	1	1
5	3	4	4	1	1	1	1	1	1
3	1	1	1	4	1	1	1	1	1
4	3	3	3	1	1	1	1	1	1
4	2	1	1	1	1	1	1	1	1
1	1	1	1	1	1	1	1	1	1
4	3	3	3	1	5	1	5	1	5
5	5	4	4	1	1	1	4	1	1
1	1	1	3	1	1	1	1	1	1
5	1	5	5	1	1	1	1	1	1
5	1	1	3	1	1	3	3	3	1
4	3	3	2	1	3	1	3	1	3
3	1	1	3	1	1	3	3	1	1
4	1	3	2	3	1	1	4	1	1
5	1	3	1	1	1	1	1	1	1
3	1	3	1	3	1	1	1	1	1
3	1	3	1	1	3	4	5	1	1
3	1	2	1	1	1	1	3	1	1
1	1	1	1	1	1	1	1	1	1
3	3	2	3	3	1	1	3	1	1
3	3	2	1	1	1	3	3	1	3
5	3	3	3	1	1	1	1	1	1
5	1	3	5	1	1	3	3	3	1
5	5	1	5	1	1	1	1	1	1
1	2	1	1	1	1	1	2	1	1
4	1	3	2	1	1	1	1	1	3
5	4	4	3	1	1	2	1	2	1
5	5	5	4	1	5	5	5	4	4
5	5	3	1	1	5	5	5	1	5
5	1	1	1	1	1	5	1	3	1
4	3	2	3	1	3	3	4	4	3

1	1	1	1	1	1	1	1	1	1
4	3	3	3	4	1	1	3	1	1
1	1	1	1	1	5	1	5	1	5
5	1	5	1	1	5	1	5	1	5
1	1	1	1	4	4	4	4	4	3
5	3	2	3	1	1	2	1	1	1
3	1	3	1	1	1	1	1	1	1
3	3	3	1	1	1	1	1	1	1
5	3	5	5	5	2	3	2	1	3
5	3	5	3	4	1	3	2	1	3
5	3	3	1	1	1	3	1	1	3
4	1	1	1	1	1	1	5	4	1
5	1	5	1	1	1	1	1	5	1
1	1	3	1	1	1	1	3	1	3
3	1	4	1	1	1	1	1	1	1
3	1	3	1	4	1	1	1	1	1
3	1	3	3	1	3	1	5	3	3
1	1	1	1	1	1	1	1	1	1
2	3	3	2	1	4	1	3	3	2
3	3	1	1	1	1	1	5	1	1
4	4	3	3	1	1	5	3	4	3
4	4	4	1	1	1	4	1	1	3
5	2	3	1	1	2	1	4	1	3
5	3	3	1	1	1	1	1	1	1
3	1	1	1	1	1	1	1	1	1
4	2	3	3	1	1	3	1	1	1
5	1	3	4	5	1	4	1	3	1
5	1	3	1	1	5	1	5	1	4
1	1	1	1	1	4	1	4	1	4
5	1	3	4	3	4	3	5	3	3
5	1	4	1	5	1	1	5	1	1
2	1	3	1	5	1	1	1	1	1
5	3	5	1	4	3	2	1	2	2
2	3	3	1	1	2	1	4	4	1
5	4	3	4	1	5	1	3	1	3
3	2	4	3	1	1	1	1	1	1
2	3	3	2	1	1	1	1	2	2
3	3	3	3	1	3	3	1	1	1
3	1	1	1	1	1	1	1	1	1
1	1	1	1	1	1	1	1	1	1
3	2	4	2	1	4	1	4	1	4
3	3	5	4	1	1	1	4	1	1
3	2	1	1	1	1	1	1	1	2
3	3	3	2	5	3	1	2	2	3
3	2	3	2	1	1	1	3	2	2
4	3	4	3	3	2	1	2	2	2
4	3	3	3	1	1	1	5	1	1
5	5	3	2	1	3	1	4	1	3
4	2	4	3	3	1	1	2	2	1
2	3	3	2	2	3	2	3	2	2

2	2	2	1	1	1	1	3	1	1
2	2	3	2	2	1	1	1	1	1
1	1	1	1	1	1	1	1	1	1
1	1	1	1	1	1	1	4	1	1
3	3	4	1	5	2	1	2	1	2

C22	C23	C24	C25
3	4	4	1
1	1	1	1
1	3	3	1
2	1	3	1
1	1	3	1
1	1	1	1
1	1	1	1
1	1	1	1
1	1	1	1
1	1	5	1
1	2	3	1
1	1	2	1
3	1	3	3
1	1	1	1
1	1	1	1
2	2	2	2
4	2	2	1
1	1	1	1
1	1	3	3
1	1	1	1
1	3	3	1
1	1	1	1
1	1	1	1
1	1	1	1
1	1	3	2
3	1	1	1
3	3	3	3
1	1	1	1
1	1	1	1
1	1	1	1
1	1	1	1
3	2	4	5
1	1	1	1
1	1	1	1
1	1	3	1
4	3	1	4
1	1	3	1
1	1	1	1
1	1	3	1
2	1	4	1
1	1	5	1
3	1	2	1
1	5	5	1
1	1	1	1

3	1	3	1
1	1	1	1
1	1	5	1
1	1	3	1
1	1	4	1
1	1	5	1
2	1	5	1
1	1	1	1
1	1	3	1
1	1	5	3
2	2	4	1
3	3	3	1
1	1	4	1
3	3	1	1
1	1	2	2
2	2	4	1
1	1	1	1
1	1	1	1
2	2	3	1
1	1	1	1
1	1	1	1
1	1	3	1
1	1	1	1
1	1	4	1
1	1	1	1
1	1	4	1
1	1	4	3
1	1	5	1
3	4	3	1
1	1	1	1
1	1	1	1
1	1	1	1
1	1	3	3
1	1	1	1
1	1	1	1
1	1	5	1
1	3	1	1
3	3	5	1
1	1	5	1
1	1	4	1
1	3	4	1
1	1	5	1
1	1	5	4
1	1	4	1
1	1	1	1
1	1	1	1
2	3	2	1
1	1	1	1
1	3	3	1
1	1	3	1

1	1	3	3		
5	2	4	1		
1	1	3	1		
1	1	5	1		
1	1	2	1		
1	1	1	1		
1	1	3	1		
1	1	5	1		
1	1	1	1		
5	1	1	1		
1	1	1	1		
1	1	1	1		
1	1	1	1		
1	1	1	1		
1	1	2	1		
1	1	2	1		
1	1	2	1		
1	1	1	1		
1	1	4	1		
1	1	1	1		
4	1	1	1		
2	1	3	1		
1	1	1	5		
1	1	3	1		
1	1	1	1		
1	1	1	1		
1	1	1	1		
3	1	1	1		
1	3	3	5		
1	1	4	1		
1	1	4	4		
1	1	3	1		
2	2	1	1		
1	1	1	1		
3	3	1	1		1
1	1	3	3		
1	1	3	1		
1	1	1	1		
3	3	4	1		
2	2	1	5		
4	5	3	5		
1	1	1	5		
1	3	1	1		
1	1	3	1		
3	3	5	4		
1	1	4	1		
1	1	1	1		
1	1	1	1		
1	1	2	1		
1	1	1	1		

1	1	4	1
1	1	1	1
1	1	1	1
3	3	1	1
1	1	1	1
1	1	4	4
1	1	1	1
1	1	1	1
1	1	3	1
1	5	3	1
1	1	1	1
2	1	3	1
1	1	5	1
1	1	3	1
1	1	3	1
1	1	3	1
1	1	1	1
3	3	4	3
2	5	5	1
1	1	1	1
1	1	5	1
1	1	4	1
2	2	4	1
1	1	1	3
1	1	5	1
1	1	4	1
3	3	5	1
5	5	5	1
1	5	1	1
1	1	5	1
1	1	1	1
1	1	4	1
1	1	1	1
2	1	2	1
1	1	3	3
1	1	1	1
1	1	4	1
1	1	4	3
1	1	1	1
4	1	3	1
1	1	3	1
3	1	4	1
4	1	1	1
3	1	3	1
1	1	3	1
1	1	4	1
3	1	1	1
1	1	1	1
1	1	1	1
1	1	3	1

1	2	1	1
1	1	1	1
1	1	1	1
5	1	5	1
4	3	4	1
1	1	5	1
1	1	1	1
4	3	4	1
5	5	1	1
1	1	1	1
1	1	1	1
1	1	1	1
1	1	1	1
1	1	3	1
4	4	3	1
3	1	1	1
3	3	4	1
1	1	4	1
1	1	4	1
1	1	4	1
1	1	1	1
1	1	5	1
3	3	1	1
4	1	4	4
4	4	1	1
1	1	5	1
1	3	3	5
1	1	1	1
1	3	3	3
1	1	3	3
1	1	3	1
1	1	2	1
2	2	1	1
5	5	5	1
1	1	1	1
3	3	1	1
4	3	1	1
1	1	1	1
3	1	1	1
3	3	2	1
1	1	3	1
1	1	3	1
1	1	2	1
1	1	3	1
2	1	1	1
1	1	2	1
1	1	3	1
1	1	3	1
1	1	3	1
1	1	3	1

3	2	3	5
2	1	1	1
3	1	1	1
2	2	1	1
1	1	2	2
1	1	4	1
2	1	3	4
1	1	1	3
1	1	1	1
3	5	1	1
2	1	1	1
3	1	4	1
2	2	1	1
1	1	1	1
2	1	1	1
1	1	1	1
1	1	1	1
4	2	2	1
2	2	1	1
1	1	2	1
1	1	1	4
1	3	1	2
1	1	5	1
1	1	1	1
1	1	1	1
1	1	1	4
1	1	4	1
3	3	1	1
1	1	4	1
3	3	5	5
1	1	1	1
1	1	1	1
1	1	4	1
1	4	4	4
1	1	5	1
1	1	4	1
3	4	4	1
1	1	1	2
1	1	3	1
3	3	1	1
1	1	4	1
5	1	4	4
1	1	3	3
1	1	4	5
4	1	2	1
1	1	3	1
1	1	1	1
1	1	3	3
1	1	3	5
1	1	1	1

1	3	3	1
1	1	1	1
1	1	1	1
1	1	3	1
1	1	4	3
3	1	1	1
1	1	1	1
1	2	1	1
1	1	3	1
4	4	4	1
1	1	1	1
1	1	1	1
2	2	2	2
1	1	1	1
1	2	5	1
1	1	5	1
1	1	2	1
3	3	3	3
1	1	4	3
1	5	2	1
1	1	1	1
1	1	1	1
1	1	3	5
1	3	4	5
1	1	3	1
3	2	3	3
1	1	2	1
1	1	1	1
1	1	1	1
1	1	1	1
5	2	3	2
1	1	5	5
1	1	3	1
2	1	3	1
1	1	1	1
1	1	4	4
1	1	3	1
2	1	5	1
1	2	4	1
1	1	1	1
1	1	1	1
1	1	3	2
1	1	3	1
1	1	4	1
3	3	1	3
1	1	4	1
1	1	1	1
1	1	4	3
1	1	1	1
1	1	4	1

2	2	1	1
1	1	1	1
1	1	4	1
1	1	5	1
1	1	4	1
1	1	1	1
1	2	3	1
1	1	3	1
1	1	2	1
1	1	3	3
1	1	4	4
1	1	1	1
1	1	1	1
1	1	1	1
1	1	4	1
1	1	1	1
1	1	1	1
1	1	1	4
1	1	4	1
5	1	1	1
2	1	5	3
1	1	1	1
1	2	3	1
3	3	3	5
1	1	1	1
1	1	1	1
1	5	4	1
1	1	5	1
1	1	1	1
1	1	1	1
1	1	1	5
1	1	1	1
1	1	4	1
1	1	3	1
1	1	1	4
1	1	1	1
3	1	1	1
1	1	1	1
1	1	1	1
4	1	1	1
3	1	1	1
3	3	1	1
1	1	1	1
1	1	1	1
1	1	1	1
1	1	3	1
1	1	3	1
1	1	2	1
1	1	3	1
1	1	1	3

2	2	3	1
1	1	2	1
1	1	5	1
2	1	5	4
3	1	1	5
1	1	5	1
1	1	1	1
5	1	5	1
4	1	1	1
1	1	1	5
1	1	2	1
1	5	1	5
3	1	1	2
1	1	3	1
3	1	1	1
1	1	1	1
1	1	1	2
1	1	1	1
1	1	3	1
1	1	4	1
1	1	4	1
1	1	1	3
4	4	3	1
1	1	1	1
1	1	1	1
1	1	3	1
1	1	4	3
1	1	1	1
4	5	1	5
1	1	1	1
1	1	3	1
1	1	3	2
1	1	3	1
3	1	5	1
4	4	4	1
1	1	1	1
1	1	1	1
1	1	1	1
1	1	1	1
1	1	3	1
1	5	3	3
1	1	3	1
1	1	1	1
1	1	1	4
2	1	1	1
1	1	1	4
1	2	5	1
1	1	1	4
1	1	3	1
1	3	1	1

1	1	1	1
1	3	3	1
1	1	1	1
1	1	1	1
1	1	5	1
1	3	3	1
1	1	1	1
4	1	2	1
2	1	5	1
1	1	3	1
3	5	1	1
1	4	4	1
1	1	5	5
1	1	1	1
1	1	3	1
1	1	3	1
1	1	3	5
1	1	1	1
1	1	1	1
1	1	3	1
1	1	3	1
4	1	1	1
1	1	1	1
1	1	1	1
1	1	1	1
1	1	1	2
1	1	1	1
1	1	1	1
1	1	1	1
1	1	1	1
1	1	5	1
1	1	1	3
2	1	2	2
1	1	4	1
1	1	1	1
1	1	2	1
2	2	1	1
1	1	3	1
1	1	4	1
2	2	1	1
1	1	1	1
4	1	1	1
2	2	1	1
1	1	5	3
1	1	2	1
3	1	3	2
1	1	4	1
3	1	4	1
1	1	5	5
2	1	1	1

1	1	1	1	
1	2	1	1	
1	1	1	1	
1	1	1	1	
1	1	3	1	
