# Supplementary material for: Evidence of validity of the Risk Self-Medication Questionnaire focused on Health Literacy
Source: Rev Bras Enferm. 2024 Jul 29;77(3):e20230386. doi: 10.1590/0034-7167-2023-0386 (PMC11290745; doi:10.1590/0034-7167-2023-0386)
Supplement: Translation

# Translation: Evidências de validade do Questionário da Automedicação de Risco focado no Letramento em Saúde

**DOI:** 10.1590/0034-7167-2023-0386pt

**Authors:** Márcio Adriano Fernandes Barreto, Francisca Diana da Silva Negreiros, Virna Ribeiro Feitosa Cestari, Helena Alves de Carvalho Sampaio, Thereza Maria Magalhães Moreira

**Affiliations:** I Universidade do Estado do Rio Grande do Norte. Pau dos Ferros, Rio Grande do Norte, Brasil; II Universidade Federal do Ceará, Hospital Universitário Walter Cantídio. Fortaleza, Ceará, Brasil; III Universidade Estadual do Ceará. Fortaleza, Ceará, Brasil

**Keywords:** Automedicação, Risco, Letramento em Saúde, Estudo de Validação, Psicometria.

## Abstract

**Objetivos:**

analisar as evidências de validade da estrutura interna do Questionário da Automedicação de Risco Focado no Letramento em Saúde.

**Métodos:**

estudo psicométrico, com 499 adultos. Avaliou-se a estrutura interna com análise fatorial exploratória e confirmatória para comprovar o ajuste. A consistência interna foi medida pela fidedignidade composta e pelo coeficiente ômega de McDonald (ω).

**Resultados:**

os parâmetros revelaram modelo de 35 itens distribuídos em quatro fatores, explicando 56% da variância total, com cargas fatoriais variando de 0,31 a 0,85 e comunalidades adequadas. Os indicadores precisão (0,79<ORION<0,98), representatividade (0,89<FDI<0,99), sensibilidade (1,92<SR<7,07), esperança do fator (88,3%<EPTD<97,9%), replicabilidade (0,82<H-Latente<H-observado<0,87) e confiabilidade (ω=0,87) foram adequados. A fidedignidade composta variou de 0,840 a 0,910. Além disso, alcançou-se bom ajuste do modelo (TLI = 0,99; CFI = 0,99; GFI = 0,95; RMSEA = 0,02; e RMSR = 0,05).

**Conclusões:**

obteve-se instrumento com boas evidências de validade de estrutura para mensuração da automedicação.

## INTRODUÇÃO

A automedicação de risco (AR) se ancora na definição da automedicação da Organização Mundial da Saúde (OMS)^(1)^, incorporando os elementos potenciais de risco à saúde humana, constituindo-se por elementos envolvidos na automedicação inadequada^(2)^. Desse modo, predizer AR perpassa por reconhecer elementos constitutivos relevantes, como o letramento em saúde (letramento em medicamentos)^(3-4)^, o comportamento e a intenção do comportamento^(5)^. Nessa perspectiva, o letramento é voltado às habilidades de busca, análise e aplicação de informações em contextos de uso de medicamentos^(6)^, impactando a decisão e a prática da automedicação.

O contexto da prática da automedicação inadequada incorre em desperdício econômico, dano ao serviço de saúde e desenvolvimento de bactérias resistentes a antimicrobianos^(7)^. Desse modo, tem-se os elementos potenciais de risco à automedicação que, segundo a OMS, estão relacionados ao autodiagnóstico errado, à escolha incorreta da terapia, ao não reconhecimento dos efeitos adversos, às interações medicamentosas, à contraindicação, ao armazenamento inadequado, ao erro na dose^(2)^, além de não ler os rótulos^(8-9)^ e as bulas dos medicamentos antes de usar^(9)^. Estudos demonstram que pessoas com baixo letramento em medicamentos são mais propensas a praticar automedicação inadequada ou de risco^(4,9)^.

Destarte, medir os riscos que a automedicação pode provocar à população constitui tarefa relevante à saúde pública^(10)^. Nesse cerne, pesquisa realizada pelas Nações Unidas (ONU) estima que, até o ano de 2050, esses riscos podem incorrer no óbito anual de dez milhões de pessoas^(11)^. O Brasil é responsável por 35% das vendas totais de medicamentos no país^(12)^, sendo o quinto país que mais consome medicamentos no mundo^(13)^. Soma-se a isso o aumento da automedicação nos últimos oito anos (de 72% para 81% em 2022)^(14)^. Essa prática tem parcela relevante nas intoxicações por medicamentos, com índice de letalidade de 0,25%^(15)^.

No que concerne ao processo de evidência de validade, tem-se a validade de conteúdo como etapa importante, que avalia a concordância entre itens e construto^(16)^, obtendo parâmetro válido e reprodutível. Entretanto, mesmo que o instrumento tenha apresentado evidências satisfatórias nessa etapa, torna-se necessário analisar as evidências de validade da estrutura interna.

Ainda que a literatura evidencie relação entre a automedicação e o letramento em saúde, até o momento, pouco são os esforços para construir e validar um instrumento que mensure a automedicação na perspectiva do letramento em saúde. Desse modo, a busca de evidências de validade de um instrumento que permita mensurar aspectos relativos à AR se torna oportuna para fortalecer mecanismos de articulação, monitoramento e avaliação direcionada à promoção do uso racional de medicamentos^(17)^, além de impulsionar políticas que possam contribuir de forma direta e direcionada para melhoria assistencial dos serviços de saúde, com vistas a minimizar danos causados pela prática inadequada da automedicação.

## OBJETIVOS

Analisar as evidências de validade da estrutura interna do Questionário da Automedicação de Risco Focado no Letramento em Saúde (QAR-LS).

## MÉTODOS

### Aspectos éticos

O projeto deste estudo foi aprovado pelo Comitê de Ética em Pesquisa da Universidade Estadual do Ceará em 2022, de acordo com as recomendações da Resolução n^o^. 466/2012 do Conselho Nacional de Saúde. Os participantes assinaram o Termo de Consentimento Livre e Esclarecido (TCLE).

### Desenho, período e local do estudo

Trata-se de estudo psicométrico, focado na análise de evidências da estrutura interna^(16)^ do QAR-LS, desenvolvido com a população cadastrada em instituições primárias de saúde (n=12), que buscaram atendimento na Atenção Básica da cidade de Pau dos Ferros, Rio Grande do Norte (RN), Brasil.

A pesquisa foi realizada em todas (n=12) as unidades de saúde de Atenção Primária à Saúde, abrangendo as localidades rurais e urbanas. As unidades são dos diversos territórios do município, apresentando estratos socioeconômicos diversos, que vão desde situações de vulnerabilidade a condições mais favoráveis, englobando pessoas com os diversos níveis de letramento em saúde.

### População, amostra; critérios de inclusão e exclusão

Participaram do estudo adultos e idosos cadastrados nas instituições primárias de saúde, selecionados por conveniência. Incluíram-se pessoas acompanhadas nas instituições de saúde com idade > 18 anos e que buscaram a instituição para prestação de serviços da Atenção Básica. Excluíram-se pessoas sem condições cognitivas de responder.

O tamanho amostral deste estudo psicométrico tomou como base o número de itens, na proporção de mínimo de dez participantes para cada item^(18)^. Logo, o QAR-LS com 49 itens teve a amostra estimada para 490 participantes. Ao considerar a extensão do banco de itens geral, e com a pretensão de preservar a heterogeneidade e conseguir respondentes que cobrisse todo o construto, convidaram-se 536 pessoas (490 + 10%).

Todavia, 36 sujeitos não aceitaram participar e houve perda de um participante por incompletude nas respostas aos itens. Dessa forma, resultou em 499 participantes, que garantiram média de 10,2 observações para cada item do instrumento. Desse modo, o quantitativo de participantes foi adequado com base nas recomendações da literatura^(18)^.

### Protocolo do estudo

Os dados deste estudo são originários de tese de doutorado. Em etapa anterior, ocorreu a construção e validação de conteúdo do Questionário de Automedicação de Risco (QAR)^(19)^. Para tanto, adotou-se a definição de automedicação da OMS, incorporando os elementos potenciais de risco da automedicação^(1-2)^. Para conhecer os elementos constitutivos da automedicação, realizou-se revisão integrativa que avaliou os instrumentos com evidência de validade que medem automedicação no Brasil, chegando aos elementos: letramento em medicamentos; intenção do comportamento; e comportamento.

Em seguida, desenvolveu-se o banco de itens a partir de duas revisões de escopo, envolvendo os elementos do letramento em saúde, mais especificamente o letramento em medicamentos^(6,20)^ e a Teoria do Comportamento Planejado, para auxiliar nos componentes intenção do comportamento e comportamento propriamente dito da automedicação, que incorre em riscos à saúde humana. O banco resultou em 136 itens, que passaram pelo processo de validade de conteúdo, em duas rodadas com juízes de várias regões do Brasil, que apresentavam *expertise* na área de uso de medicamentos e de validação de instrumentos. Após esse processo, o QAR foi considerado validado e confiável, com 49 itens e três elementos constitutivos (letramento em medicamentos, intenção do comportamento e comportamento)^(19)^.

Nessa fase, avaliou-se a estrutura interna do instrumento, aplicando o instrumento com amostra da população (adultos e idosos). Participaram da coleta o autor principal e cinco acadêmicos de enfermagem, previamente treinados pelo autor principal. Utilizaram-se, na coleta de dados, um questionário com dados sociodemográficos (sexo, idade, renda, escolaridade, profissão), um questionário com variáveis clínicas (problema de saúde, uso de medicamentos de uso contínuo e quantidade de uso de medicamentos sem prescrição médica nos últimos três meses) e o banco composto por 49 itens, com padrão de respostas por meio de escala do tipo Likert de cinco pontos, com variação de 1 a 5 (de nunca a sempre).

Os dados foram coletados de fevereiro a maio de 2023, em entrevistas individuais fechadas, realizadas nas unidades primárias de saúde. Os pacientes eram contactados e a eles eram explicados os objetivos e a relevância da pesquisa. Os que aceitaram participar do estudo assinaram o TCLE em duas vias. Os pacientes foram, então, encaminhados a uma sala reservada para garantia da privacidade.

### Análise dos resultados e estatística

Na análise dos dados sociodemográficos e clínicos dos participantes da pesquisa, calcularam-se as frequências absoluta e relativa, para as variáveis categóricas, e medidas de tendência central e dispersão, para as variáveis numéricas, dependendo da normalidade, verificada pelo teste Kolmogorov-Smirnov.

A estrutura interna do QAR-LS foi validada pela Análise Fatorial Exploratória (AFE) e Análise Fatorial Confirmatória (AFC). A adequação dos dados ocorreu pelos testes Kaiser-Meyer-Olkin (KMO) e de esfericidade de Bartlett (BTS), com valores esperados para KMO>0,60 e de BTS de p<0,05^(21)^. Após, verificou-se a dimensionalidade do instrumento pela análise paralela, via técnica *Parallel Analysis Optimal Implementation* ^(22)^, com associação de *bootstrap* extrapolado para 1.000 casos^(23)^.

A extração dos fatores foi executada pelo método *Robust Unweighted Least Squares* (ULS), com correlação policórica^(24)^ e rotação *Robust Promin* ^(25)^. Ainda, utilizaram-se as técnicas *Unidimensional Congruence* (único) > 0,95), *Explained Common Variance* (ECV) > 0,85) e *Mean of Item Residual Absolute Loadings* (MIREAL) < 0,30) para testagem da dimensionalidade dos fatores^(26)^.

Os fatores foram avaliados quanto à precisão (*Overall Reliability of fully Informative prior Oblique N-EAP scores* (ORION) > 0,70), à representatividade do traço latente e efetividade da estimativa dos fatores (*Factor Determinacy Index* (FDI) > 0,80^(27)^, à sensibilidade (*Sensitivity Ratio* (SR) > 2,0), à porcentagem esperada do fator (*Expected Percentage of True Differences* (EPTD) > 90%) e à replicabilidade (*Generalized G-H Index* > 0,80)^(26)^.

Mantiveram-se itens com correlação acima de 0,2 com outros dois itens, com comunalidades (h^2^) e cargas fatoriais acima de 0,40 e 0,30, respectivamente. Excluíram-se os itens com *Heywood cases* e dupla saturação^(18)^. Observou-se, ainda, a convergência da matriz policórica, curtose e assimetria.

Para a AFC, avaliaram-se os índices de ajuste ao modelo: razão de qui-quadrado pelos graus de liberdade (X2/gl≤5,0); *Tucker-Lewis Index* (TLI > 0,90); *Comparative Fit Index* (CFI > 0,94); *Goodness of Fit Index* (GFI) ≥ 0,95; *Root Mean Square Error Approximation* (RMSEA) ≤ 0,07; *Root Mean Square of Residuals* (RMSR < 0,08); *Adjusted Goodness of Fit Index* (AGFI) ≥ 0,93; e qui-quadrado < 0,05^(28)^.

Por fim, a confiabilidade foi calculada pelo Coeficiente Ômega de McDonald () e fidedignidade composta (FC). Os valores de referência adotados para e FC foram: <0,6, baixa; entre 0,6 e 0,7, moderada; e entre 0,7 e 0,9, alta^(18)^.

Para realização das análises estatísticas, empregaram-se os *softwares Statistical Package for the Social Sciences* (IBM SPSS), versão 23, Factor (versão 11.05.01) e R (versão 3.6.2). O cálculo da FC ocorreu pela *Composite Reliability Calculator*, via site www.thestatisticalmind.com.

## RESULTADOS

Entre os 499 participantes da pesquisa, houve predomínio de pessoas do sexo feminino (367; 73,5%), com idades de 18 a 89 anos (Md[p25-p75]=41[30,0-52,0]), com companheiro(a) (256; 51,3%), de escolaridade variada, como ensino fundamental incompleto (70; 14%) e completo (47; 9,4%), ensino médio incompleto (27; 5,4%) e completo (203; 40,7%), ensino superior (37; 7,4%) e pós-graduação (18; 3,6%). A renda autorreferida relatada pelos participantes variou de zero a 15 salários mínimos, com prevalência de um salário (210; 42,1%).

Quanto aos dados clínicos e comportamentais, 238 (47,7%) possuíam algum problema de saúde, sendo que 213 (42,7%) tinham doença crônica e 237 (47,5%) faziam uso de medicamento contínuo. A polifarmácia foi referida por 103 (20,6%) dos participantes. A quantidade máxima de dias que praticou a automedicação nos últimos três meses foi de 90, com mediana de cinco dias (Md[p25-p75]=5[2,0-15,0]).

O modelo 1, com todos os itens (49) do QAR-LS, apresentou matriz negativa e percentual de destruição inadequado (46,3%), KMO inaceitável, pois dez não saturaram e quatro tiveram carga em mais de um fator. Dessa forma, excluíram-se 14 itens (7, 10, 11, 22, 28, 29, 30, 34, 35, 40, 44, 46, 47 e 49) e realizou-se nova análise.

O modelo 2 revelou matriz positiva e boa adequação (KMO=0,83[0,72-0,89]; BTS=5589,5, gl=595, p<0,001). Por meio do *scree plot* (Figura 1) da análise paralela, a exploração dos itens demonstrou estrutura com quatro fatores dominantes (linha verticalizada), na qual os dois primeiros fatores são responsáveis pela maior variância explicada dos dados (F1=28,3%; F2=13,4%; F3=7,9%; e F4=6,4%; variância total = 56%). Os índices UniCo (0,79 [IC95% = 0,78-0,82]), ECV (0,67 [IC95% = 0,65-0,72]) e MIREAL (0,25 [IC95% = 0,22-0,28]) atestaram a multidimensionalidade do modelo.

**Figura 1:** *Scree plot* dos itens do instrumento Questionário da Automedicação de Risco Focado no Letramento em Saúde (QAR-LS), obtido pela análise paralela, Fortaleza, Ceará, Brasil, 2023

A Tabela 1 detalha os fatores e resultados descritivos do QAR-LS. O primeiro fator (04 itens - 6, 12, 17 e 37) englobou aspectos relacionados à norma subjetiva, influência de outras pessoas, como familiares, vizinhos e amigos, na tomada de decisão, na opinião, na recomendação e no motivo da prática de automedicação. O segundo fator (06 itens - 23, 24, 41, 42, 43 e 45) envolve itens referentes à possibilidade e execução da prática de alterar o percurso do tratamento prescrito por profissional de saúde. O terceiro (07 itens - 1, 2, 3, 4, 5, 13 e 14) foi composto por itens concernentes às questões do letramento em medicamentos, considerando as habilidades da pessoa usar o medicamento corretamente. Os itens são inversamente proporcionais ao construto AR. O quarto fator (18 itens - 8, 9, 15, 16, 18, 19, 20 ,21, 25, 26, 27, 31, 32, 33, 36, 38, 39 e 48) ficou com os itens relativos à atitude e ao comportamento da AR. Também reportaram-se os índices de FC.

**Tabela 1:** Cargas fatoriais, comunalidades, curtoses e confiabilidade do modelo final com quatro fatores, Fortaleza, Ceará, Brasil, 2023

Itens	Cargas fatoriais				K	h^2^
	F1	F2	F3	F4		
1			0,759		-1,482	0,599
2			0,638		-0,878	0,430
3			0,567		-0,757	0,342
4			0,850		-1,529	0,712
5			0,806		-1,557	0,648
6	0,739				-1,380	0,503
8				0,529	0,324	0,265
9				0,317	-1,335	0,277
12	0,728				-1,379	0,469
13			0,720		-0,999	0,533
14			0,676		-1,035	0,492
15				0,746	-0,436	0,507
16				0,784	0,271	0,516
17	0,784				-1,080	0,688
18				0,614	-0,984	0,477
19				0,737	-0,702	0,508
20				0,408	-0,645	0,304
21				0,580	-1,160	0,386
23		0,770			-1,336	0,511
24		0,588			-0,350	0,380
25				0,718	-0,925	0,442
26				0,760	-0,390	0,562
27				0,494	-1,185	0,370
31				0,674	-1,074	0,470
32				0,455	-1,363	0,274
33				0,729	-0,681	0,322
36				0,735	-1,102	0,570
37	0,763				-1,167	0,718
38				0,522	-1,051	0,376
39				0,465	-1,010	0,289
41		0,792			-0,085	0,621
42		0,547			1,233	0,391
43		0,806			-1,436	0,651
45		0,763			-0,518	0,594
48				0,435	-1,198	0,230
FC	0,840	0,862	0,883	0,910		

*F1 - influência de terceiros; F2 - interferência do sujeito na terapêutica; F3 - letramento em medicamentos; F4 - atitudes e comportamentos; K - curtoses; h^2^ - comunalidades; FC - fidedignidade composta.*

Os itens apresentaram cargas fatoriais adequadas, com cargas fatoriais elevadas nos respectivos fatores. Não se encontrou padrão de cargas cruzadas (itens com cargas fatoriais >0,300 em mais de um fator). Os fatores apresentaram alta confiabilidade, conforme valores de FC (F1=0,840; F2=0,862; F3=0,883 e F4=0,910, e =0,87).

A Tabela 2 especifica a qualidade e eficácia das estimativas de pontuações dos fatores do QAR-LS. O questionário se mostrou adequado quanto à precisão (0,79 < ORION < 0,98), representatividade (0,89 < FDI < 0,99), sensibilidade (1,92 <SR<7,07), espera do fator (88,3%< EPTD<97,9%) e repetibilidade (0,87<H-Latente< 0,94) (0,82 < H-observado<0,91). Todos os fatores se apresentaram adequados, conforme os valores de referências, atestando a qualidade.

**Tabela 2:** Qualidade das estimativas dos fatores do Questionário da Automedicação de Risco Focado no Letramento em Saúde (QAR-LS), Fortaleza, Ceará, Brasil, 2023

Fatores	ORION	FDI	SR	EPTD (%)	*G-H Index*	
					H-latente	H-observado
Influência de terceiros	0,870	0,933	2,591	91,2	0,87 (0,28-0,89)	0,82 (0,75-0,87)
Interferência do sujeito na terapêutica	0,886	0,941	2,792	91,9	0,88 (0,85-0,91)	0,80 (0,73-0,87)
Letramento em medicamentos	0,904	0,951	3,073	92,7	0,90 (0,86-0,92)	0,83 (0,75-0,86)
Atitudes e comportamentos	0,930	0,965	3,654	94,1	0,93 (0,92-0,94)	0,90 (0,88-0,91)

A Tabela 3 expõe os índices de ajuste dos modelos testados e revela a qualidade do modelo 2 sobre os demais, expressa pelos valores na AFE e AFC.

**Tabela 3:** Índices de ajuste do modelo, Fortaleza, Ceará, Brasil, 2023

Estimativas	Valor	IC95%	Valores de referência
X^2^/gl	1,83	-	≤ 5,0
*Tucker-Lewis Index*	0,985	0,986-0,992	> 0,90
*Comparative Fit Index*	0,988	0,989-0,994	> 0,94
*Goodness of Fit Inde*	0,951	0,946-0,961	> 0,95
*Adjusted Goodness of Fit Index*	0,937	0,931-0,950	> 0,93
*Root Mean Square Error of Approximation*	0,027	0,019-0,027	< 0,07
*Root Mean Square of Residuals*	0,052	0,048-0,052	< 0,08

Tendo em vista os resultados obtidos, o Quadro 1 apresenta a versão final, após análises fatoriais, em que o QAR-LS apresenta 35 itens divididos em quatro dimensões (influência de terceiros; interferência dos sujeitos na terapêutica; letramento em medicamentos; e atitude e comportamentos).

**Quadro 1:** Versão final do Questionário da Automedicação de Risco Focado no Letramento em Saúde (QAR-LS), Fortaleza, Ceará, Brasil, 2023

	QAR-LS (Questionário de Automedicação de Risco focado no Letramento em Saúde)
**Influência de terceiros**	
1	Antes de usar remédio por conta própria, busco informações com amigos/familiares.
2	Durante o uso de remédio(s) por conta própria, ao sentir reação do medicamento, busco informações com familiares/amigos.
3	Tenho intenção de tomar remédio por conta própria porque confio na opinião de amigos/vizinhos/familiares.
4	Uso remédio por conta própria, por ser recomendado por amigos/vizinhos/familiares.
**Interferência do sujeito na terapêutica**	
5	Eu teria intenção de parar o uso do remédio receitado pelo médico se os sintomas melhorassem.
6	Eu teria intenção de parar o uso do remédio receitado pelo médico para usar em outro momento.
7	Diminuo a dose do remédio que o médico ou enfermeiro receitou, quando os sintomas melhoram.
8	Aumento a dose do remédio que o médico ou enfermeiro receitou, quando percebo que não estou melhorando.
9	Paro de usar o remédio por conta própria, quando me sinto melhor.
10	Já reduzi a quantidade de dias do tratamento.
**Letramento em medicamentos**	
11	Antes de usar remédio por conta própria, leio as informações da bula.
12	Antes de usar remédio por conta própria, leio as informações da embalagem/rótulo.
13	Antes de usar remédio por conta própria, eu avalio a dose que devo tomar.
14	Antes de usar remédio por conta própria, tiro minhas dúvidas na bula.
15	Antes de usar remédio por conta própria, vejo na bula o número de dias do tratamento.
16	Ao tomar remédio por conta própria, eu sei olhar na bula a dose correta.
17	Ao tomar remédio por conta própria, compreendo a bula.
**Atitudes e comportamentos**	
18	Antes de usar remédio por conta própria, sigo minha experiência de já ter tomado antes aquele remédio.
19	Antes de usar remédio por conta própria, sigo receitas anteriores.
20	Tenho intenção de tomar remédio por conta própria porque alivia rápido os sintomas.
21	Tenho intenção de tomar remédio por conta própria porque o medicamento faz efeito.
22	Tenho intenção de tomar remédio por conta própria, quando não precisa de consulta médica.
23	Tenho intenção de tomar remédio por conta própria porque os tenho em casa.
24	Tenho intenção de tomar remédio por conta própria, mesmo sem saber identificar as reações do medicamento.
25	Tenho intenção de tomar remédio por conta própria, mesmo sem aconselhamento médico, farmacêutico ou de enfermeiro.
26	Tomei remédios por conta própria, nos últimos três meses.
27	Uso remédio por conta própria quando não preciso de receita na farmácia.
28	Uso remédio por conta própria, mesmo quando a receita é exigida na farmácia.
29	Uso remédio por conta própria porque tenho experiências de tratamento anteriores.
30	Uso remédio por conta própria porque tenho receitas antigas.
31	Uso remédio por conta própria porque tenho remédios guardados em casa.
32	Uso remédio por conta própria, por ser mais rápido para resolver meus problemas de saúde.
33	Uso remédio por conta própria, quando não precisa de orientação(ões) de profissionais de saúde.
34	Uso mais de um remédio por conta própria ao mesmo tempo.
35	Uso antibióticos por conta própria.

Os indicadores avaliados apontam modelo de quatro dimensões com evidência interna consistente para mensuração do construto AR.

## DISCUSSÃO

Instrumentos de avaliação da automedicação fornecem informações relevantes à compreensão da prevalência de automedicação na população. Porém, o desenvolvimento de métricas padronizadas de autorrelato, sensíveis em relação ao conhecimento, à intenção e ao comportamento da AR, amplia a compreensão dos elementos de risco à saúde do sujeito e auxilia o serviço de saúde a usar intervenções efetivas que possam minimizar os danos da automedicação inadequada à saúde humana^(29)^.

O QAR-LS se mostra instrumento de rastreio útil e vantajoso, pois considera a influência de terceiros, interferência dos sujeitos na terapêutica, letramento em medicamentos e atitude e comportamentos, podendo ser utilizado nas estratégias dos serviços de saúde para minimizar elementos potenciais de riscos à saúde da população. Para minimizar riscos à saúde e garantir uso seguro de medicamentos, é necessário que o indivíduo tenha conhecimento, atitude e prática adequada em relação ao uso de medicamentos sem receita^(30)^.

Os resultados do presente estudo demonstraram que o QAR-LS apresenta características multidimensionais, cargas fatoriais satisfatórias e bons níveis de confiabilidade, que apontam para um instrumento com evidência interna consistente e confiável para medir o construto desejado. Até o momento, este é o primeiro estudo a avaliar a validade da AR, na perspectiva do letramento em saúde (letramento em medicamentos) e da Teoria do Comportamento Planejado, usando várias técnicas de dimensionamento e índices de ajuste.

Reforça-se que a literatura tem demonstrado relação entre a prática inadequada da automedicação e o baixo letramento em medicamentos^(9,31)^, assim como tem sido demonstrado que os construtos da Teoria do Comportamento Planejado (atitude, norma subjetiva e controle percebido) são fortes preditores da automedicação^(32-33)^.

A validade baseada na estrutura interna de um instrumento representa o grau em que a estrutura de correlações entre os itens corresponde à estrutura do construto que o teste propõe mensurar^(34)^. Portanto, avaliar as evidências de estrutura interna de instrumentos é um procedimento complexo que requer execução de requisitos, como a estrutura fatorial do instrumento e a confiabilidade, determinantes para que seja efetivada^(16)^. Neste estudo, a estrutura empírica do instrumento refletiu a estrutura teórica do construto, e a amostra analisada se mostrou adequada e representativa para continuidade da análise fatorial, conforme evidências dos BTS, KMO e RCI.

A análise fatorial identificou que o construto AR se organiza em quatro fatores, evidenciados pela matriz de correlação da análise fatorial como congruentes com a definição de AR. A estrutura teórica se apresentava com três fatores: letramento em medicamentos; intenção do comportamento; e comportamento. Dessa forma, com o reagrupamento dos itens na análise fatorial, redefiniram-se os quatro fatores em: influência de terceiros; interferência dos sujeitos na terapêutica; letramento em medicamentos; e atitude e comportamentos.

Os quatro fatores, evidenciados pela matriz de correlação da análise fatorial, foram congruentes com a definição de AR e os respectivos elementos constituintes: AR é multifatorial e incorpora atributos comportamentais, intenção do comportamento (atitude, norma subjetiva e controle percebido) e letramento em medicamentos. Esse modelo rompe com o tradicional, buscando-se medir apenas a automedicação em geral. Dessa forma, mede-se AR focado no letramento em saúde em níveis que se possam adotar medidas preventiva e promocionais que antecedem ao risco da automedicação.

O fator 1 contém os itens das três dimensões teórica do construto AR; nesse fator, ancora-se, de modo mais preponderante, a Teoria do Comportamento Planejado, com o construto norma subjetiva, que é a crença acerca da pressão social exercida sobre um indivíduo para realizar ou não determinado comportamento e da motivação da pessoa em concordar com essa referida pressão^(35)^. Essa pressão de terceiros encontra pessoas que apresentam influências na decisão, motivação e prática da automedicação, como familiares^(36-37)^ e amigos^(5,38-39)^. Reforça-se a isso que estudo com base na Teoria do Comportamento Planejado evidenciou que 69% de esposos ou amigos influenciaram a tomada de medicamentos sem receitas^(5)^.

O fator 2 corresponde teoricamente ao letramento em medicamentos, à intenção de comportamento e ao comportamento na interferência da condução da terapêutica, apresentando as três dimensões do modelo teórico. Salienta-se que a automedicação é um processo que envolve diversas facetas, como a definição da automedicação da OMS^(1)^ como uso intermitente, quando se configura a interrupção/interferência na condução do tratamento prescrito pelo clínico. A intenção do comportamento e da prática de realizar essa situação, aliada ao abaixo letramento, constitui potencial risco à saúde. Destarte, a interrupção do tratamento, por exemplo, do uso de antibiótico, pode contribuir para resistência bacteriana^(40-41)^.

O fator 3 se refere a itens considerados exclusivamente do letramento de medicamentos, como ler e compreender rótulos e bulas dos medicamentos, para garantir uso seguro da dose, duração e entre outros aspectos que garantem a segurança do paciente. Desse modo, esses itens se configuram como inversos ao construto AR, cujas pessoas que marcarem baixo na escala de Likert estarão propensas a um maior risco ao realizar a automedicação.

O fator 4, intenção do comportamento e comportamento da AR, envolve a motivação do sujeito em realizar determinado comportamento, como acondicionar medicamentos em casa ou mesmo a prática em si de já ter acondicionado medicamentos outras situações, como usar antibiótico por contra própria e não buscar informação com os profissionais de saúde. Esses elementos são potenciais riscos para condução do uso do medicamento por conta própria.

Os fatores demonstraram forte consistência interna, de acordo com valor de FC. Neste estudo, optou-se pelo uso da FC, pois constitui técnica que considera a carga fatorial dos itens, logo, permitindo avaliar melhor a qualidade do modelo estrutural de instrumentos psicométrico^(42)^.

Os índices de adequação de ajuste foram satisfatórios, sugerindo aceitação do modelo. Desse modo, o uso de técnicas robustas realizadas pela análise fatorial confirmatória, como precisão, representatividade, sensibilidade, replicabilidade, porcentagem esperada do fator e índices de adequação do modelo, além do uso desses indicadores, aliados à interpretação teórica, tornou possível eliminar possíveis vieses.

O instrumento pode auxiliar no preenchimento de lacuna relacionada à escassez de outras formas de avaliação das ações focadas na AR. Ademais, pode ajudar aos trabalhadores de saúde a elaborarem intervenções, podendo atuar em diferentes esferas, de forma a promover a saúde da população.

### Limitações do estudo

Entretanto, algumas limitações incluem possíveis fatores culturais que podem impactar a compreensão dos itens, a subjetividade nas respostas dos participantes e a não generalização para diferentes populações, sendo importante a realização de futuros estudos que envolvam participantes de outras regiões, com outras culturas e outros comportamentos. Ademais, a pesquisa traz técnicas robustas fundamentadas nas recomendações da psicometria.

### Contribuições para as áreas da enfermagem, saúde ou políticas públicas

Este estudo oferece instrumento valioso para preencher lacuna na avaliação da AR, fornecendo ferramenta que pode orientar os profissionais de saúde na elaboração de intervenções direcionadas a grupos específicos. O instrumento destaca aspectos como letramento em medicamentos, intenção de comportamento e tomada de decisão, abordando atitudes, normas subjetivas e controle percebido relacionados à prática da AR. Isso possibilita avaliar o conhecimento, a decisão e a prática da AR, permitindo intervenções direcionadas e eficazes para promover a saúde daqueles em situação de risco em relação à automedicação.

## CONCLUSÕES

O QAR-LS, composto por 35 itens, distribuídos em quatro dimensões (influência de terceiros, interferência dos sujeitos na terapêutica, letramento em medicamentos e atitude e comportamentos), apresentou propriedades psicométricas satisfatórias, evidenciando modelo com boas evidências de validade e confiabilidade.

## Data Availability

https://doi.org/10.48331/scielodata.WPYUK2
